# N‑Heterocyclic Carbene Stabilized Aluminum Alkyls and Their Reactivity toward NHC-Alanes

**DOI:** 10.1021/acs.organomet.5c00409

**Published:** 2025-12-14

**Authors:** Stuart Burnett, Alan R. Kennedy, Catherine E. Weetman

**Affiliations:** Department of Pure and Applied Chemistry, 3527 University of Strathclyde, 295 Cathedral Street, Glasgow G1 1XL, United Kingdom

## Abstract

Herein, we report the synthesis of several new NHC-stabilized aluminum alkyl species [NHCAlR_3_] (for NHC = ICy, R = Me, *i*Bu; NHC = IMes and IDip, R = *i*Bu) via coordination of the respective free carbene to AlMe_3_ or Al*i*Bu_3_. Attempts to access Al–Al bonded species (dialumanes) via reactions with the respective NHCAlH_3_ complexes did not yield the desired Al­(II) complexes via R-H elimination, instead yielding the R/H ligand scrambled complexes NHCAlR_2_H and NHCAlRH_2_, respectively. These mixed-ligand species were characterized by ^1^H, ^13^C­{^1^H}, and multinuclear NMR spectroscopy, with select cases characterized by single-crystal X-ray diffraction studies.

## Graphical abstract

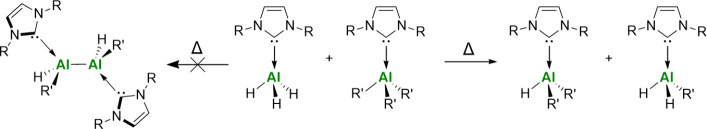

## Introduction

Over the last few decades, interest in the synthesis and reactivity of low oxidation-state aluminum species has surged. Landmark contributions in this area include Schnöckel’s tetrameric [(Cp*Al)_4_] (Cp* = pentamethylcyclopentadiene);^1^ Roesky’s monomeric, β-diketiminate stabilized Al­(I) [(^Dip^NacNac)­Al] **1** (^Dip^NacNac = HC­(CMeN­(Dip)); Dip = 2,6-diisopropylphenyl);^2^ the isolation of nucleophilic Al­(I) complexes by Aldridge and Goicoechea in 2018;^3^ and monocoordinated, neutral Al­(I) complexes from the Power^4^ and the Liu^5^ groups, respectively. Uhl's use of the bulky bistrimethylsilylmethyl ligand [CH­(SiMe_3_)_2_]^−^ enabled the synthesis of the first crystallographically characterized dialumane containing an Al–Al single bond.^6^ Since then, a plethora of Al­(II) complexes featuring Al–Al bonds have been reported using a variety of bulky stabilizing ligands, including terphenyls pioneered by the Power group,^7–12^ diimido-based ligands by the groups of Yang and Fedushkin,^13–19^ and Cp ligands utilized by the Braunschweig and Arnold groups.^20–22^

Despite these advances, the synthesis of low oxidation-state aluminum complexes has predominantly relied on the use of strong alkali metal-based reducing agents. Recently, several examples have demonstrated the feasibility of using alternative main group of reducing agents to yield such species. Jones and Stasch isolated the first examples of stable, neutral aluminum­(II) hydride complexes (Figure 1a),^23^ including the first example of a *N*-heterocyclic carbene (NHC) adduct of the parent dialane(4) via reduction of the Al­(III) hydride species using Mg­(I) (Mg­(I) = [{(^Ar^NacNac)­Mg}_2_] (Ar = Dip **2a**, Mes **2b**)). Similarly, Cowley and co-workers utilized Mg­(I) to prepare a series of amidophosphine stabilized dihydrodialanes,^24^ whereby varying the sterics of the donor ligand influences the Al–Al bond length and allowed for the detection of reversible reductive elimination processes. In 2014, Nikonov demonstrated that Roesky’s Al­(I) could act as a stoichiometric reducing agent in reactions with a β-diketiminate stabilized Al­(III) dihydride complex (Figure 1b),^25^ affording the corresponding dihydrodialane [{(^Dip^NacNac)­AlH}_2_], albeit existing in equilibrium with the aforementioned Al­(I) and Al­(III) starting materials. Building upon this, the Bakewell group introduced a series of symmetric and asymmetric dihydrodialanes, along with a masked dialumene species, via similar Al­(I) reduction of amidinate and β-diketiminate Al­(III) dihydrides.^26^ More recently, our group has similarly investigated the use of both Mg­(I) and Al­(I) as stoichiometric reducing agents toward various NHC-alanes (Figure 1c).^27^ Reactions with Mg­(I) dimer (**2b**) exclusively yielded the expected dialane complexes [{NHCAlH_2_}_2_] (NHC = IPr*, IDip, ICy), while use of Al­(I) gave rise to, depending on the steric demand of the NHC, an NHC-dialane (with IPr*), a cationic abnormal aluminum dihydride (with IDip), or an asymmetric mixed-ligand dialane (with ICy).

**Figure 1:**
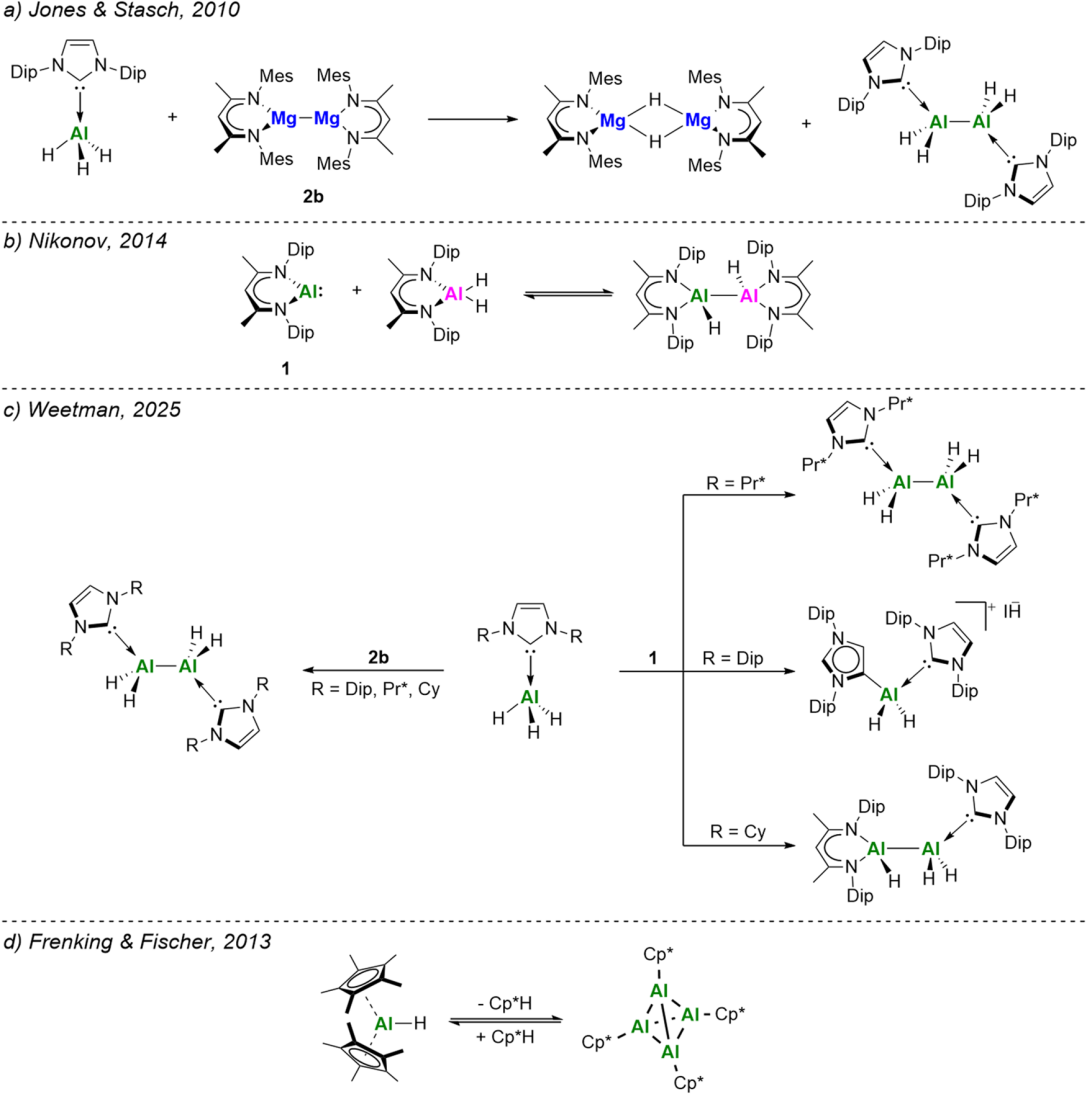
(a–c) Reported usage of low oxidation-state main group complexes as reducing agents. (d) Synthesis of [(Cp*Al)_4_] via thermally induced reductive elimination.

Although NHC-stabilized aluminum complexes have been known for some time (*vide infra*), research on this compound class has grown significantly in recent years. In 2017, the Inoue group reported the first example of an NHC-stabilized, neutral Al=Al doubly bonded species,^28^ which was shown to initiate both stoichiometric and catalytic reduction of CO_2_.^29^ The Radius group has reported on various NHC-ligated Al­(III) and Al­(II) species,^30–32^ with a particular emphasis on the application of these compounds in ring opening/expansion^33^ and decarbonization of carbene ring systems.^34^ While catalytic applications of NHC–aluminum species remain limited to two reported cases—amine–borane dehydrocoupling^35,36^ and the aforementioned CO_2_ reduction—the prospect of employing aluminum in catalysis is compelling, given its natural abundance and low cost. As such, exploration of alternative synthetic routes to obtaining reactive, low oxidation-state aluminum species represents a worthwhile goal.

While recent advancements with stoichiometric main group reducing agents has opened new avenues in low oxidation-state aluminum chemistry, most known systems still rely on classical alkali metal-based reductants, and further work investigating alternative routes is still needed. Inspired by a 2013 report by Fischer et al.,^37^ where they reported a novel synthetic route to [(Cp*Al)_4_] via thermally induced reductive elimination of Cp*H from Cp*_2_AlH (Figure 1d), we sought to extend this methodology toward the synthesis of NHC-stabilized Al­(II) complexes, specifically investigating reactions of NHC-stabilized aluminum­(III) alkyls and hydrides in an effort to yield dialumanes without the use of external reducing agents.

## Results and Discussion

First reported by Arduengo in 1992,^38^ NHC-aluminum hydride complexes are typically synthesized via addition of the respective free NHC to solutions of Me_3_N·AlH_3_.^39^ Formation of NHC-aluminum alkyl complexes typically follows a similar route (Figure 2).^40,41^

**Figure 2:**
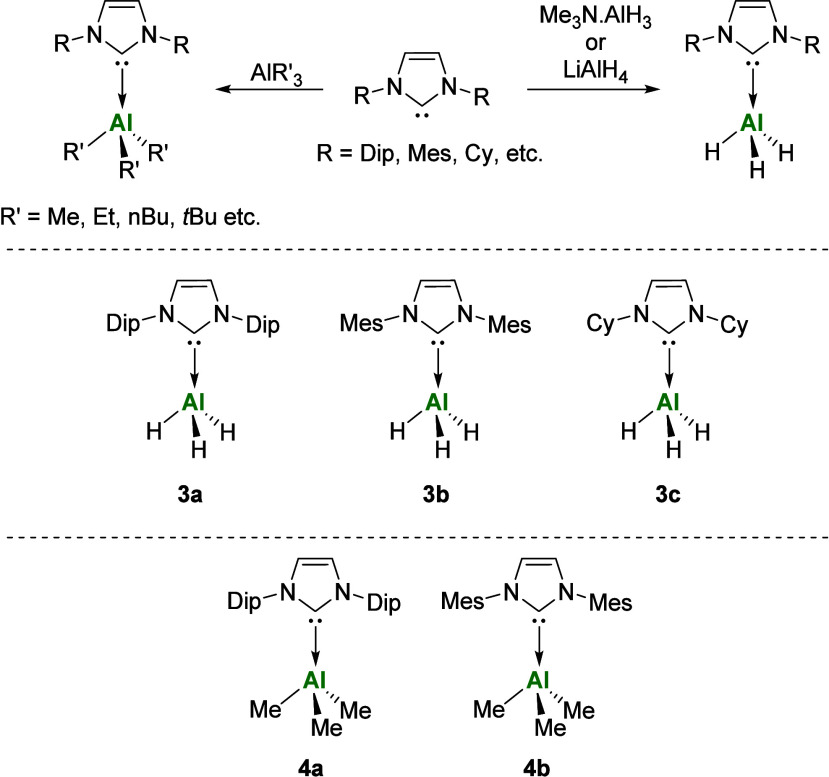
Reported synthesis of the NHCAlH_3_ and NHCAlR_3_ complexes (top). Selected examples of NHCAlH_3_ (middle) and NHCAlR_3_ (bottom) complexes relevant to this work.

This method was employed to extend the library of reported NHCAlR_3_ complexes. Addition of Al*i*Bu_3_ to hexane solutions of the free NHC’s IDip, IMes, and ICy affords the corresponding aluminum alkyl adducts IDipAl*i*Bu_3_ **5a**, IMesAl*i*Bu_3_ **5b**, and ICyAl*i*Bu_3_ **5c**, respectively (Scheme 1). Similarly, the addition of AlMe_3_ to solutions of ICy affords the expected ICyAlMe_3_ **4c** complex.

**Scheme 1:**
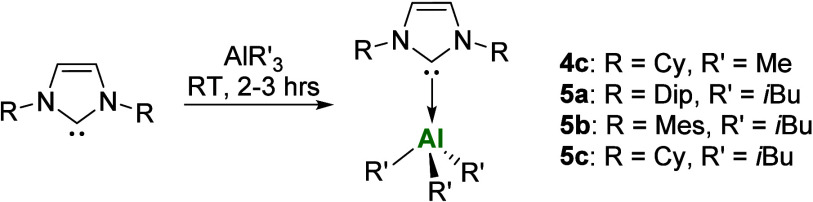
Synthesis of NHC-Aluminum Alkyl Complexes **4c** and **5a–c**

**5a** and **5b** were isolated as colorless crystalline solids from concentrated hexane solution in excellent yields of 77 and 79%, respectively, while **4c** and **5c** were isolated as red/orange solids in lower yields of 29 and 41%, respectively. Complexes **4c** and **5a**–**c** were spectroscopically characterized by ^1^H and ^13^C­{^1^H} NMR spectroscopy, exhibiting resonances in agreement with their expected structure. The ^13^C­{^1^H} carbenic carbon resonances for **5a**–**c** were all extremely weak while **4c** was not observed at all; however, this can be assigned via 2D NMR methods (Figures S4 and S5). The carbenic carbon resonances (**4c**: 173.9 ppm; **5a**: 181.8 ppm; **5b**: 179.3 ppm; **5c**: 173.9 ppm; cf. IDipAlMe_3_ **4a**: 181.1 ppm; IMesAlMe_3_ **4b**: 178.5 ppm) fall expectedly upfield from the respective free carbene resonances consistent with coordination to an aluminum center (cf. IDip 220.6 ppm;^42^ IMes 219.7 ppm;^43^ ICy 212.0 ppm^44^). The downfield trend in the Al-CH_2_ chemical shifts from **5a**–**c** (**5a**: −0.35 ppm, **5b**: −0.28 ppm, **5c**: 0.47 ppm) indicates weaker σ-donation and lower basicity of the ligand from IDip > IMes > ICy. This trend was similarly noted by Garcia^41^ and Barron^45,46^ for related NHC- and phosphine-stabilized aluminum complexes.

Complex **4c** displays an Al-CH_3_ resonance at −0.10 ppm, following the trend mentioned above (IDipAlMe_3_ **4a**: −0.86 ppm; IMesAlMe_3_ **4b**: −0.78 ppm). Both ICy-stabilized species **4c** and **5c** have notable downfield shifts of the Al–C*H* substituent compared with free AlMe_3_ (−0.35 ppm)^41^ and Al*i*Bu_3_ (0.29 ppm),^47^ respectively. This is surprising as coordination to the Lewis basic ICy would typically result in an upfield shift due to the σ-donor nature of the ligand; however, this can be explained by intramolecular hydrogen bonding between the Al–C*H* and the ligand N–C*H* as confirmed by NOESY NMR spectroscopy for **4c** and **5c** (Figures S6 and S22). Additionally, the Al–C*H* and N–C*H* distances in the solid-state structures (e.g., **4c**: 2.216 Å, 2.296 Å; **5c**: 2.110 Å, 2.283 Å) are shorter than the combined van der Waals radii indicating a strong interaction in both cases.^48^

Complexes **4c** and **5a**–**c** show good solution state thermal stability—heating benzene-*d*_6_ solutions to 100 °C for 3 days shows no noticeable decomposition of the respective compounds. Solid-state structures of **4c**, **5b**, and **5c** were obtained via single-crystal X-ray diffraction (SC-XRD) analysis (Figure 3). The C_carbene_-Al bond lengths (**4c**: 2.0860(3) Å; **5b**: 2.1337(12) Å; and **5c**: 2.1075(17) Å) are consistent with previously reported NHC-stabilized aluminum alkyl complexes^41^ (cf. for IDipAlMe_3_ **4a** C_carbene_-Al: 2.1030(3) Å; IMesAlMe_3_ **4b** C_carbene_-Al: 2.0984(16) Å). As expected upon NHC-coordination, the Al–C_R_ bond lengths (**4c**: 1.997(3), 1.999(3), and 2.003(3) Å; **5b**: 2.0139(12), 2.0203(12), and 2.0018(12) Å; **5c**: 2.0163(17), 2.014(2), and 2.009(2) Å) increase in comparison with uncoordinated AlMe_3_ (1.957(3) Å)^49^ and the terminal Al–C bonds in dimeric Al*i*Bu_3_ (1.972(3) and 1.983(4) Å).^47^

**Figure 3:**
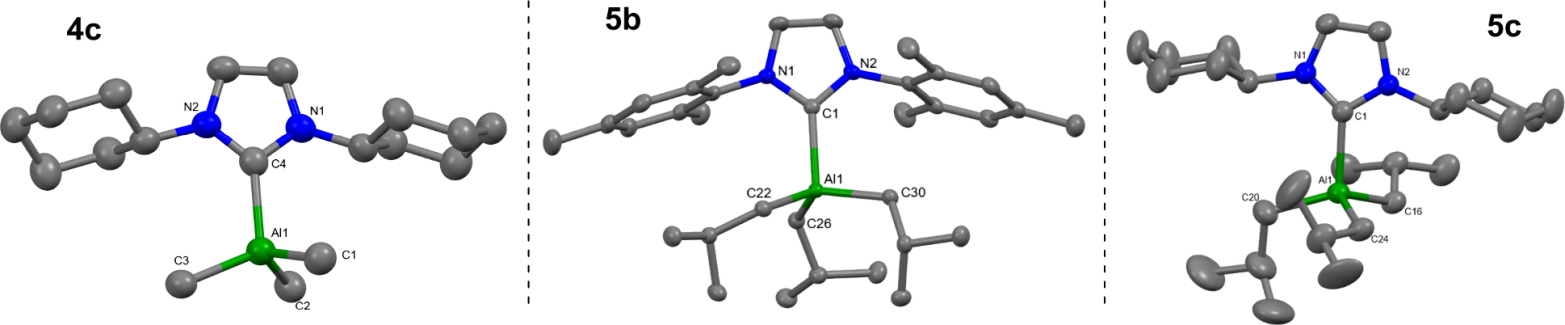
Molecular structures (50% thermal ellipsoids) of **4c** (left), **5b** (middle), and **5c** (right). Only one molecule of **4c** is shown. Hydrogen atoms and minor disorder in **5c** have been omitted for clarity. Selected bond lengths (Å) and angles (°): Complex **4c**: Al1–C1: 1.997(3), Al1–C2: 1.999(3), Al1–C3: 2.003(3), Al1–C4: 2.086(3), Al2–C19: 2.005(3), Al2–C20: 2.002(3), Al2–C21: 1.996(3), Al2–C22: 2.086(3); N1–C4: 1.368(3), N2–C4: 1.359(3); C1–Al1–C4: 107.27(12), C2–Al1–C4: 102.79(12), C3–Al1–C4: 110.37(12). Complex **5b**: Al1–C1:2.1337(12), Al1–C22: 2.0139(12), Al1–C26: 2.0203(12), Al1–C30: 2.0018(12), N1–C1: 1.3615(15), N2–C1: 1.3647(15); C22–Al1–C1: 109.19(5), C26–Al1–C1: 100.72(5), C30–Al1–C1: 101.69(5). Complex **5c:** Al1–C1: 2.1075(17), Al1–C16: 2.0163(17), Al1–C20: 2.014(2), Al1–C24: 2.009(2), N1–C1: 1.356(2), N2–C1: 1.363(2); C1–Al1–C16: 102.83(7), C1–Al1–C20: 110.33(8), C1–Al1–C24: 105.98(8).

Targeting dialumane formation, **4a**–**c** and **5a**–**c** were combined with the corresponding NHCAlH_3_ complex **3a**–**c** in a J. Young NMR tube. No change in the ^1^H NMR spectra was noted in any of the reactions over the course of 3 days at ambient temperature. Subsequent heating of reaction mixtures to 100 °C and monitoring by ^1^H NMR spectroscopy shows conversion of starting materials to at least two new NHC-containing species. For the least sterically hindered ICy reactions (**3c** + **4c** and **3c** + **5c**), no further conversion of starting materials is observed after 3 days at 100 °C, with approximate NMR conversions of 62% for **3c** + **4c** and 85% for **3c** + **5c**. Only slight shifts in the resonances attributed to the products from both reactions are observed in the resulting ^1^H NMR spectra, suggestive of solution-state structures similar to those of **3c**, **4c**, and **5c**. Particularly informative are the new Al-CH resonances observed in each reaction—for **3c** + **4c**, a new triplet at −0.05 ppm and a doublet at −0.08 ppm are present, while for **3c** + **5c**, three new doublets at 0.41, 0.53, and 0.58 ppm and a doublet of triplets at 0.61 ppm are now observed, all of which are suggestive of coupling between an Al-CH moiety and potentially a new Al–H group. SC-XRD analysis of colorless crystals grown from the reaction between **3c** and **5c** revealed the nature of one of these species. Instead of yielding the desired dialumane, the reaction in fact forms mixed alkyl/hydride complexes of the form ICyAl*i*Bu_2_H **7c** and ICyAl*i*BuH_2_ **7c′**, arising via thermally induced alkyl/hydride scrambling processes (Scheme 2). The molecular structure of **7c** showed poor-quality data precluding detailed structural analysis; however, it does provide confirmation of its connectivity (Figure 4).

**Scheme 2:**
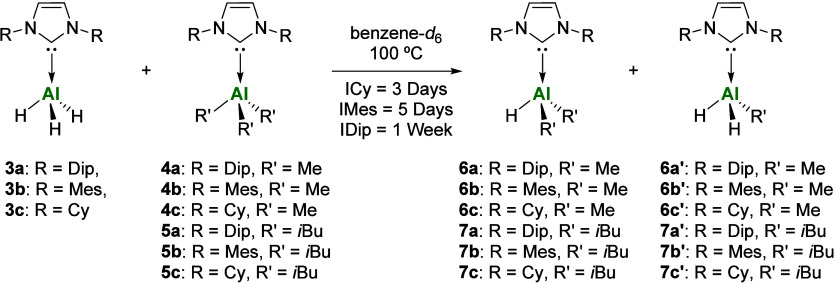
Synthesis of NHC-Aluminum Alkyl/Hydride Complexes

**Figure 4:**
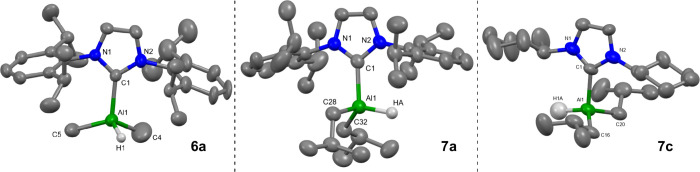
Molecular structures (50% thermal ellipsoids) of **6a** (left), **7a** (middle), and **7c** (right). Only one molecule of **7a** is shown. Hydrogen atoms (except for Al–H groups) and minor disorder in **6a** and **7b** have been omitted for clarity. Selected bond lengths (Å) and angles (°): Complex **6a:** Al1–C1: 2.094(2), Al1–C4: 1.939(5), Al1–C5: 1.963(3), Al1–H1: 1.550(19), N1–C1: 1.358(3), N2–C1: 1.356(3); C1–Al1–H1: 110.9(7), C1–Al1–C4: 108.87(17), C1–Al1–C5: 111.47(10). Complex **7a:** Al1–C1: 2.101(4), Al–C28: 2.003(4), Al1–C32: 1.997(4), Al1-HA: 1.56(3), N1–C1: 1.361(5), N2–C1: 1.366(4); C1–Al1–HA: 103.9(12), C1–Al1–C28: 103.94(15), C1–Al1–C32: 114.28(16). Bond lengths for complex **7c** are included only for reference: Al1–H1A: 1.64(12), Al1–C1: 2.086(10), Al1–C16: 2.002(15), Al1–C20: 1.997(16).

The assignment of the ^1^H NMR spectrum from the reaction between **3c** and **4c** suggests similar mixed species, i.e., ICyAlMe_2_H **6c** and ICyAlMeH_2_ **6c′** are formed, with the triplet at −0.05 ppm arising from coupling between the lone Al-CH_3_ group and the two Al–H substituents in **6c′**, while the doublet at −0.08 ppm is assigned to the two Al-CH_3_ groups in **6c** arising from coupling to the adjacent Al–H. No resonances for the carbenic carbon centers in **6c** and **6c′** could be observed in the reaction mixture ^13^C­{^1^H} NMR spectrum, though weak coupling was observed in the ^1^H–^13^C HMBC NMR spectra at 172.8 and 173.7 ppm.

As expected, the more sterically demanding IMes- and IDip-stabilized reactions require extended heating times to achieve similar (approximate) conversion rates: IMes—**3b** + **4b**: 5 days, 82% conversion; **3b** + **5b**: 5 days, 98% conversion; IDip—**3a** + **4a**: 1 week, 76% conversion; **3a** + **5a**: 1 week, > 99% conversion. Similar Al-CH_3_ and Al-CH_2_ resonances were observed in the resulting ^1^H NMR spectra of these reactions as were observed for the ICy-stabilized mixtures, again suggestive of mixed alkyl/hydride aluminum species (see SI for full NMR assignment).

For both IDip reactions (**3a** and **4a**/**5a**), colorless crystals were grown from concentrated benzene-*d*_6_ reaction mixtures that were suitable for SC-XRD, yielding solid-state structures IDipAlMe_2_H **6a** and IDipAl*i*Bu_2_H **7a** (Figure 4). It is worth noting IDipAl*i*Bu_2_H **7a** has been previously synthesized by Radius and co-workers^33^ via addition of diisobutylaluminum hydride to free IDip. While this was never structurally characterized by X-ray diffraction, their solution-state NMR assignment matches one set of resonances obtained from the reaction of **3a** and **7a**. Both complexes crystallize in the monoclinic *P*2_1_/*c* space group, with **7a** containing 2 molecules in the asymmetric unit. Both contain distorted tetrahedral aluminum centers similar to that observed in **7c**. The observed C_carbene_-Al bond lengths in **6a** and **7a** are slightly shorter than the corresponding trialkyl species (**6a** 2.094(2) Å vs **4a** 2.103(3) Å;^41^ **7a** 2.101(4) Å vs **5a** 2.1337(13) Å), with the Al–H bond lengths (**6a** 1.550(19) Å; **7a** 1.560(3) Å) concurrently longer than those in IDipAlH_3_ **3a** (1.527(15), 1.546(17), and 1.510(17) Å).^50^

## Conclusions

In summary, we have synthesized a series of NHC-stabilized aluminum alkyl complexes and studied their reactivity with corresponding NHCAlH_3_ complexes. Instead of the intended R-H elimination to yield new dialumanes, thermally induced alkyl/hydride scrambling occurs yielding mixed NHC-aluminum complexes of the form NHCAlR_2_H and NHCAlRH_2_, respectively. These reaction mixtures have been studied by solution-state NMR spectroscopy, with select examples characterized by SC-XRD.

## Supplementary Material



## Notes

General experimental materials and methods, synthetic procedures for the preparation of all compounds, NMR spectra and X-ray diffraction data (PDF)
