# Supplementary material for: N‑Heterocyclic Carbene Stabilized Aluminum Alkyls and Their Reactivity toward NHC-Alanes
Source: Organometallics. 2025 Dec 14;45(1):90–5. doi: 10.1021/acs.organomet.5c00409 (PMC12801380; doi:10.1021/acs.organomet.5c00409)
Supplement: Supplementary file 1 [file om5c00409_si_001.pdf]

N-Heterocyclic carbene stabilized aluminium alkyls and their reactivity towards NHC-alanes

Supporting Information

*Stuart Burnett, Alan R. Kennedy and Catherine E. Weetman**

Department of Pure and Applied Chemistry, University of Strathclyde, 295 Cathedral Street, Glasgow, G1 1XL, U.K.

*Corresponding author. Email: catherine.weetman@strath.ac.uk

Table of Contents

1. **General Experimental**
2. **Synthetic Procedures**
3. **NMR Spectroscopy**
4. **X-Ray Crystallography Data**
5. **References**

1. General Experimental

All manipulations were carried out using standard Schlenk and glove box techniques under a dry argon or dinitrogen atmosphere. Toluene and hexane were taken from an Innovative Technology Solvent Purification system and stored over activated 4Å molecular sieves. Pentane was dried by distillation over sodium benzophenone and stored over activated 4Å molecular sieves prior to use. Benzene-d_6 was purchased from Sigma-Aldrich and stored over activated 4Å molecular sieves. All other reagents were purchased from Sigma-Aldrich, Fluorochem or Alfa Aeser and used as received.

NMR spectra were recorded on a Bruker AV3-400 spectrometer operating at 400.4 MHz (^{1}H) and 100.7 MHz ($^{13}C\{^{1}H\}$) and were measured at 298K. All chemical shifts are expressed in parts per million (δ, ppm) and were referenced to the residual ^{1}H or $^{13}C\{^{1}H\}$ resonances of the solvent used. ICy[1], IMes[2], IDip[3], $ICyAlH_3$ **3c**[4], $IMesAlH_3$ **3b**[4], $IMesAlMe_3$ **4b**[5], $IDipAlH_3$ **3a**[6], $IDipAlMe_3$ **4a**[5] and $Me_3N.AlH_3$[7] were synthesised according to literature procedure. Elemental analyses were collected using a Thermo Scientific FlashSmart CHNS Elemental Analyzer in the Department of Civil and Environmental Engineering at the University of Strathclyde.

2. Synthetic Procedures

2.1 $ICyAlMe_3$ 4c

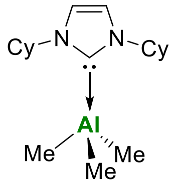

ICy (100 mg, 0.43 mmol) was added to a J. Youngs flask and slurried in dry hexane (6 mL). $AlMe_3$ (2M, 0.22 mL, 0.43 mmol) was then added dropwise at ambient temperature, resulting in complete dissolution of all starting material and a colour change from orange to red. The reaction mixture was subsequently stirred for 2 hours,

after which all volatiles were removed *in vacuo* affording **4c** as a burgundy red solid. Recrystallisation from concentrated hexane solutions afforded colourless crystals suitable for X-ray diffraction analysis. Yield = 38 mg (29%).

^{1}H NMR (benzene-d_6): δ = -0.10 (s, 9H, Al(CH_3)$_3$), 0.83 (q of t, $^{2}J_{HH}$ = 3.9 Hz, $^{3}J_{HH}$ = 12.8 Hz, 2H, Cy-H_2), 1.05 (q of d, $^{2}J_{HH}$ = 3.6 Hz, $^{3}J_{HH}$ = 12.5 Hz, 4H, Cy-H_2), 1.28 (q of t, $^{2}J_{HH}$ = 3.3 Hz, $^{3}J_{HH}$ = 13.4 Hz, 4H, Cy-H_2), 1.38-1.43 (m, 2H, Cy-H_2), 1.46-1.52 (m, 4H, Cy-H_2), 1.79-1.85 (m, 4H, Cy-H_2), 4.84 (t of t, J_{HH} = 3.8 Hz and 12.0 Hz, 2H, Cy-*H*), 6.30 (s, 2H, NC*H*C*H*N).

^{13}C{^{1}H} NMR (benzene-d_6): δ = 25.4 (Cy-*C*H$_2$), 25.5 (Cy-*C*H$_2$), 34.5 (Cy-*C*H$_2$), 57.9 (Cy-*C*H), 116.8 (N*C*H*C*HN). No resonance attributable to the carbenic carbon or the Al-(CH_3)$_3$ group could be observed.

^{27}Al NMR (benzene-d_6): No signal observed.

Elemental Analysis (%) for $C_{18}H_{33}AlN_2$: Calculated: C 71.01, H 10.93, N 9.20; Found: C 69.55, H 10.57, N 8.67.

2.2 IDipAl*i*Bu$_3$ 5a

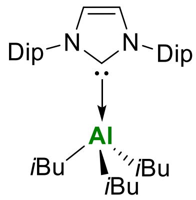

IDip (68 mg, 0.18 mmol) was added to a J. Youngs flask and slurried in dry hexane (6 mL). Al*i*Bu$_3$ (1M, 0.18 mL, 0.18 mmol) was then added dropwise at ambient temperature, resulting in complete dissolution of all starting material and formation of a colourless solution. The reaction mixture was subsequently stirred for 3 hours, after which all volatiles were removed *in vacuo* affording **5a** as a white solid. Efforts to obtain crystalline sample suitable for X-ray crystallographic analysis were unsuccessful. Yield = 79 mg (77%).

¹H NMR (benzene-d_6): δ = -0.35 (d, J_{HH} = 6.9 Hz, 6H, AlCH_2CH(CH_3)$_2$), 0.94 (d, J_{HH} = 6.9 Hz, 12H, Ar-CH(CH_3)$_2$), 1.14 (d, J_{HH} = 6.5 Hz, 18H, AlCH_2CH(CH_3)$_2$), 1.44 (d, J_{HH} = 6.8 Hz, 12H, Ar-CH(CH_3)$_2$), 2.00 (sept, J_{HH} = 6.5 Hz, 3H, AlCH_2C*H*(CH_3)$_2$), 2.72 (sept, J_{HH} = 6.8 Hz, 4H, Ar-CH(CH_3)$_2$), 6.40 (s, 2H, NC*H*C*H*N), 7.11-7.16 (m, 4H, Ar-*H*), 7.24-7.28 (m, 2H, Ar-H).

¹³C{¹H} NMR (benzene-d_6): δ = 22.9 (Ar-CH(*C*H_3)$_2$), 23.4 (Al*C*H_2CH(CH_3)$_2$), 26.1 (Ar-CH(*C*H_3)$_2$), 27.3 (AlCH_2*C*H(CH_3)$_2$), 28.8 (Ar-*C*H(CH_3)$_2$), 29.2 (AlCH_2CH(*C*H_3)$_2$), 124.2 (Ar-*C*), 124.6 (N*C*H*C*HN), 130.7 (Ar-*C*), 136.0 (Ar-*C*), 145.9 (Ar-*C*), 182.3 ($C_{carbene}$).

²⁷Al NMR (benzene-d_6): No signal observed.

Elemental Analysis (%) for $C_{39}H_{63}AlN_2$: Calculated: C 79.81, H 10.82, N 4.77; Found: C 79.61, H 9.97, N 4.85.

2.3 IMesAl*i*Bu₃ 5b

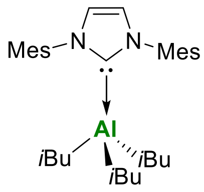

IMes (500 mg, 1.64 mmol) was added to a J. Youngs flask and slurried in dry hexane (10 mL). Al*i*Bu$_3$ (1M, 1.64 mL, 1.64 mmol) was then added dropwise at ambient temperature, resulting in complete dissolution of all starting material and a colour change from orange to red. The reaction mixture was subsequently stirred for 3 hours, after which concentrating the solution to *ca.* 8 mL and leaving at ambient temperature overnight afforded colourless crystals of **5b**. Further concentration of supernatant solutions afforded additional crops of **5b**. Crystals suitable for X-ray diffraction analysis were obtained from a concentrated hexane solution. Yield = 655 mg (79%).

^{1}H NMR (benzene-d_6): δ = -0.28 (d, J_{HH} = 6.9 Hz, 6H, AlCH_2CH(CH$_3$)$_2$), 1.17 (d, J_{HH} = 6.5 Hz, 18H, AlCH$_2$CH(CH_3)$_2$), 1.93 (sept, J_{HH} = 6.5 Hz, 3H, AlCH$_2$CH(CH$_3$)$_2$), 2.00 (s, 12H, Ar-*o*-CH_3), 2.14 (s, 6H, Ar-*p*-CH_3), 5.92 (s, 2H, NC*H*C*H*N), 6.77 (s, 4H, Ar-*H*).

^{13}C{^{1}H} NMR (benzene-d_6): δ = 18.0 (Ar-*o*-*C*H$_3$), 21.0 (Ar-*p*-*C*H$_3$), 23.1 (Al*C*H$_2$CH(CH$_3$)$_2$), 27.6 (AlCH$_2$*C*H(CH$_3$)$_2$), 29.4 (AlCH$_2$CH(*C*H$_3$)$_2$), 123.0 (N*C*H*C*HN), 129.5 (Ar-*C*), 135.4 (Ar-*C*), 135.8 (Ar-*C*), 139.6 (Ar-*C*), 179.6 ($C_{carbene}$).

^{27}Al NMR (benzene-d_6): No signal observed.

Elemental Analysis (%) for $C_{33}H_{51}AlN_2$: Calculated: C 78.84, H 10.23, N 5.57; Found: C 78.00, H 9.73, N 5.40.

2.4 ICyAl*i*Bu$_3$ 5c

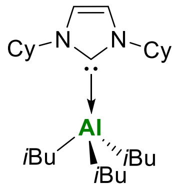

ICy (100 mg, 0.43 mmol) was added to a J. Youngs flask and slurried in dry hexane (6 mL). Al*i*Bu$_3$ (1M, 0.43 mL, 0.43 mmol) was then added dropwise at ambient temperature, resulting in complete dissolution of all starting material and a colour change from orange to red. The reaction mixture was subsequently stirred for 2 hours, after which all volatiles were removed *in vacuo* affording **5c** as a burgundy red solid. Recrystallisation from concentrated hexane solutions afforded colourless crystals suitable for X-ray diffraction analysis.

Yield = 76 mg (41%).

^{1}H NMR (benzene-d_6): δ = 0.47 (d, J_{HH} = 7.0 Hz, 6H, AlCH_2CH(CH$_3$)$_2$), 0.83-0.94 (m, 2H, Cy-H_2), 1.06-1.16 (m, 6H, Cy-H_2), 1.32 (d, J_{HH} = 6.5 Hz, 18H, AlCH$_2$CH(CH_3)$_2$), 1.37-1.48 (m, 8H, Cy-H_2), 1.55-1.60

(m, 4H, Cy-H_2), 1.91-1.94 (m, 4H, Cy-H_2), 2.20 (sept, J_{HH} = 6.6 Hz, 3H, $AlCH_2CH(CH_3)_2$), 4.78 (t of t, $^2J_{HH}$ = 3.7 Hz, $^3J_{HH}$ = 12.0 Hz, 2H, Cy-*H*), 6.30 (s, 2H, NC*H*C*H*N),

^{13}C{^{1}H} NMR (benzene-d_6): δ = 25.2 (Al*C*$H_2CH(CH_3)_2$), 25.4 (Cy-CH_2), 25.6 (Cy-*C*H_2), 28.2 ($AlCH_2$*C*$H(CH_3)_2$), 29.3 ($AlCH_2CH$(*C*$H_3)_2$), 34.7 (Cy-CH_2), 57.8 (Cy-*C*H), 117.3 (N*C*H*C*HN), 174.2 ($C_{carbene}$).

^{27}Al NMR (benzene-d_6): No signal observed.

Elemental Analysis (%) for $C_{27}H_{51}AlN_2$: Calculated: C 75.30, H 11.94, N 6.50; Found: C 74.87, H 11.58, N 6.35.

2.4 IDipAlMe$_2$H 6a and IDipAlMeH$_2$ 6a'

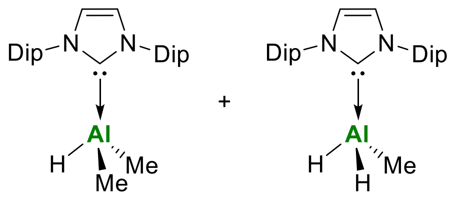

To a J. Young NMR tube was added $IDipAlMe_3$ (9.5 mg, 0.02 mmol) and $IDipAlH_3$ (8.6 mg, 0.02 mmol). Benzene-d_6 (0.5 mL) was then added and the tube sealed. The solution was subsequently heated to 100 ºC and the reaction was monitored by ^{1}H NMR spectroscopy. After 1 week of heating no further conversion was observed.

^{1}H NMR (benzene-d_6): δ = -0.95 - -0.96 (m, 6H and 3H, Al*C*H_3 **6a/6a'**), 1.01-1.03 (m, 12H and 12H, Ar-CH(CH_3)$_2$ **6a/6a'**), 1.40-1.42 (m, 12H and 12H, Ar-CH(CH_3)$_2$ **6a/6a'**), 2.74-2.82 (m, 4H and 4H, Ar-C*H*(CH$_3$)$_2$ **6a/6a'**), 6.46 (s, 2H and 2H, NC*H*C*H*N **6a/6a'**), 7.10-7.12 (m, 4H and 4H, Ar-*o*-CH **6a/6a'**), 7.22-7.26 (m, 2H and 2H, Ar-*p*-*H* **6a/6a'**).

^{13}C{^{1}H} NMR (benzene-d_6): δ = 23.0 (Ar-CH(*C*H$_3$)$_2$ **6a/6a'**), 25.6 (Ar-CH(*C*H$_3$)$_2$ **6a/6a'**), 29.0 (Ar-*C*H(CH$_3$)$_2$ **6a/6a'**), 29.1 (Ar-*C*H(CH$_3$)$_2$ **6a/6a'**), 124.0 (N*C*H*C*HN **6a/6a'**), 124.2 (Ar-*C* **6a/6a'**), 130.7

(Ar-*C* **6a/6a’**), 135.1 (Ar-*C* **6a/6a’**), 145.7 (Ar-*C* **6a/6a’**), 145.8 (Ar-*C* **6a/6a’**). No resonance attributable to the carbenic carbon or the Al-(CH_3)$_3$ groups of **6a** or **6a’** could be observed.

^{27}Al NMR (benzene-d_6): No signal observed.

2.5 IDipAl*i*Bu$_2$H 7a and IDipAl*i*BuH$_2$ 7a’

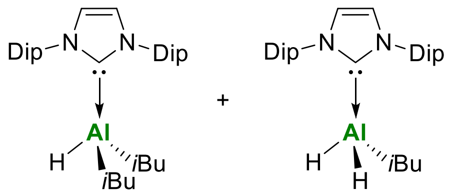

To a J. Young NMR tube was added IDipAl*i*Bu$_3$ (18 mg, 0.03 mmol) and IDipAlH$_3$ (13 mg, 0.03 mmol). Benzene-d_6 (0.5 mL) was then added and the tube sealed. The solution was subsequently heated to 100 ºC and the reaction was monitored by ^{1}H NMR spectroscopy. After 1 week of heating no further conversion was observed.

^{1}H NMR (benzene-d_6): δ = -0.63 (dd, $^2J_{HH}$ = 8.0 Hz, $^3J_{HH}$ = 13.4 Hz, 2H, AlC*H*$_2$CH(C*H*$_3$)$_2$ **7a**), -0.38 (dt, $^2J_{HH}$ = 2.2 Hz, $^3J_{HH}$ = 6.9 Hz, 2H, AlC*H*$_2$CH(C*H*$_3$)$_2$ **7a’**), -0.28 (dd, $^2J_{HH}$ = 2.9 Hz, $^3J_{HH}$ = 6.2 Hz, $^4J_{HH}$ = 13.4 Hz, 2H, AlC*H*$_2$CH(C*H*$_3$)$_2$ **7a**), 1.00 (d, J_{HH} = 6.9 Hz, 12H, Ar-CH(C*H*$_3$)$_2$ **7a**), 1.03 (d, J_{HH} = 6.9 Hz, 12H, Ar-CH(C*H*$_3$)$_2$ **7a’**), 1.09 (d, J_{HH} = 6.5 Hz, 12H, AlCH$_2$CH(C*H*$_3$)$_2$ **7a**), 1.14 (d, J_{HH} = 6.4 Hz, 6H, AlCH$_2$CH(C*H*$_3$)$_2$ **7a’**), 1.42-1.44 (m, 12H, and 12H, Ar-CH(C*H*$_3$)$_2$ **7a/7a’**), 1.78-1.94 (m, 4H and 4H, Ar-C*H*(CH$_3$)$_2$ **7a/7a’**), 6.43 (s, 2H, NC*H*C*H*N **7a**), 6.45 (s, 2H, NC*H*C*H*N **7a’**), 7.10-7.14 (m, 4H, and 4H, Ar-*o-H* **7a/7a’**), 7.23-7.28 (m, 2H, and 2H, Ar-*p-H* **7a/7a’**).

^{13}C{^{1}H} NMR (benzene-d_6): δ = 21.7 (Al*C*H$_2$CH(CH$_3$)$_2$), 22.9 (Ar-CH(*C*H$_3$)$_2$ **7a**), 23.2 (Ar-CH(*C*H$_3$)$_2$ **7a’**), 25.4 (Ar-CH(*C*H$_3$)$_2$ **7a’**), 25.7 (Ar-CH(*C*H$_3$)$_2$ **7a**), 27.6 (AlCH$_2$*C*H(CH$_3$)$_2$ **7a**), 28.1 (AlCH$_2$*C*H(CH$_3$)$_2$ **7a**), 28.2 (AlCH$_2$CH(*C*H$_3$)$_2$ **7a**), 28.9 (Ar-*C*H(CH$_3$)$_2$ **7a’**), 29.0 (Ar-*C*H(CH$_3$)$_2$ **7a**), 29.2 (AlCH$_2$CH(*C*H$_3$)$_2$ **7a’**), 123.9 (Ar-*C*), 124.2 (N*C*H*C*HN), 124.3 (N*C*H*C*HN), 130.7 (Ar-*C*), 135.0 (Ar-*C*), 135.4 (Ar-*C*),

145.8 (Ar-*C*), 145.9 (Ar-*C*). No resonances attributable to the carbenic carbon in **7c** and **7c’** could be observed.

^{27}Al NMR (benzene-d_6): No signal observed.

2.6 IMesAlMe$_2$H 6b and IMesAlMeH$_2$ 6b’

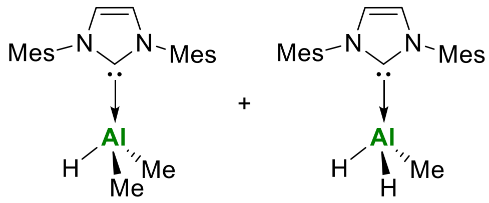

To a J. Young NMR tube was added IMesAlMe$_3$ (10 mg, 0.026 mmol) and IMesAlH$_3$ (8.9 mg, 0.026 mmol). Benzene-d_6 (0.5 mL) was then added and the tube sealed. The solution was subsequently heated to 100 ºC and the reaction was monitored by ^{1}H NMR spectroscopy. After 5 days of heating no further conversion was observed.

^{1}H NMR (benzene-d_6): δ = -0.81 (br d, 6H, AlC*H*$_3$ **6b**), -0.80 (br s, 6H, AlC*H*$_3$ **6b’**), 2.04-2.05 (m, 12H and 12H, Ar-*o*-CH$_3$ **6b/6b’**), 2.08-2.09 (m, 6H and 6H, Ar-*p*-CH$_3$ **6b/6b’**), 6.02 (s, 2H, NC*H*C*H*N **6b**), 6.05 (s, 2H, NC*H*C*H*N **6b’**), 6.74 (br s, 4H and 4H, Ar-*m-H* **6b/6b’**).

^{13}C{^{1}H} NMR (benzene-d_6): δ = 17.6 (br, Ar-*o*-*C*H$_3$ **6b/6b’**), 21.1 (br, Ar-*o*-*C*H$_3$ **6b/6b’**), 122.5 (N*C*H*C*HN **6b**), 122.7 (N*C*H*C*HN **6b**), 129.4 (br, Ar-*C* **6b/6b’**), 135.2 (Ar-*C*), 135.3 (Ar-*C*), 139.5 (Ar-*C*). No resonance attributable to the carbenic carbon or the Al-(*C*H$_3$)$_3$ groups of **6b** or **6b’** could be observed.

^{27}Al NMR (benzene-d_6): No signal observed.

2.7 IMesAl*i*Bu$_2$H 7b and IMesAl*i*BuH$_2$ 7b'

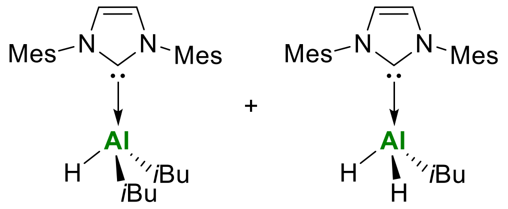

To a J. Young NMR tube was added IMesAl*i*Bu$_3$ (12 mg, 0.024 mmol) and IMesAlH$_3$ (8 mg, 0.024 mmol). Benzene-*d*$_6$ (0.5 mL) was then added and the tube sealed. The solution was subsequently heated to 100 ºC and the reaction was monitored by ^{1}H NMR spectroscopy. After 5 days of heating no further conversion was observed.

^{1}H NMR (benzene-*d*$_6$): δ = -0.35 - -0.30 (m, 2H, AlC*H*$_2$CH(CH$_3$)$_2$ **7b**), -0.20 - -0.14 (m, 2H and 2H, AlC*H*$_2$CH(CH$_3$)$_2$ **7b/7b'**), 1.13-1.21 (m, 6H (**7b'**) and 12H (**7b**), AlCH$_2$CH(C*H*$_3$)$_2$), 1.86-1.92 (m, 2H (**7b**) and 1H (**7b'**), AlCH$_2$C*H*(CH$_3$)$_2$), 2.04 (s, 12H, Ar-*o*-C*H*$_3$ **7b**), 2.05 (s, 12H, Ar-*o*-C*H*$_3$ **7b'**), 2.09 (s, 6H, Ar-*p*-C*H*$_3$ **7b'**), 2.09 (s, 6H, Ar-*p*-C*H*$_3$ **7b**), 5.99 (s, 2H, NC*H*C*H*N **7b**), 6.03 (s, 2H, NC*H*C*H*N **7b'**), 6.75 (br s, 4H, Ar-*m*-*H* **7b'**), 6.76 (br s, 4H, Ar-*m*-*H* **7b**).

^{13}C{^{1}H} NMR (benzene-*d*$_6$): δ = 17.8 (Ar-*o*-*C*H$_3$), 17.9 (Ar-*o*-*C*H$_3$), 21.0 (Ar-*p*-*C*H$_3$), 21.1 (Ar-*p*-*C*H$_3$), 21.6 (Al*C*H$_2$CH(CH$_3$)$_2$), 28.0 (AlCH$_2$*C*H(CH$_3$)$_2$), 28.2 (AlCH$_2$*C*H(CH$_3$)$_2$), 28.6 (AlCH$_2$CH(*C*H$_3$)$_2$), 28.9 (AlCH$_2$CH(*C*H$_3$)$_2$), 122.5 (N*C*H*C*HN), 122.7 (N*C*H*C*HN), 129.4 (Ar-*C*), 135.1 (Ar-*C*), 135.3 (Ar-*C*), 135.4 (Ar-*C*), 139.5 (Ar-*C*). No resonance attributable to the carbenic carbon of **7b** or **7b'** could be observed.

^{27}Al NMR (benzene-*d*$_6$): No signal observed.

2.8 ICyAlMe$_2$H 6c and ICyAlMeH$_2$ 6c'

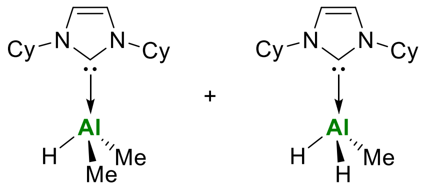

To a J. Young NMR tube was added ICyAlMe$_3$ (10 mg, 0.033 mmol) and ICyAlH$_3$ (8.6 mg, 0.033 mmol). Benzene-*d*$_6$ (0.5 mL) was then added and the tube sealed. The solution

was subsequently heated to 100 ºC and the reaction was monitored by ^{1}H NMR spectroscopy. After 3 days of heating no further conversion was observed.

^{1}H NMR (benzene-d_6): δ = -0.08 (br d, 6H, AlCH_3 **6c**), -0.05 (br t, 3H, AlCH_3 **6c'**), 0.78-0.90 (m, 2H and 2H, Cy-H_2 **6c/6c'**), 1.03-1.14 (m, 4H and 4H, Cy-H_2 **6c/6c'**), 1.21-1.28 (m, 4H and 4H, Cy-H_2 **6c/6c'**), 1.37-1.42 (m, 2H and 2H, Cy-H_2 **6c/6c'**), 1.47-1.52 (m, 4H and 4H, Cy-H_2 **6c/6c'**), 1.81-1.90 (m, 4H and 4H, Cy-H_2 **6c/6c'**), 4.94-5.04 (m, 2H and 2H, Cy-*H* **6c/6c'**), 6.35-6.36 (m, 2H and 2H, NC*H*C*H*N **6c/6c'**).

^{13}C{^{1}H} NMR (benzene-d_6): δ = 25.3 (Cy-CH$_2$), 25.4 (Cy-CH$_2$), 25.5 (Cy-CH$_2$), 34.2 (Cy-CH$_2$), 34.4 (Cy-CH$_2$), 34.5 (Cy-CH$_2$), 58.3 (Cy-*C*H), 58.6 (Cy-*C*H), 116.9 (NCH*C*HN), 117.1 (N*C*HCHN). No resonance attributable to the carbenic carbon or the Al-(CH$_3$)$_3$ groups of **6c** or **6c'** could be observed.

^{27}Al NMR (benzene-d_6): No signal observed.

2.9 ICyAl*i*Bu$_2$H 7c and ICyAl*i*BuH$_2$ 7c'

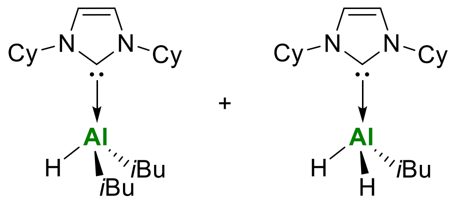

To a J. Young NMR tube was added ICyAl*i*Bu$_3$ (10 mg, 0.023 mmol) and ICyAlH$_3$ (6 mg, 0.023 mmol). Benzene-d_6 (0.5 mL) was then added and the tube sealed. The solution was subsequently heated to 100 ºC and the reaction was monitored by ^{1}H NMR spectroscopy. After 3 days of heating no further conversion was observed.

^{1}H NMR (benzene-d_6): δ = 0.42-0.47 (m, 2H and 2H, AlCH_2CH(CH$_3$)$_2$ **7c/7c'**), 0.53-0.58 (m, 2H, AlCH_2CH(CH$_3$)$_2$ **7c**), 0.62 (br t, 2H, AlCH_2CH(CH$_3$)$_2$ **7c'**), 0.82-0.92 (m, 2H, Cy-H_2), 1.06-1.16 (m, 4H and 4H, Cy-H_2 **7c/7c'**), 1.33 (d, J_{HH} = 6.5 Hz, 6H (**7c)** and 3H (**7c'**), AlCH$_2$CH(CH_3)$_2$), 1.40 (d, J_{HH} = 6.5 Hz, 6H (**7c)** and 3H (**7c'**), AlCH$_2$CH(CH_3)$_2$), 1.52-1.57 (m, 4H and 4H, Cy-H_2 **7c/7c'**), 1.90-1.94

(m, 4H and 4H, Cy-*H*$_2$ **7c**/**7c'**), 2.21-2.31 (m, 2H (**7c**) and 1H (**7c'**), AlCH$_2$C*H*(CH$_3$)$_2$), 4.97-5.04 (m, 2H and 2H, Cy-*H* **7c**/**7c'**), 6.33 (s, 2H and 2H, NC*H*C*H*N **7c**/**7c'**). Two Cy resonances masked by *i*Bu CH$_3$ resonances.

^{13}C{^{1}H} NMR (benzene-*d*$_6$): δ = 23.7 (Al*C*H$_2$CH(CH$_3$)$_2$), 25.4 (Cy-*C*H$_2$), 25.5 (Cy-*C*H$_2$), 25.6 (Cy-*C*H$_2$), 28.6 (Cy-*C*H$_2$), 29.0 (Cy-*C*H$_2$), 34.3 (Cy-*C*H$_2$), 34.5 (Cy-*C*H$_2$), 58.4 (Cy-*C*H), 117.2 (N*C*H*C*HN). No resonance attributable to the carbenic carbon of **7c** or **7c'** could be observed.

^{27}Al NMR (benzene-*d*$_6$): No signal observed.

3. NMR Spectroscopy

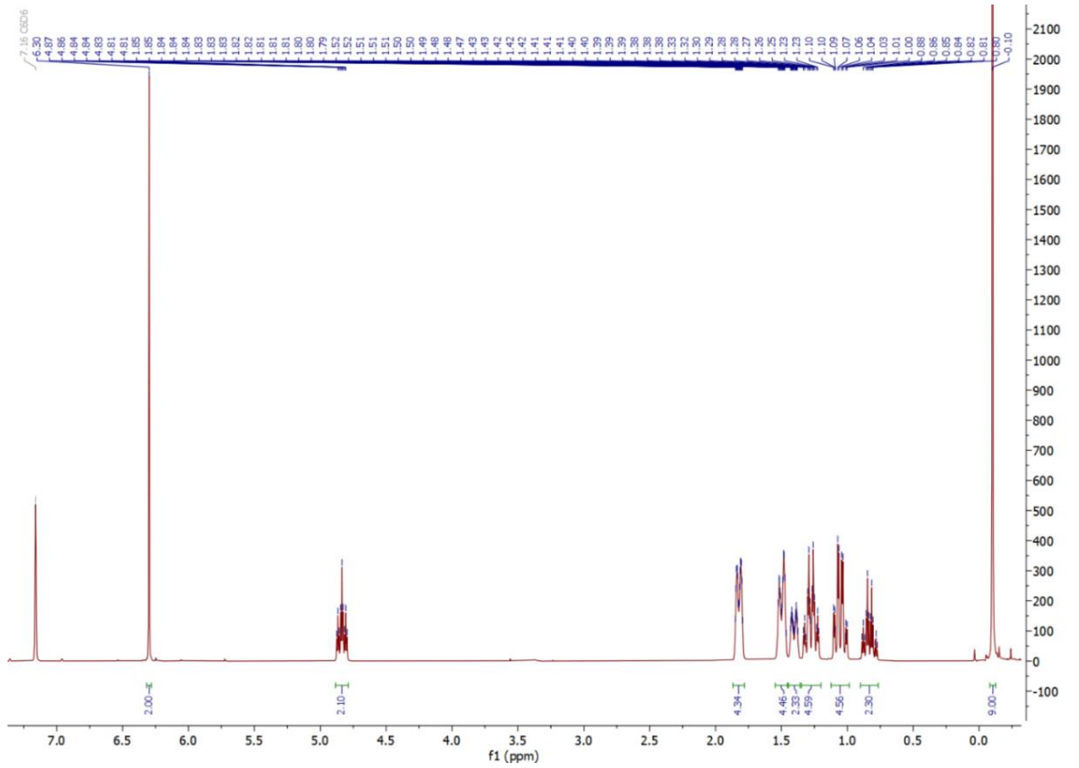

*Figure S1 - ^{1}H NMR spectrum of ICyAlMe$_3$ **4c**.*

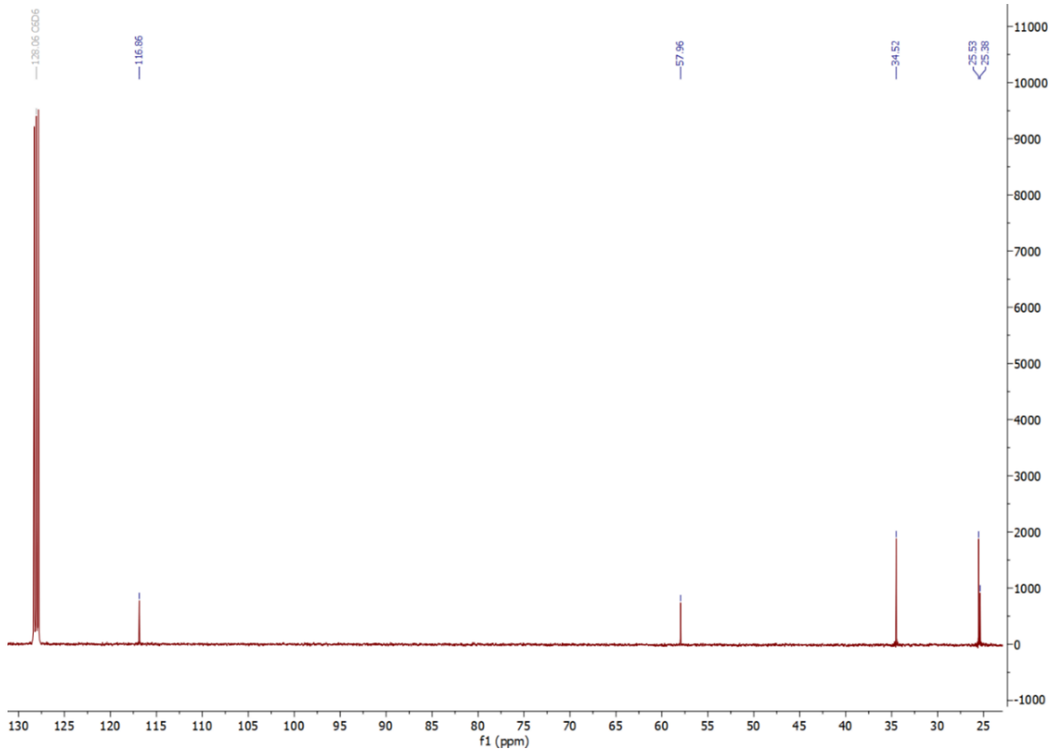

*Figure S2 - $^{13}C\{^{1}H\}$ NMR spectrum of **4c**.*

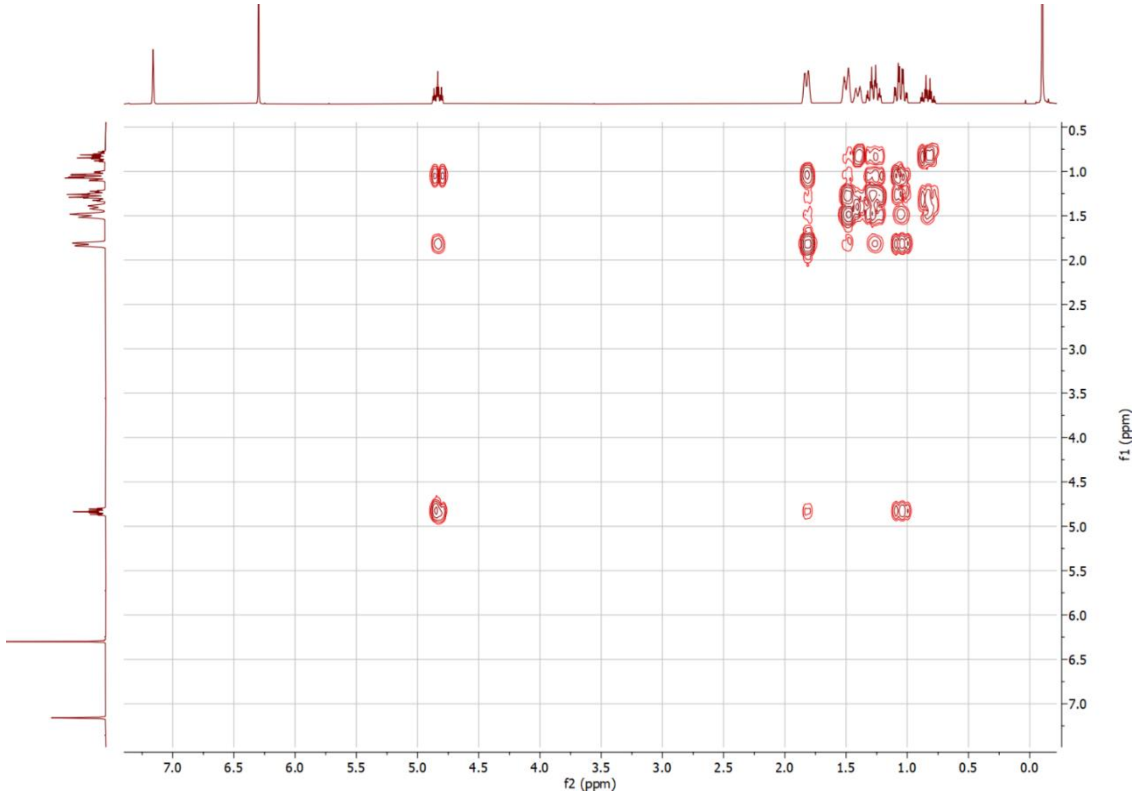

*Figure S3 - ^{1}H-^{1}H COSY NMR spectrum of **4c**.*

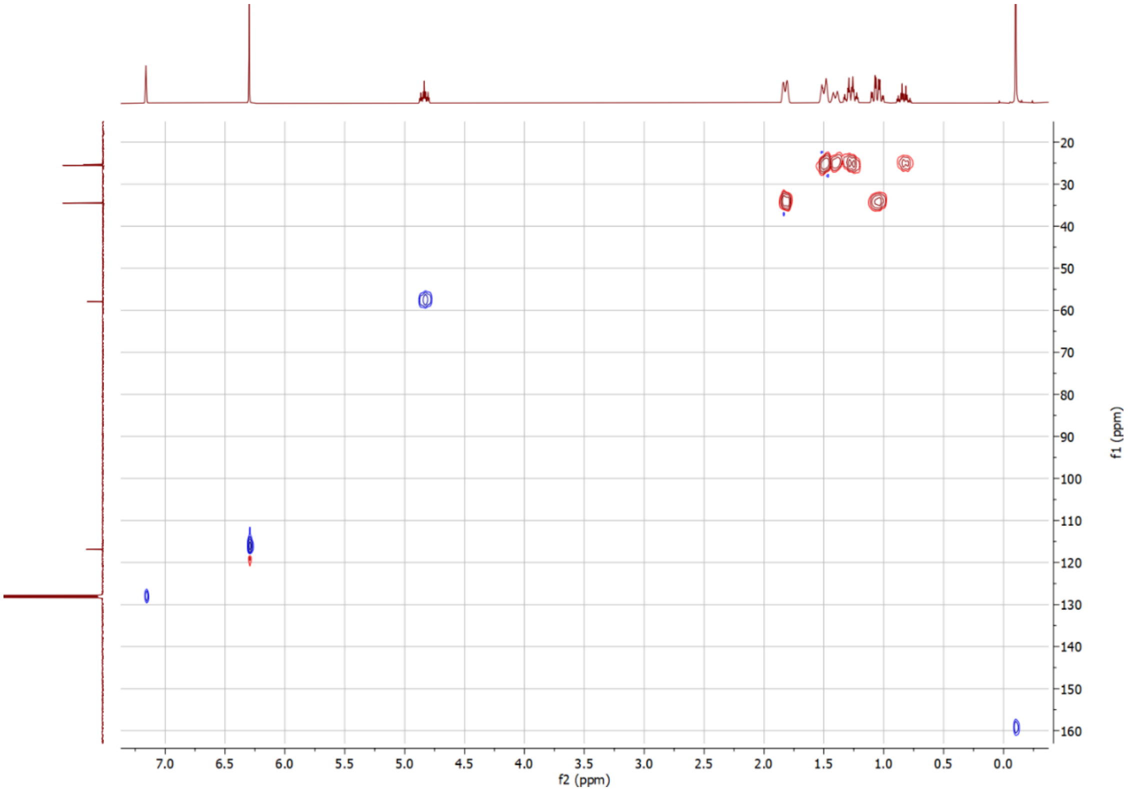

*Figure S4 - ^{1}H-^{13}C HSQC NMR spectrum of **4c**.*

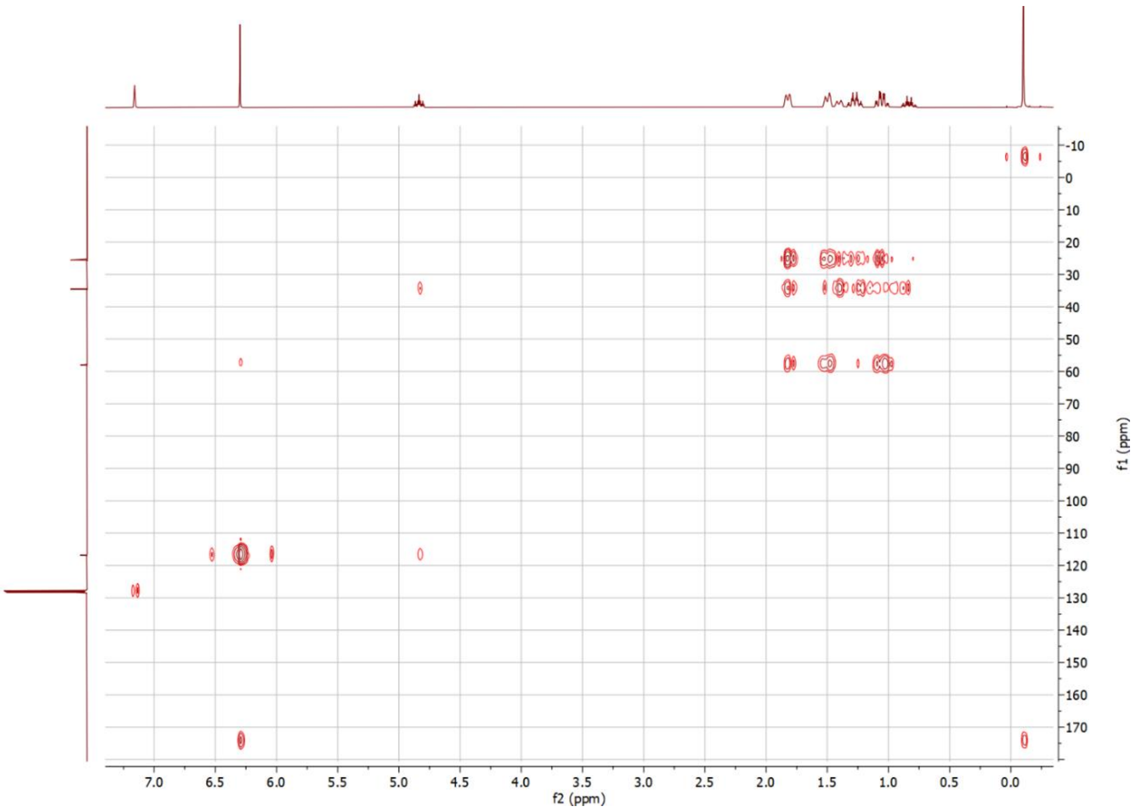

*Figure S5 - ^{1}H-^{13}C HMBC NMR spectrum of **4c**.*

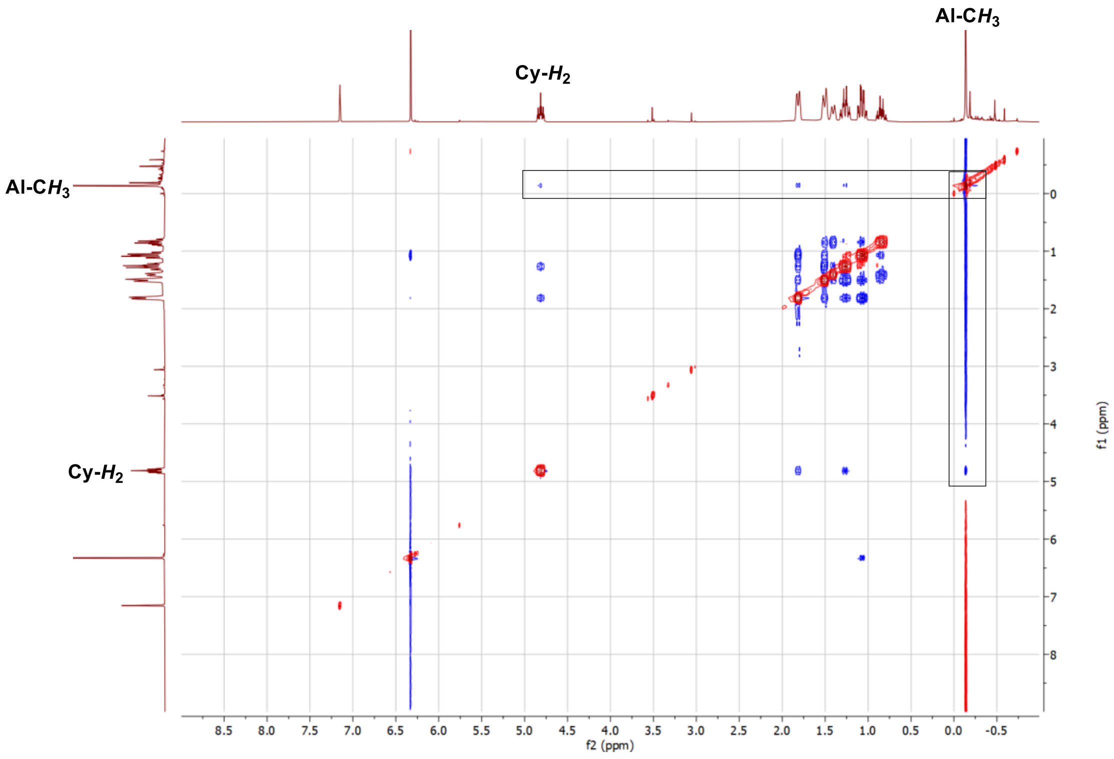

*Figure S6 – NOESY spectrum of **4c** showing interaction between Cy-H_2 and Al-CH_3 groups.*

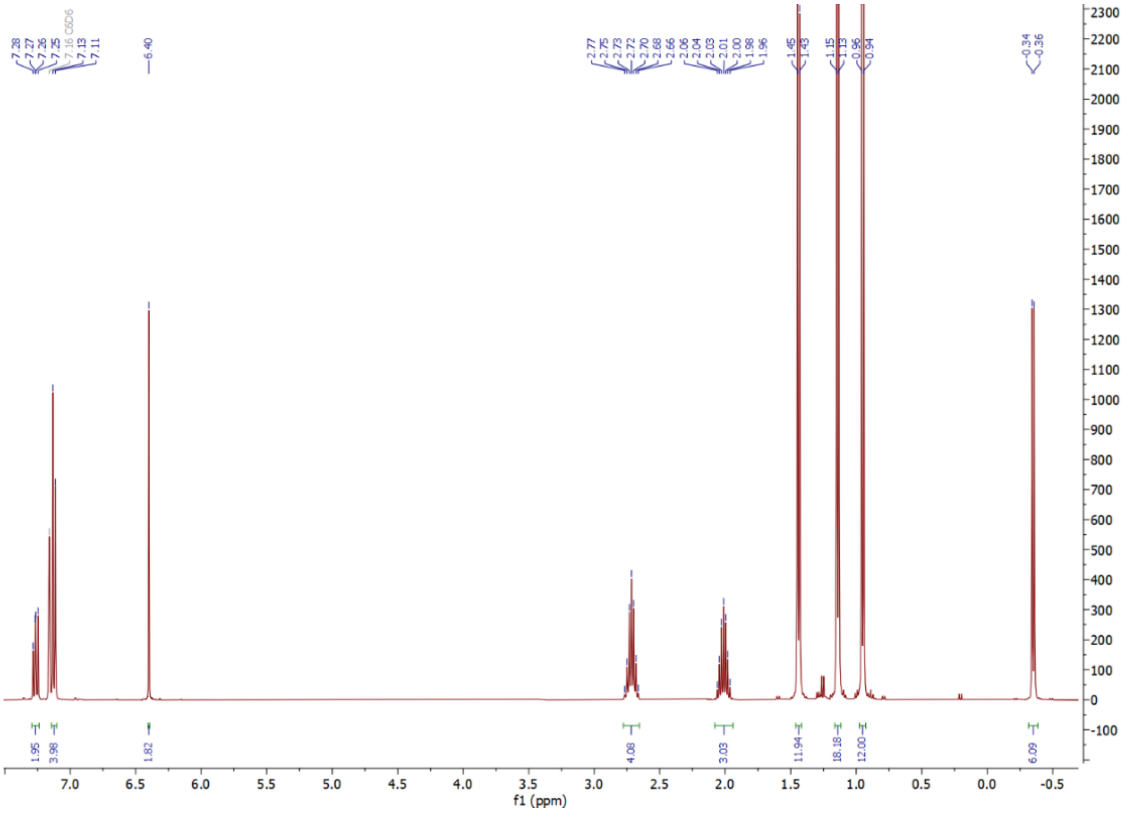

Figure S7 - ^{1}H NMR spectrum of IDipAliBu$_3$ ***5a****.*

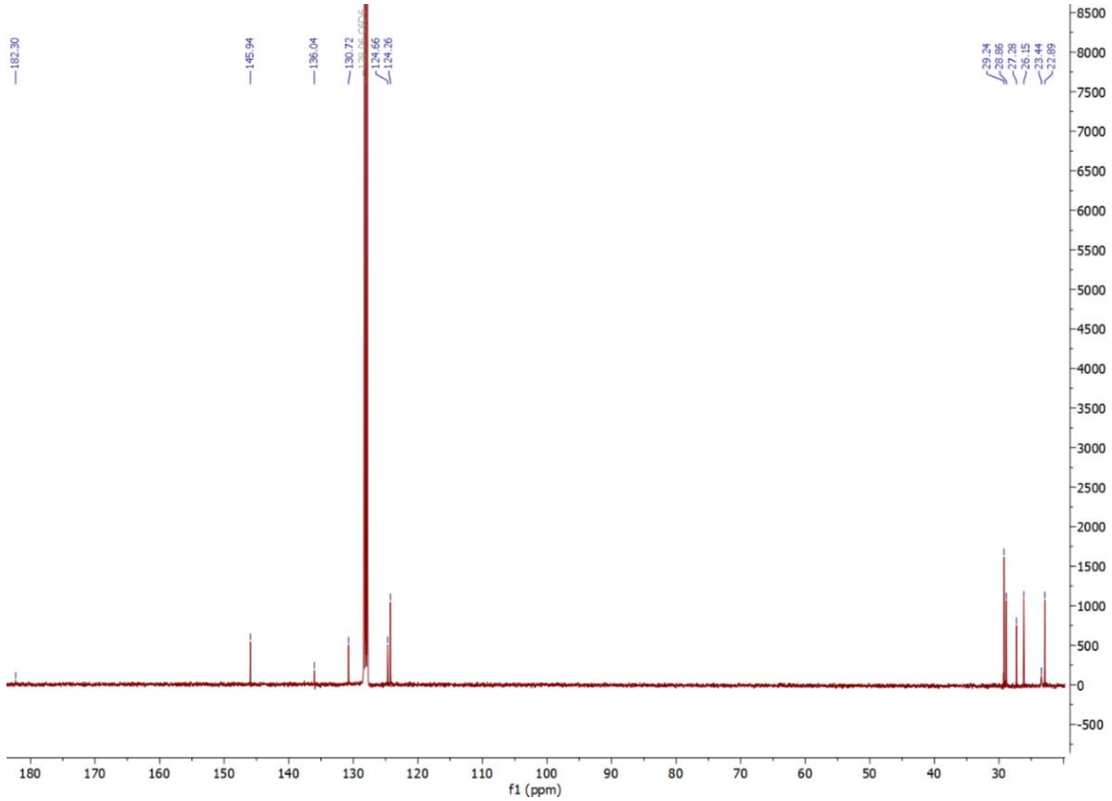

Figure S8 - ^{13}C{^{1}H} NMR spectrum of ***5a****.*

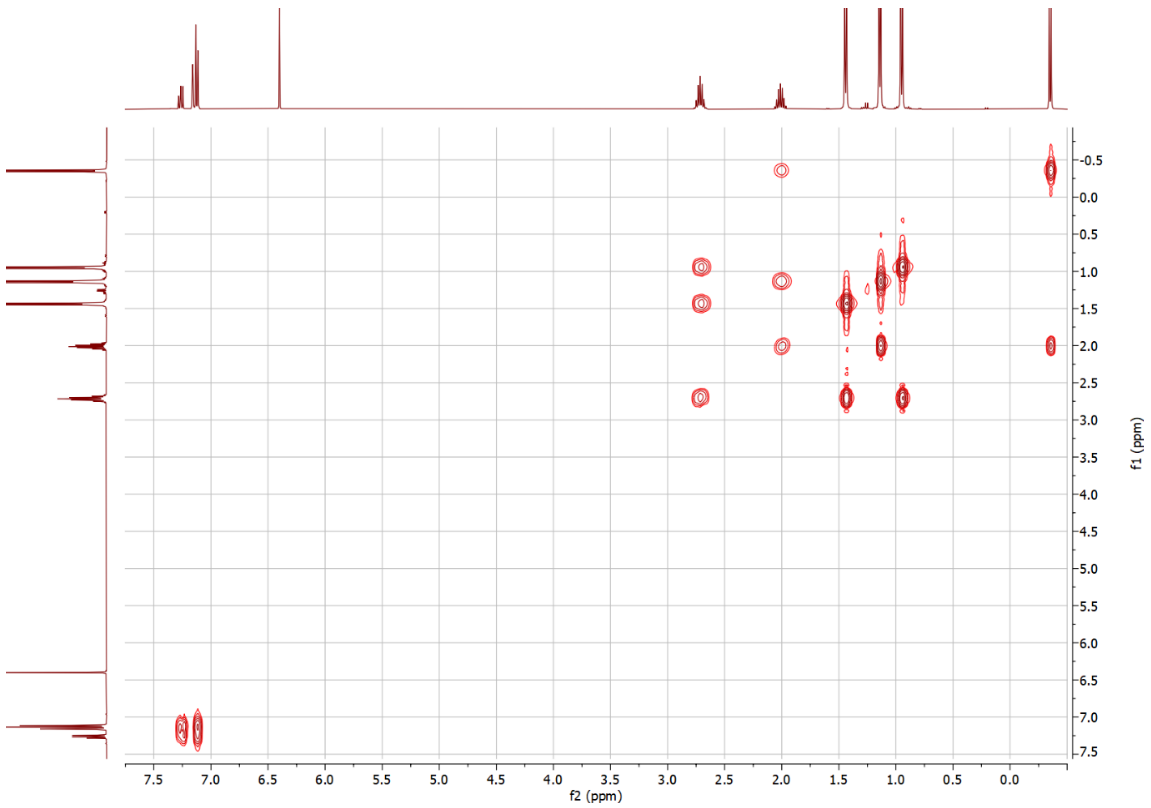

Figure S9 - ^{1}H-^{1}H COSY NMR spectrum of ***5a****.*

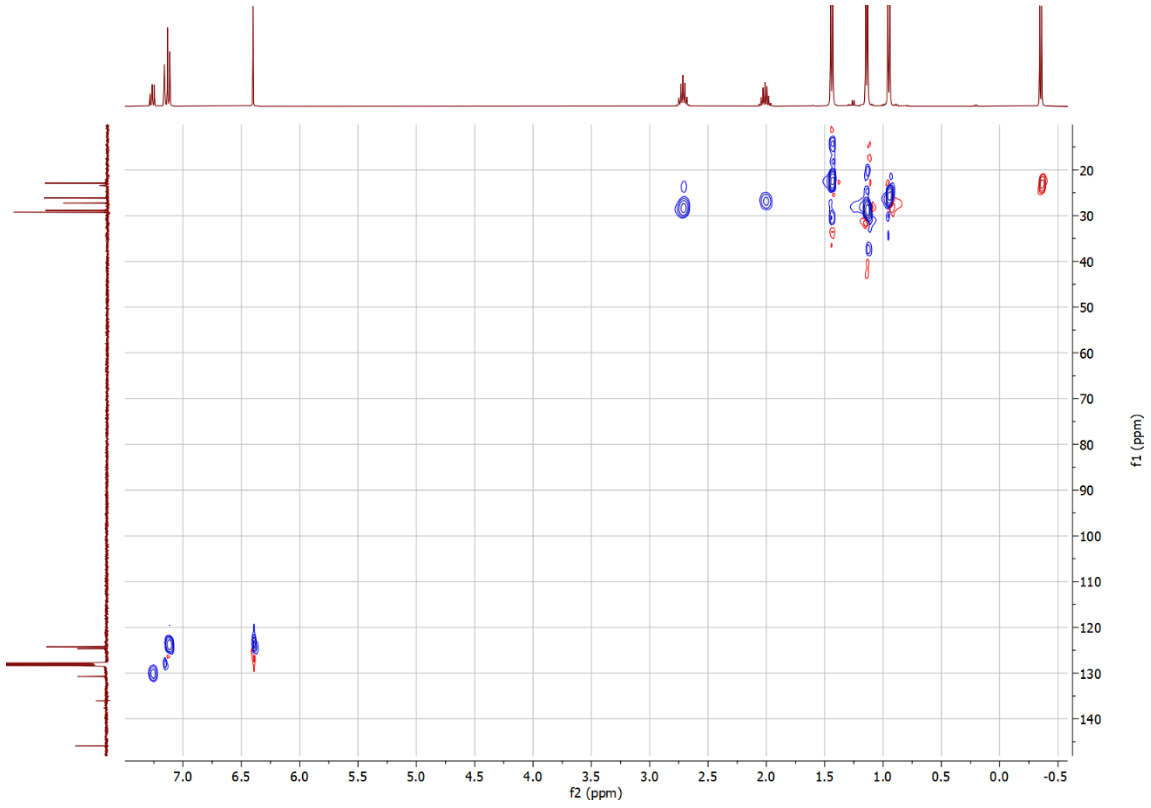

Figure S10 - ^{1}H-^{13}C HSQC NMR spectrum of ***5a****.*

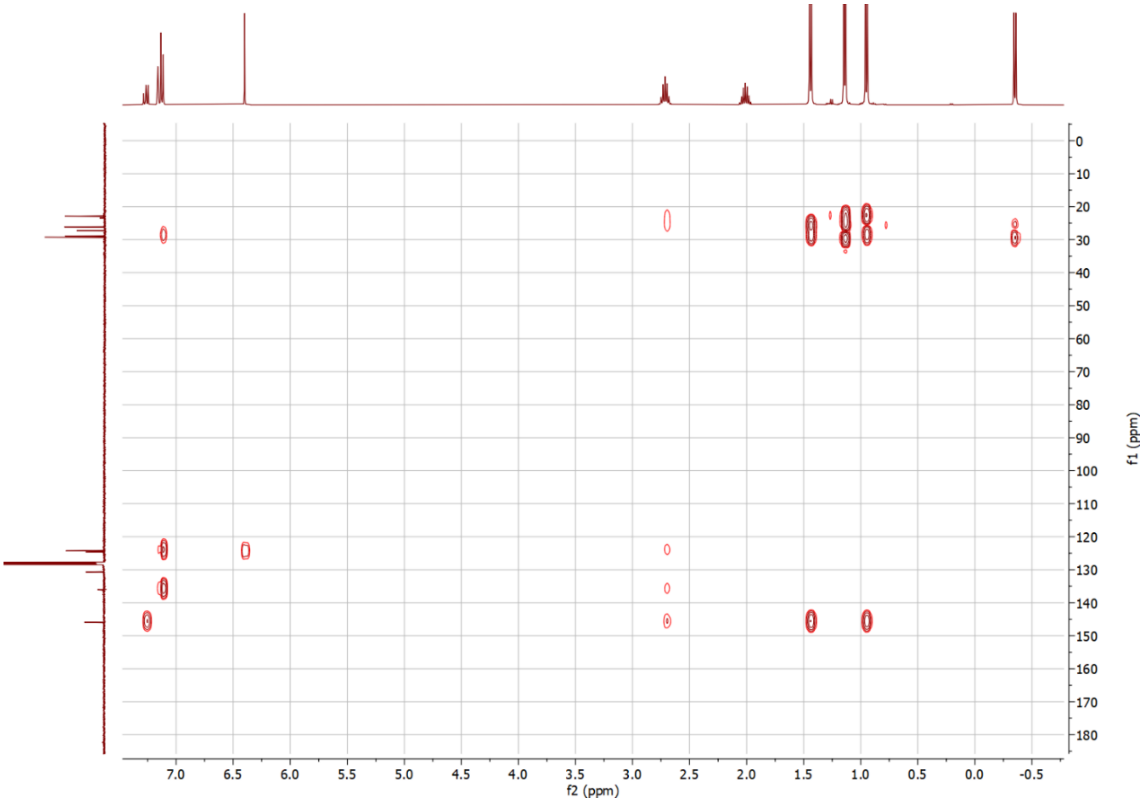

*Figure S11 - ^{1}H-^{13}C HMBC NMR spectrum of **5a**.*

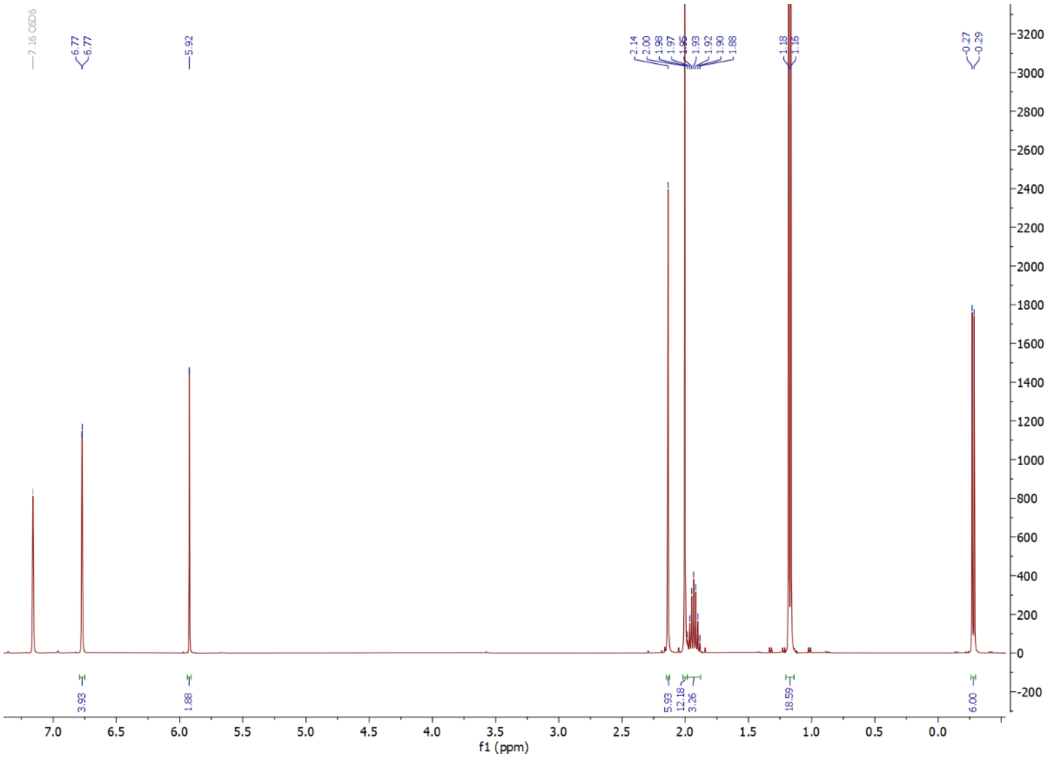

*Figure S12 - ^{1}H NMR spectrum of IMesAliBu$_3$ **5b**.*

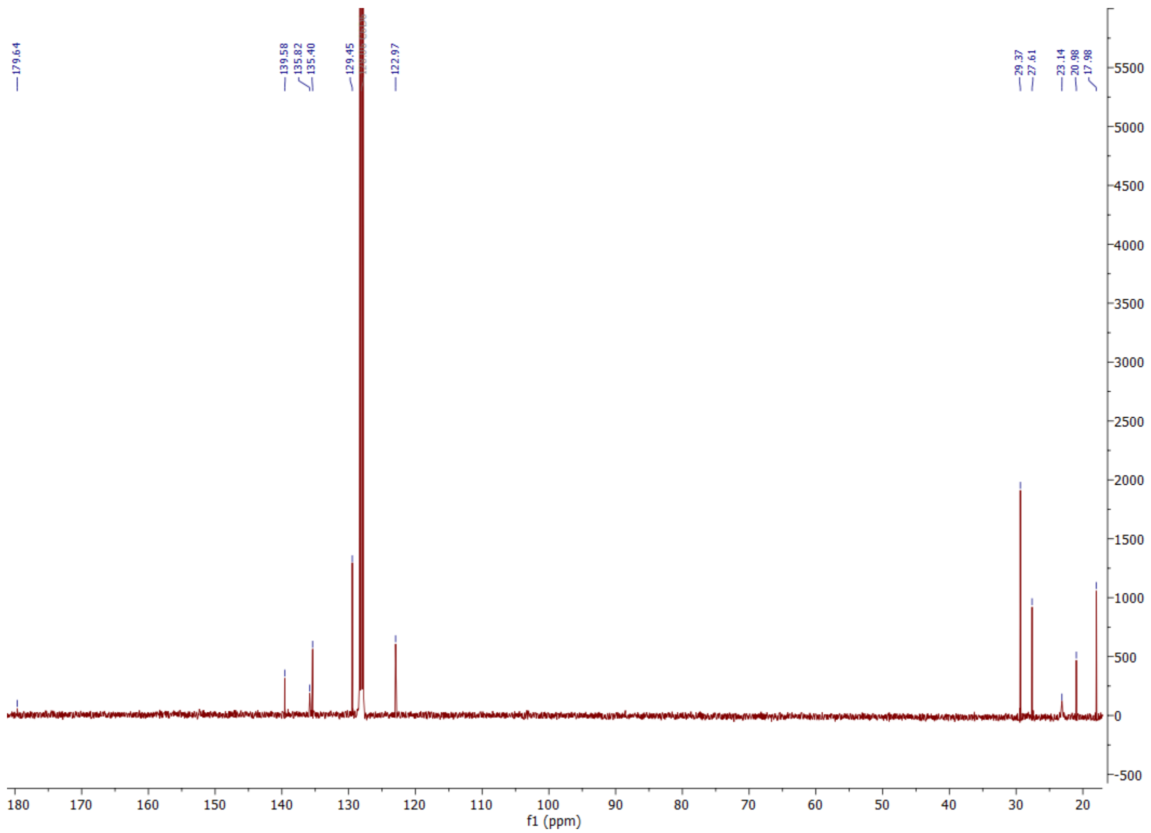

*Figure S13 - $^{13}C\{^1H\}$ NMR spectrum of **5b**.*

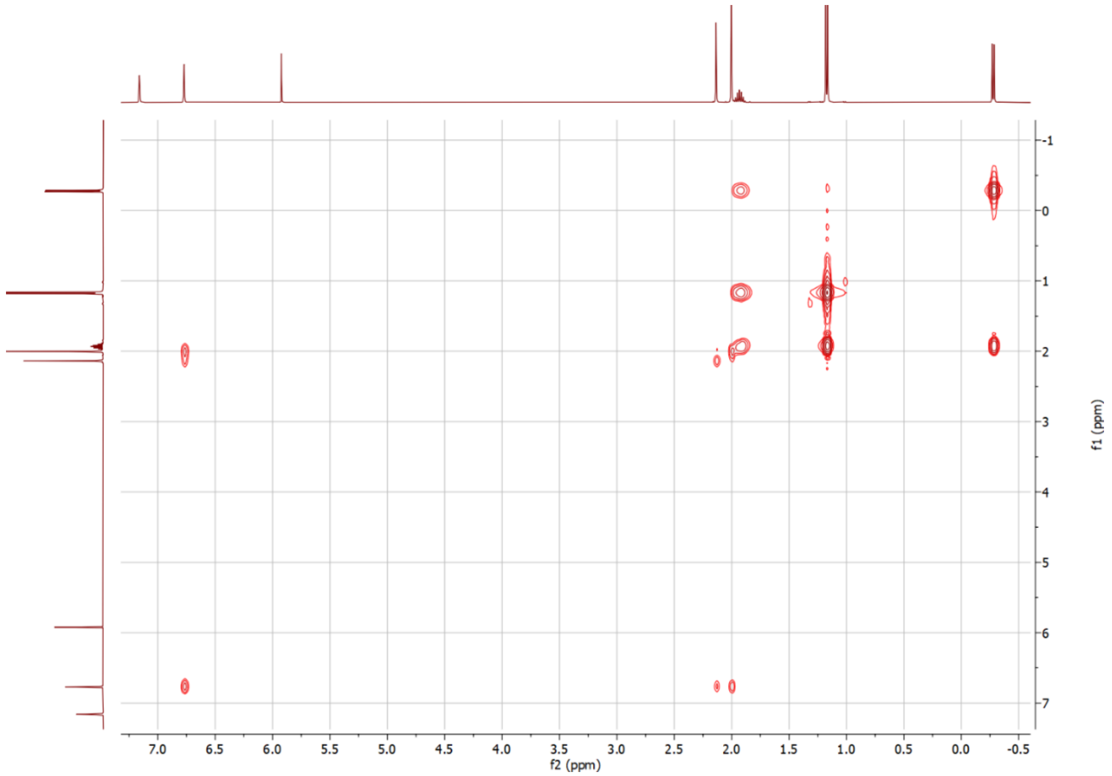

*Figure S14 - ^1H-^1H COSY NMR spectrum of **5b**.*

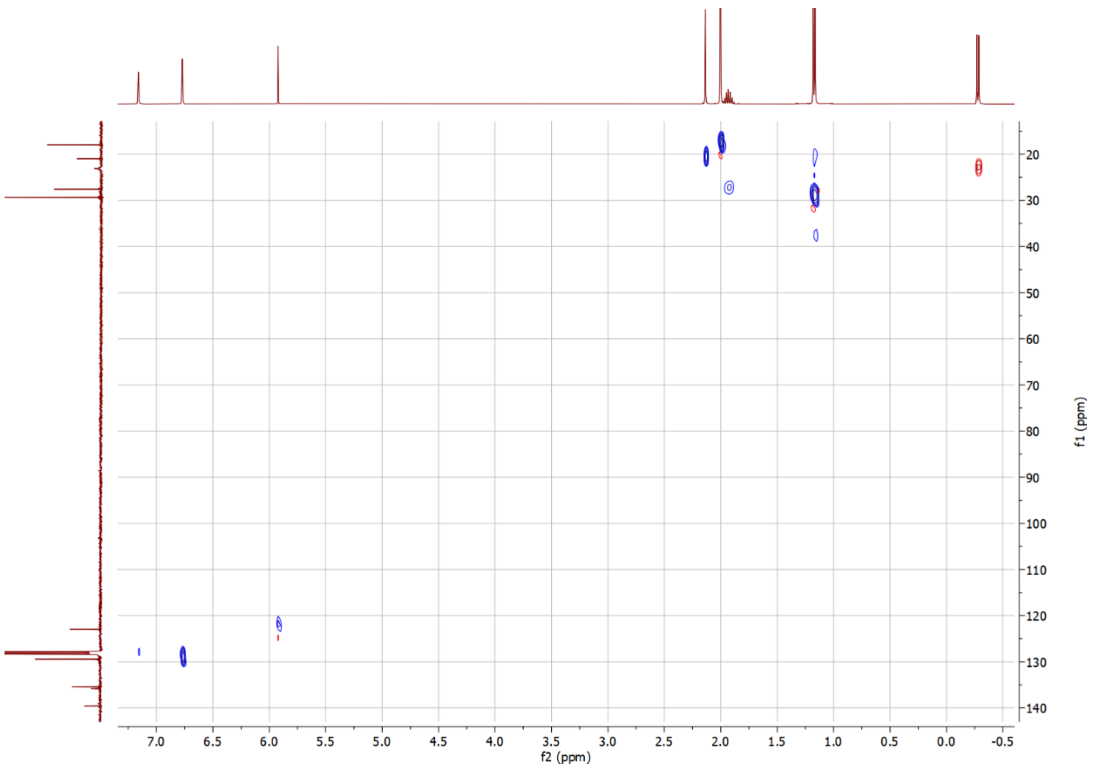

Figure S15 - ^{1}H-^{13}C HSQC NMR spectrum of ***5b****.*

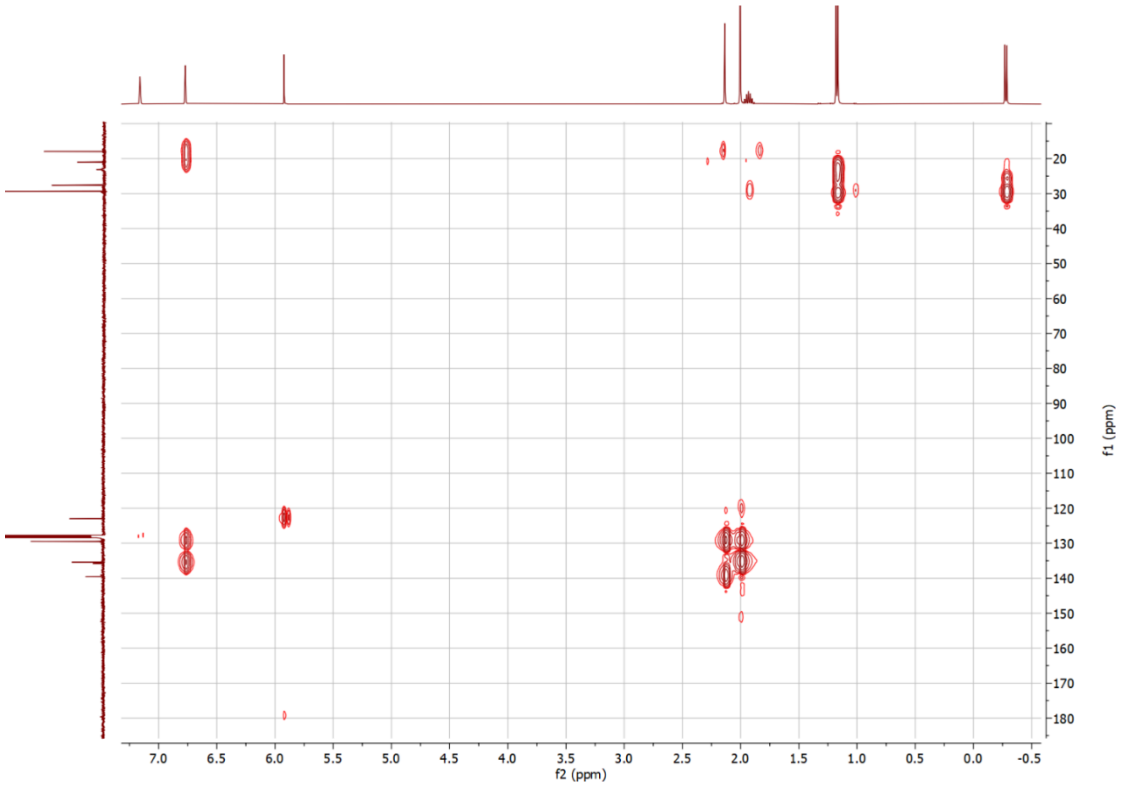

Figure S16 - ^{1}H-^{13}C HMBC NMR spectrum of ***5b****.*

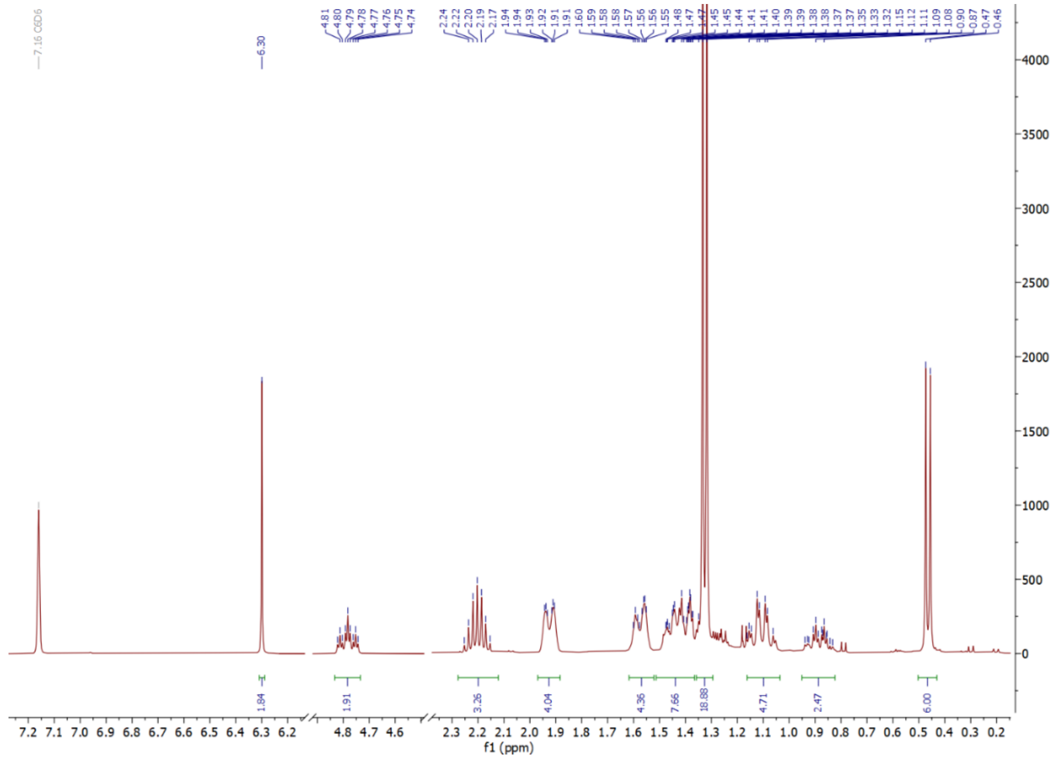

*Figure S17 - ^{1}H NMR spectrum of ICyAliBu$_3$ **5c**.*

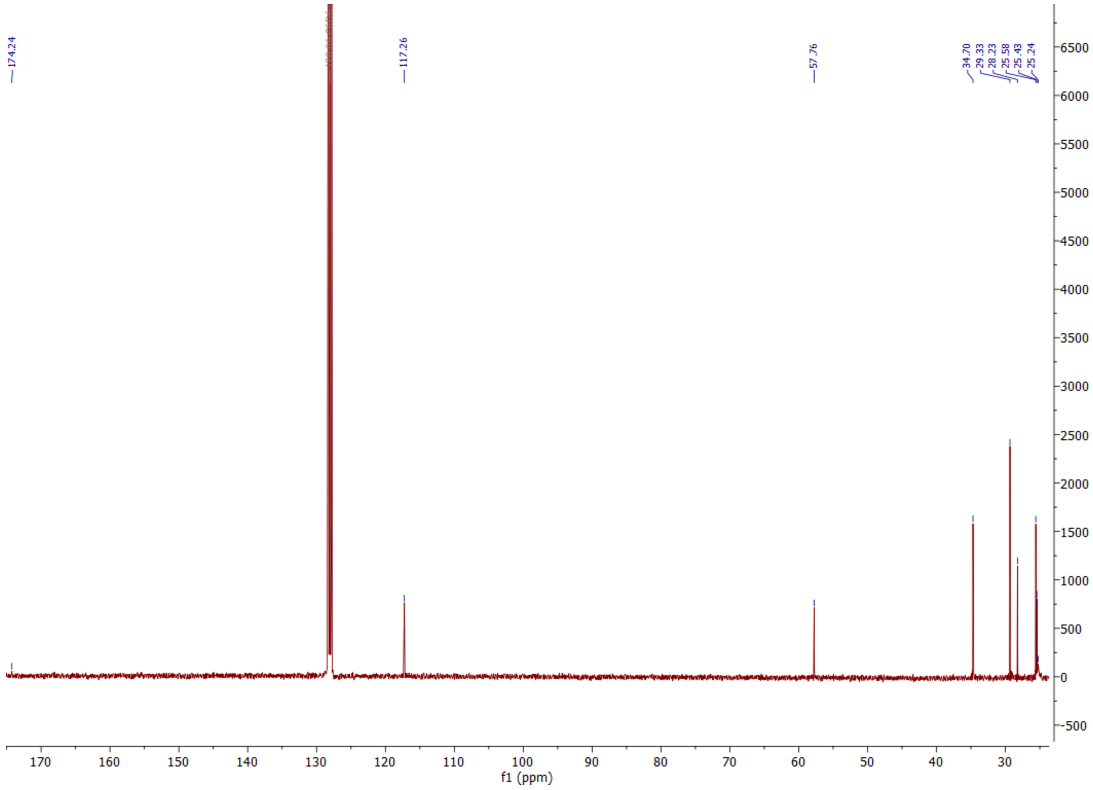

*Figure S18 - $^{13}C\{^{1}H\}$ NMR spectrum of **5c**.*

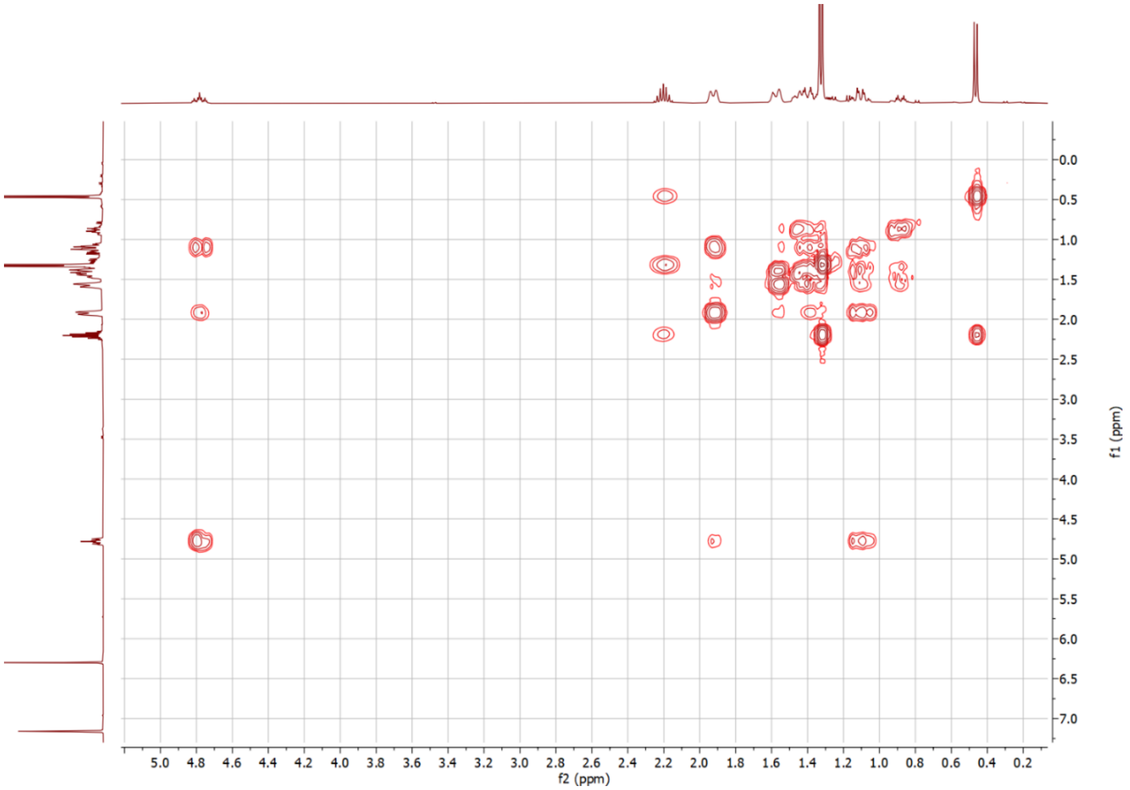

*Figure S19 - ^{1}H-^{1}H COSY NMR spectrum of **5c**.*

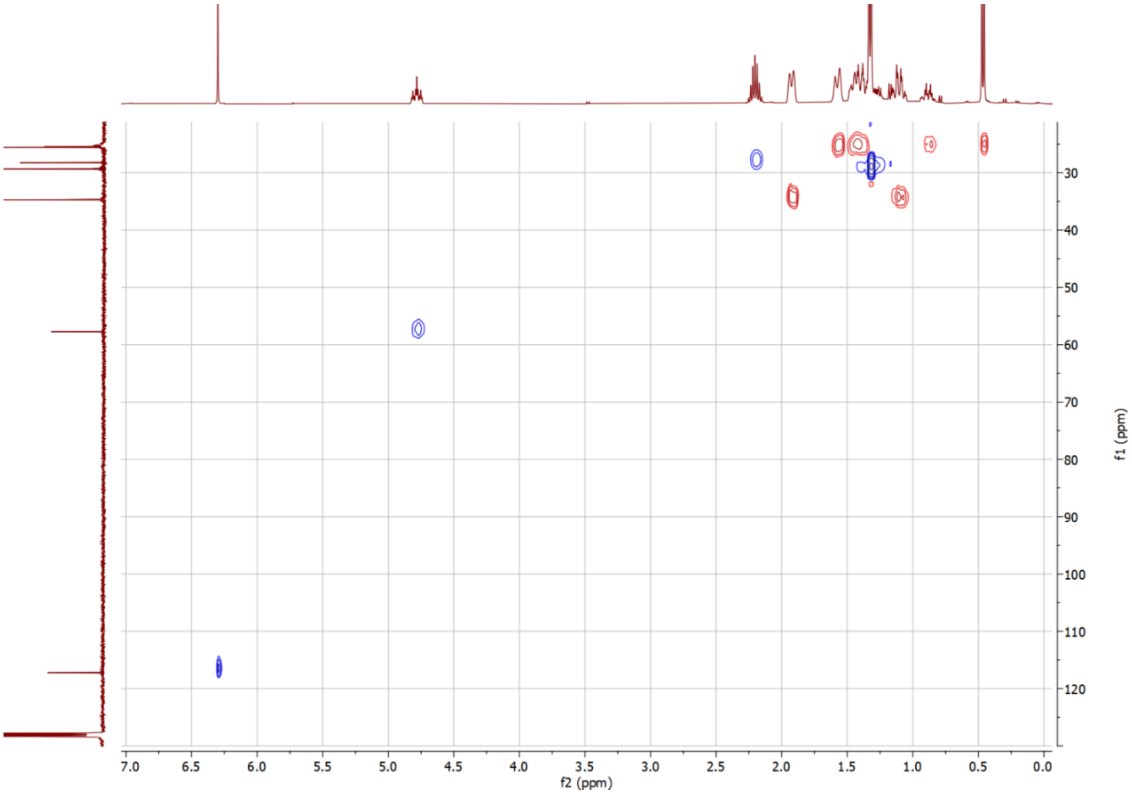

*Figure S20 - ^{1}H-^{13}C HSQC NMR spectrum of **5c**.*

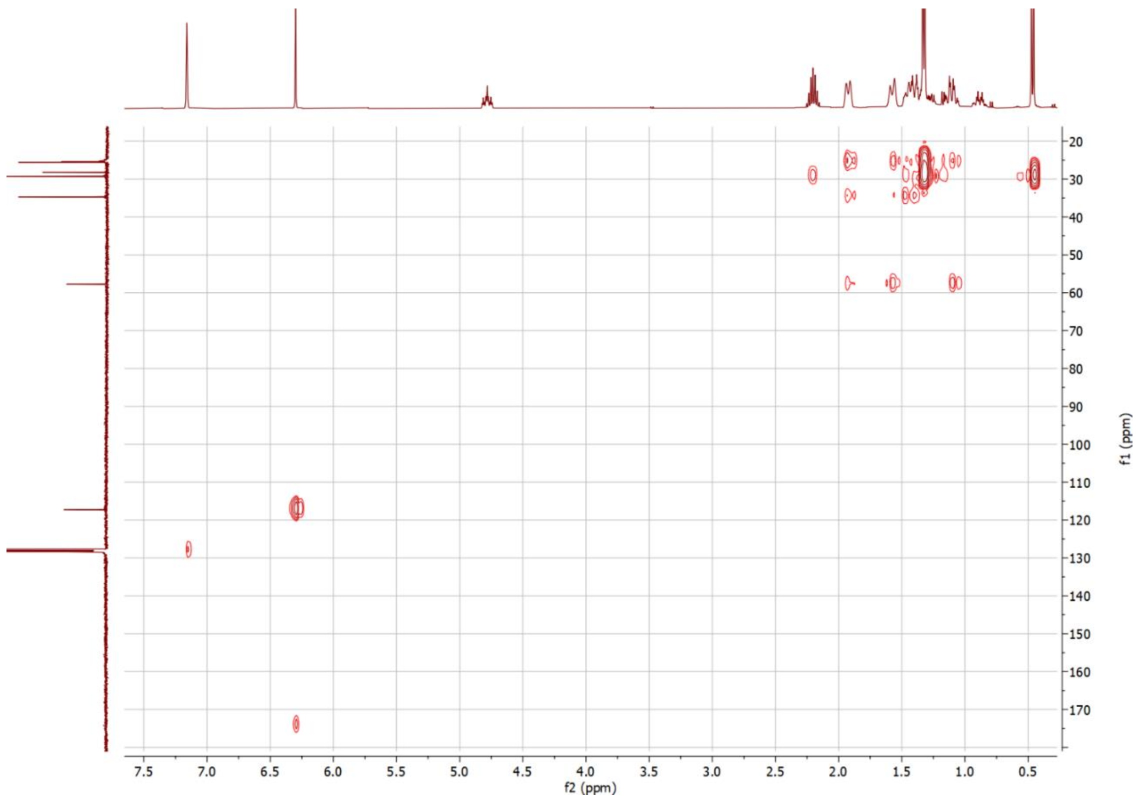

*Figure S21 - ^{1}H-^{13}C HMBC NMR spectrum of **5c**.*

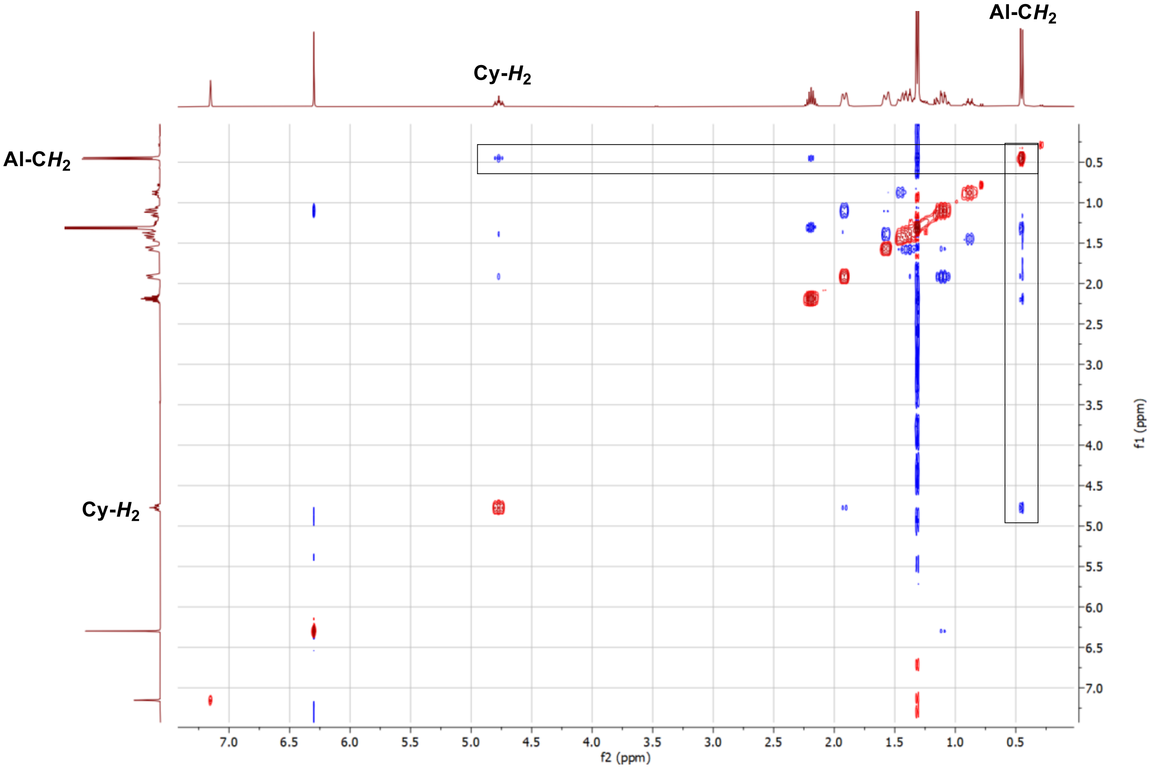

*Figure S22 - NOESY spectrum of **5c** showing interaction between Cy-H_2 and Al-CH_2 groups.*

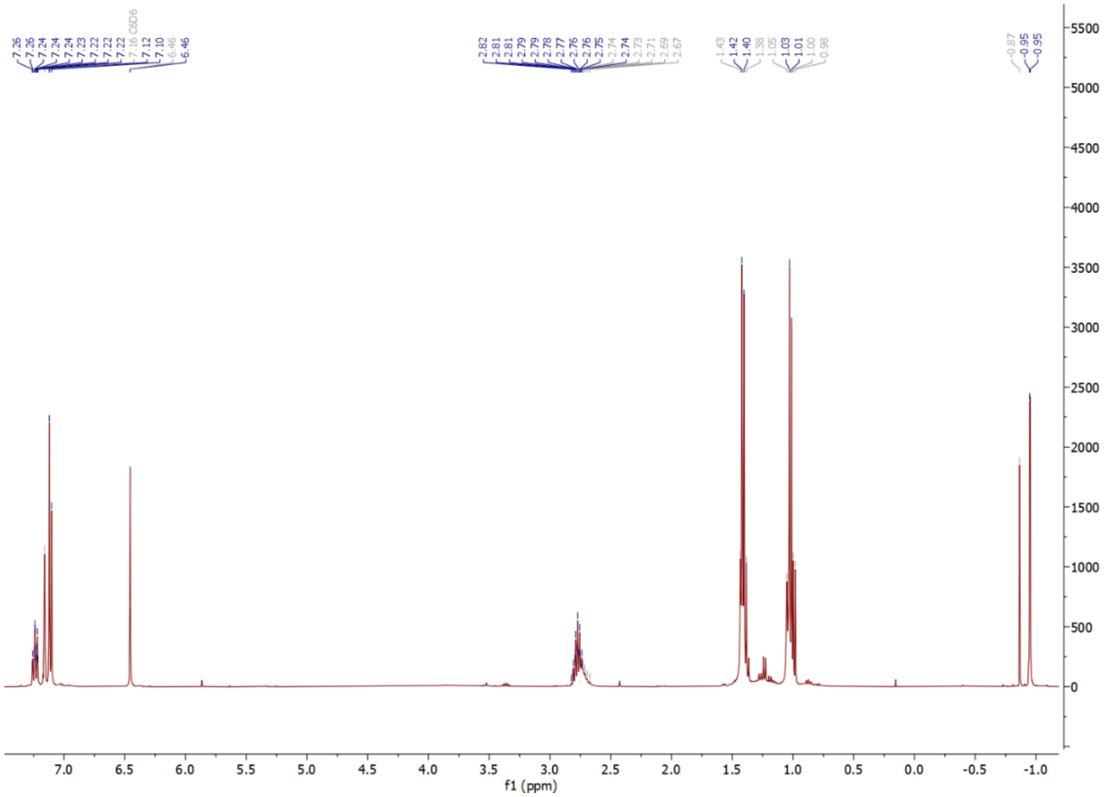

*Figure S23 - ^{1}H NMR spectrum of IDipAlMe$_2$H **6a** and IDipAlMeH$_2$ **6a'**. Starting material resonances labelled in grey. No integrals provided due to resonance overlap preventing accurate assignment.*

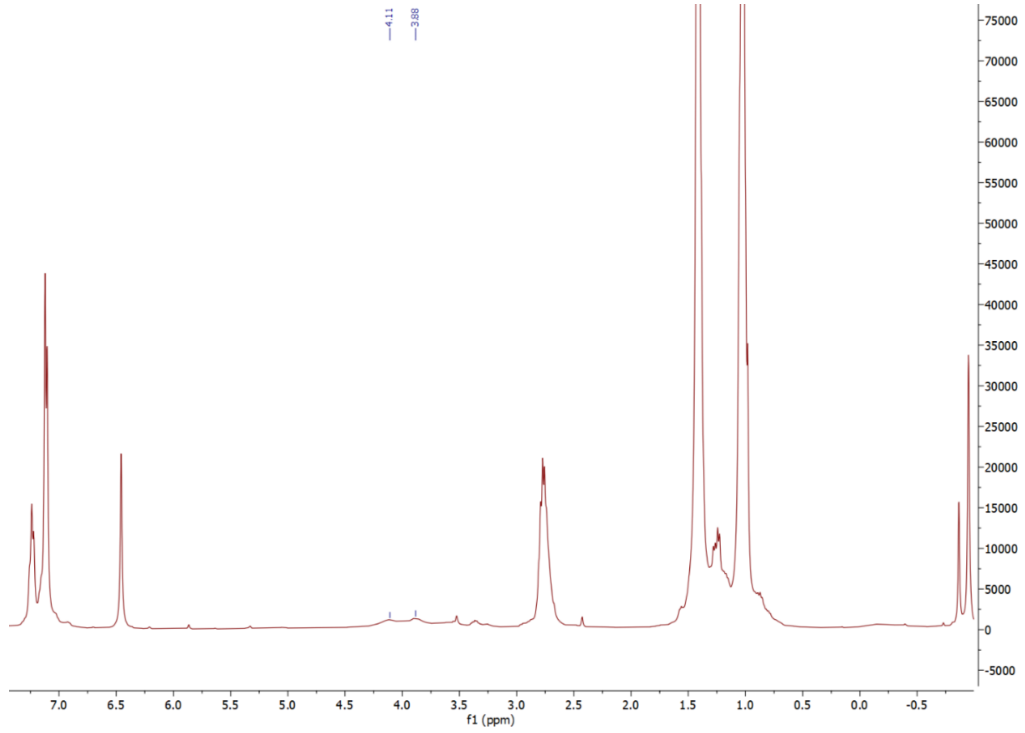

*Figure S24 - ^{1}H{^{27}Al} NMR spectrum of **6a** and **6a'**.*

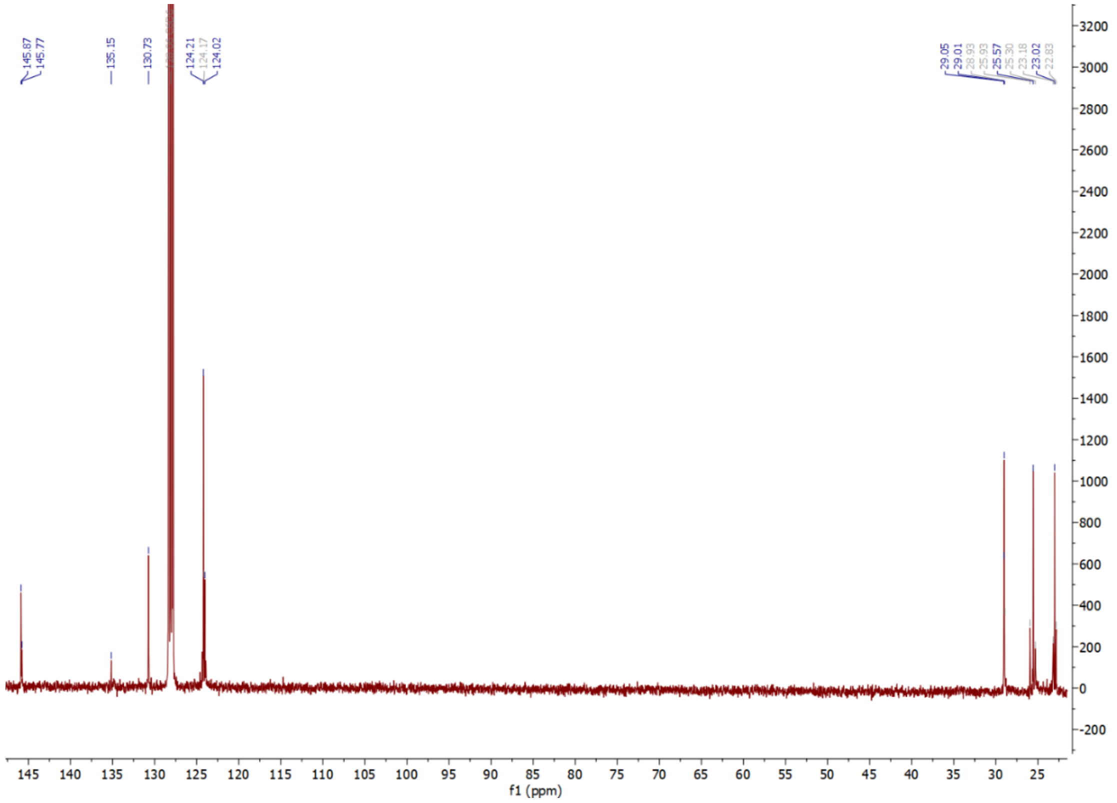

*Figure S25 - $^{13}C\{^{1}H\}$ NMR spectrum of **6a** and **6a'**. Starting material resonances labelled in grey.*

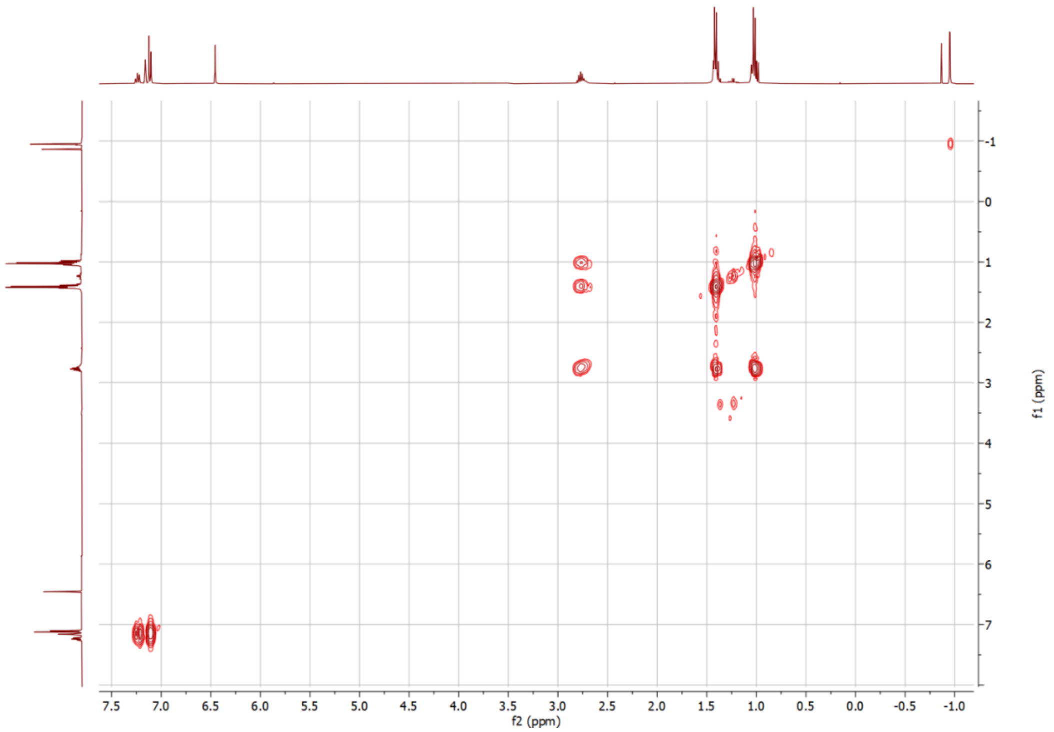

*Figure S26 - ^{1}H-^{1}H COSY NMR spectrum of **6a** and **6a'**.*

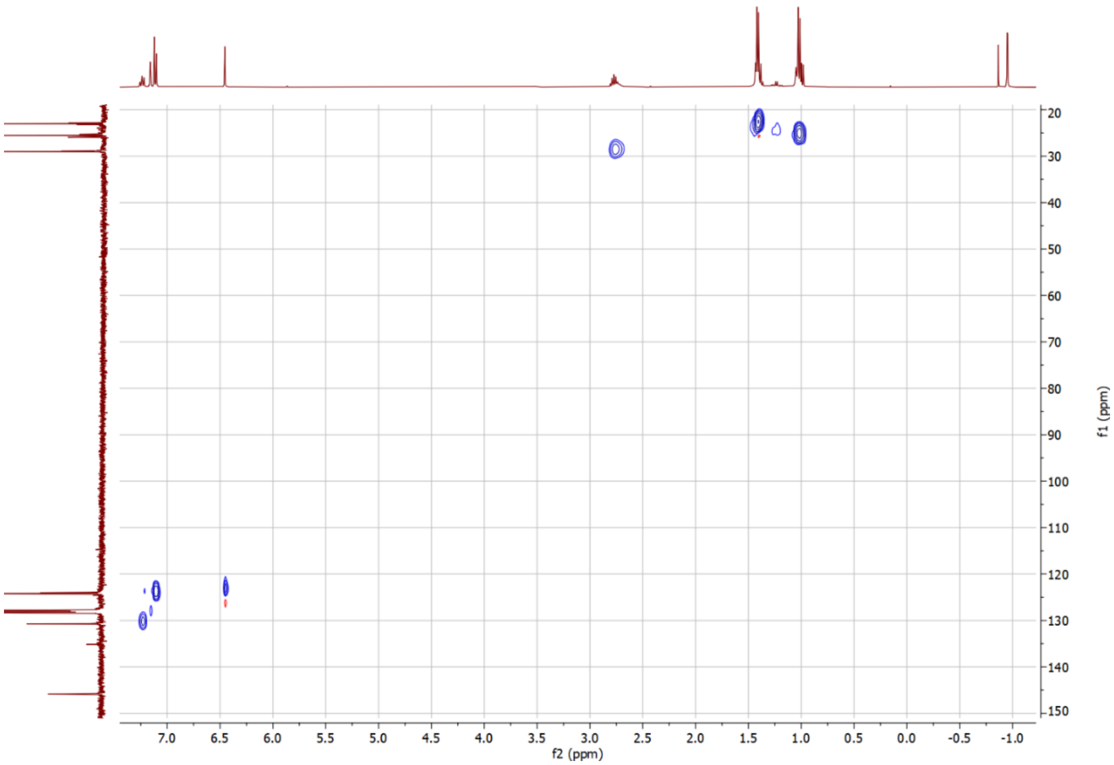

*Figure S27 - ^{1}H-^{13}C HSQC NMR spectrum of **6a** and **6a'**.*

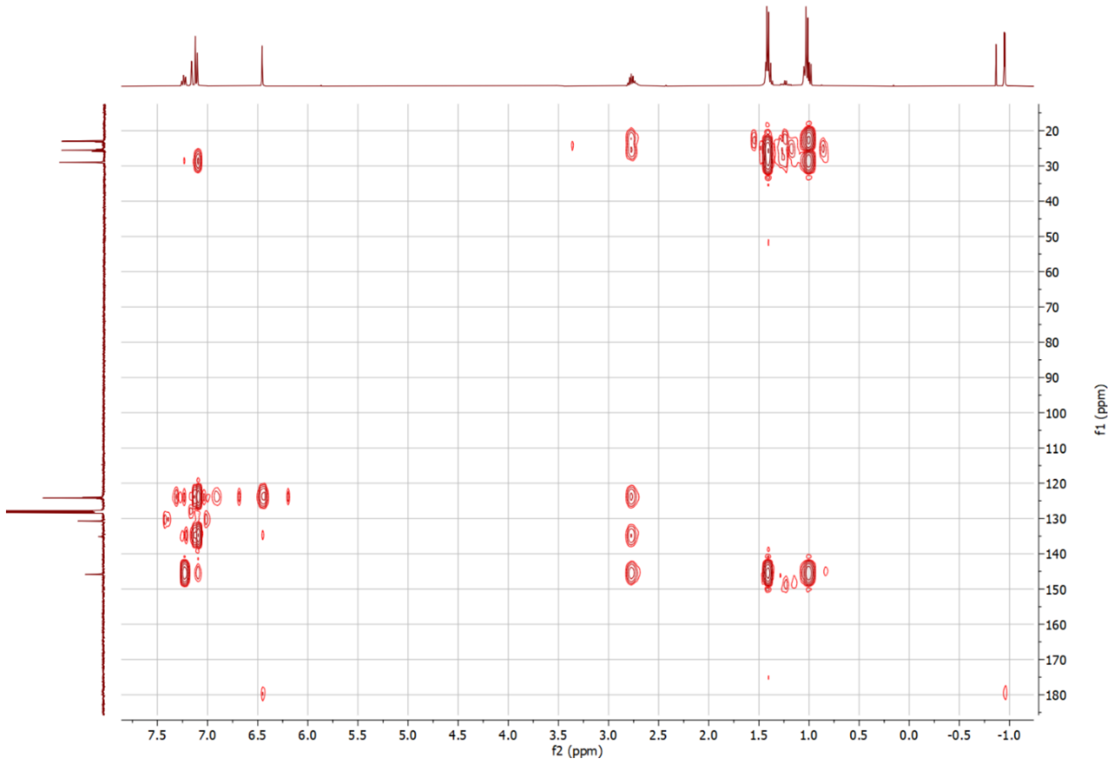

*Figure S28 - ^{1}H-^{13}C HMBC NMR spectrum of **6a** and **6a'**.*

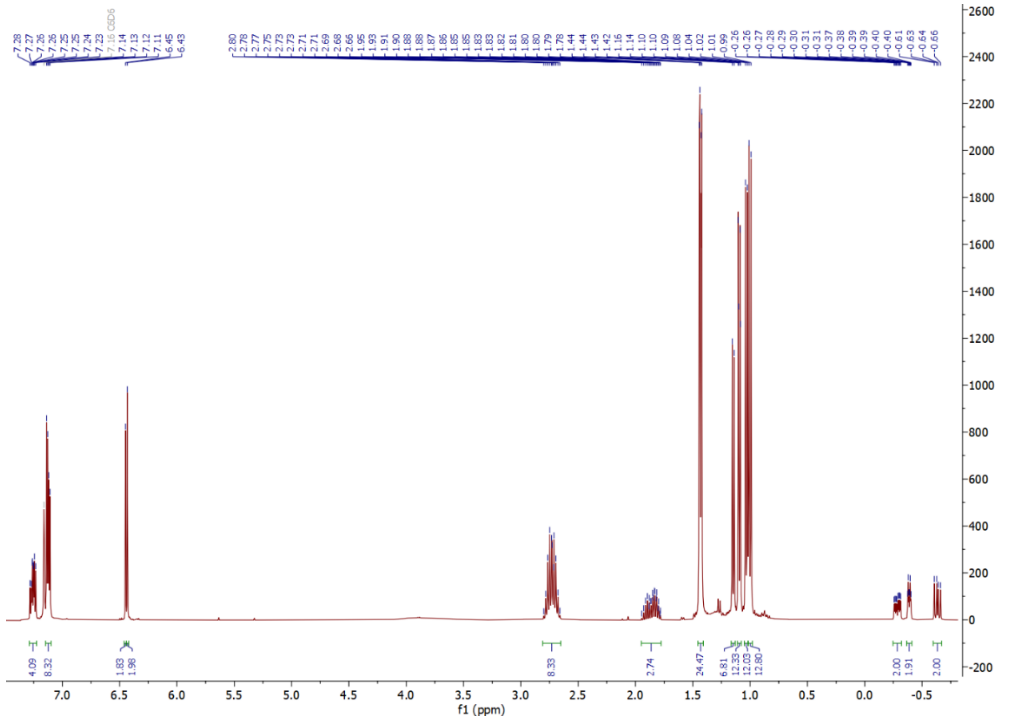

*Figure S29 - ^{1}H NMR spectrum of IDipAliBu$_2$H **7a** and IDipAliBuH$_2$ **7a'**.*

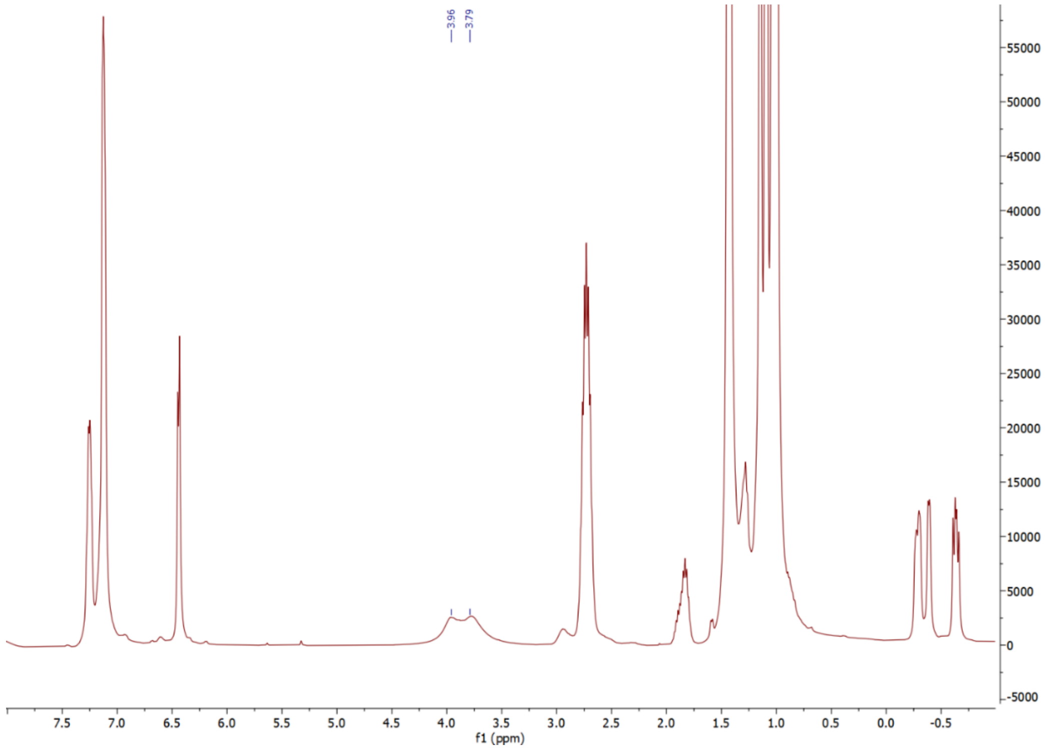

*Figure S30 - $^{1}H\{^{27}Al\}$ NMR spectrum of **7a** and **7a'**.*

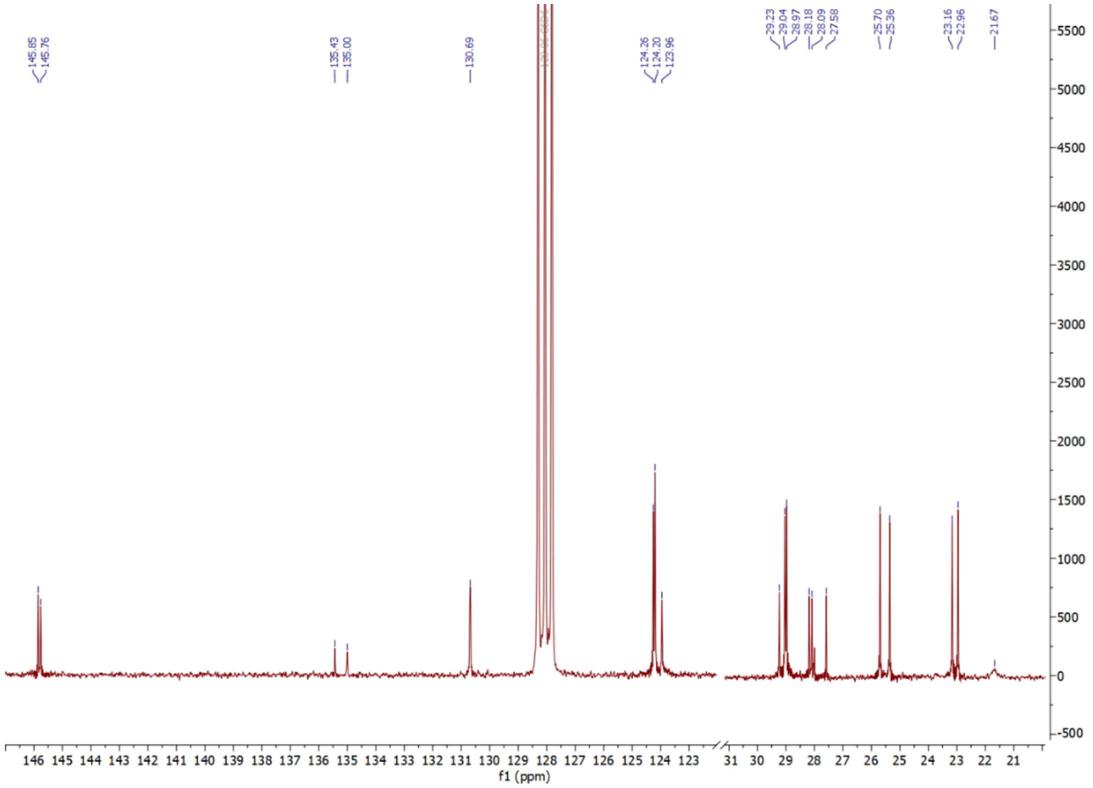

*Figure S31 - $^{13}C\{^{1}H\}$ NMR spectrum of **7a** and **7a'**.*

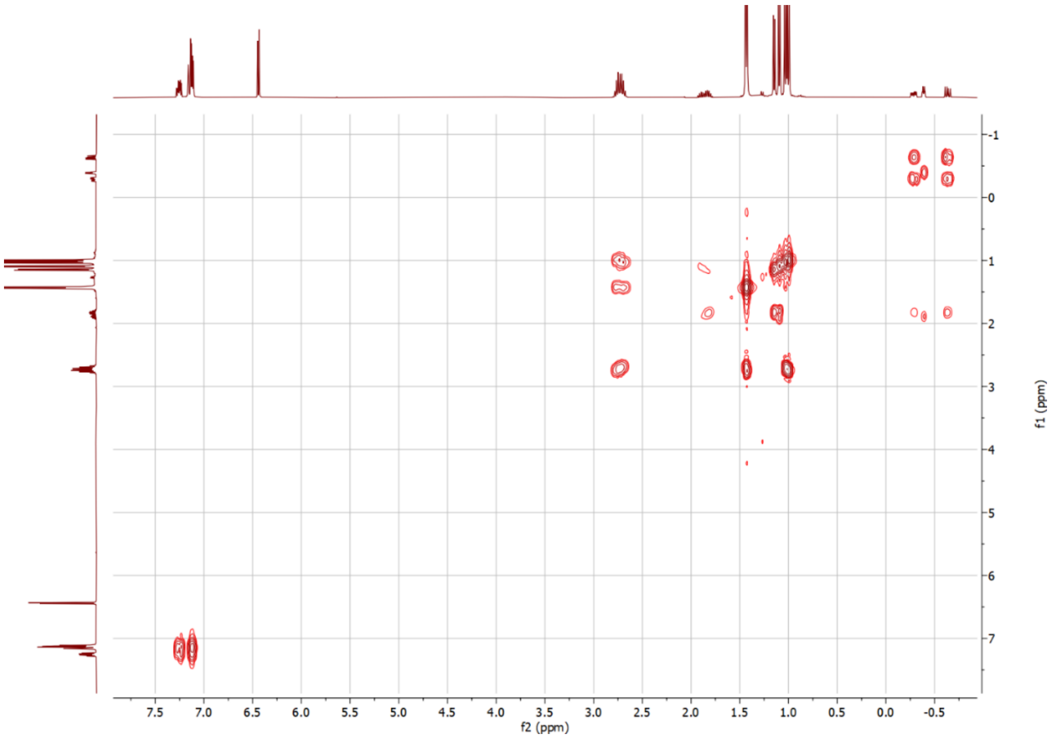

*Figure S32 - ^{1}H-^{1}H COSY NMR spectrum of **7a** and **7a'**.*

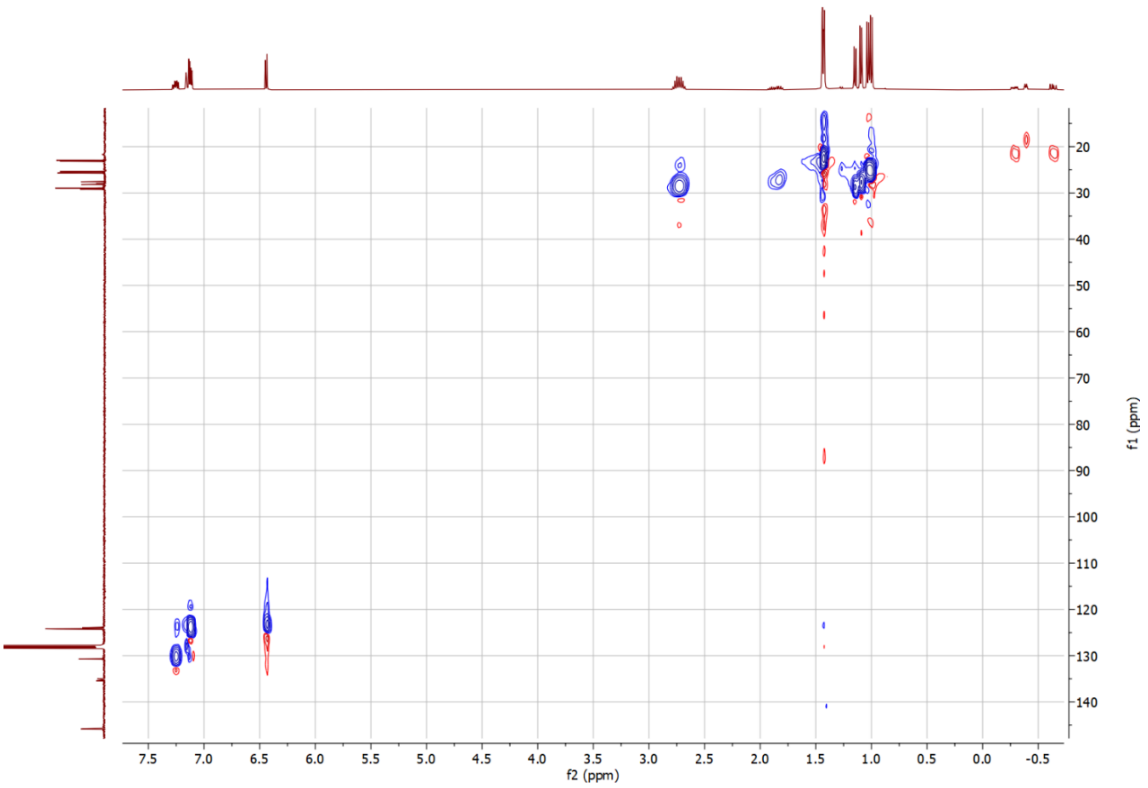

*Figure S33 - ^{1}H-^{13}C HSQC NMR spectrum of **7a** and **7a'**.*

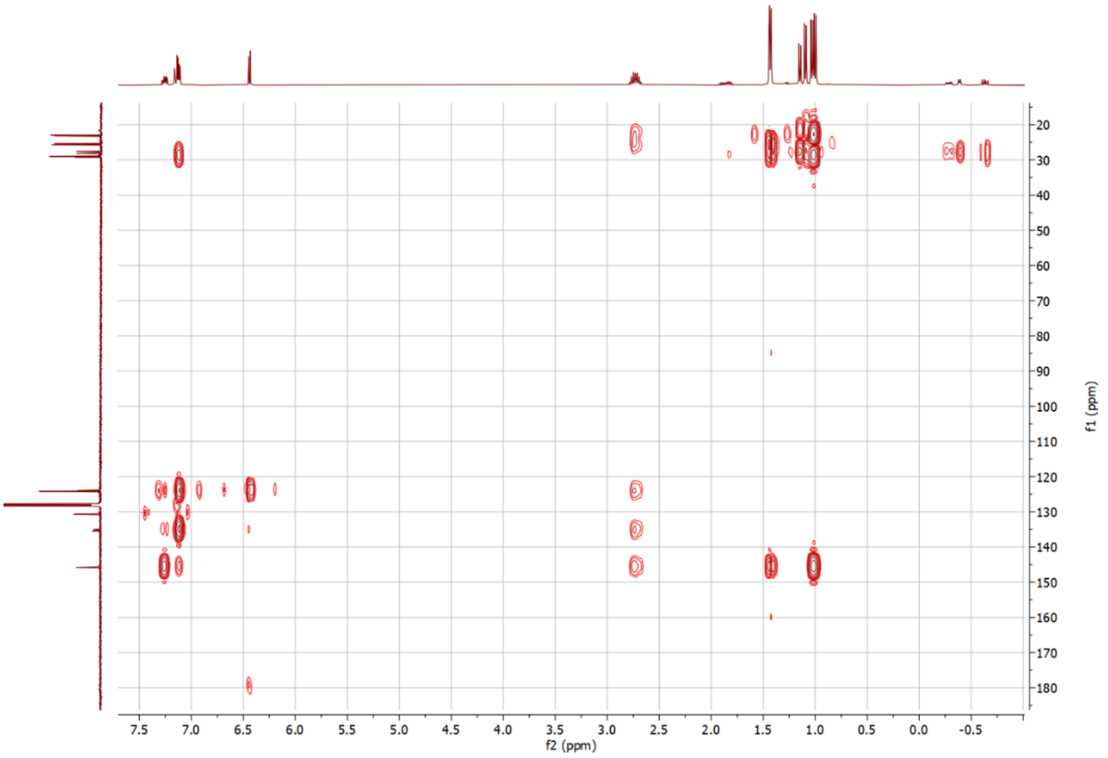

*Figure S34 - ^{1}H-^{13}C HMBC NMR spectrum of **7a** and **7a'**.*

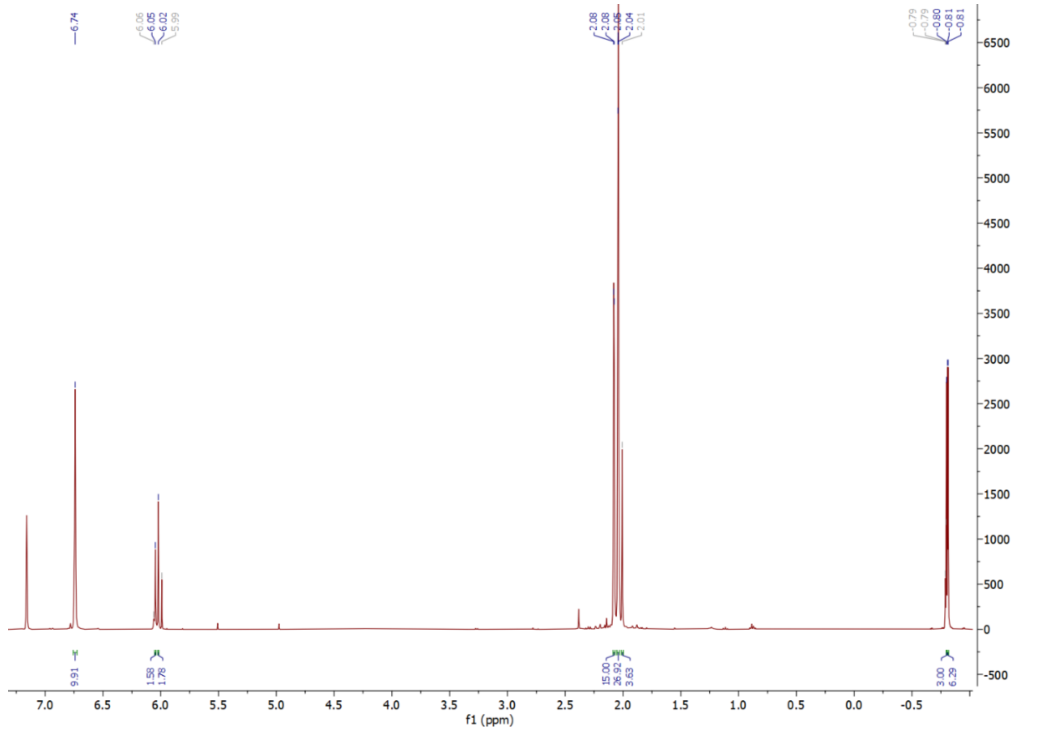

Figure S35 - ^{1}H NMR spectrum of IMesAlMe$_2$H ***6b*** *and IMesAlMeH$_2$* ***6b'****. Starting material resonances labelled in grey. Integral error is a result of overlapping starting material resonances.*

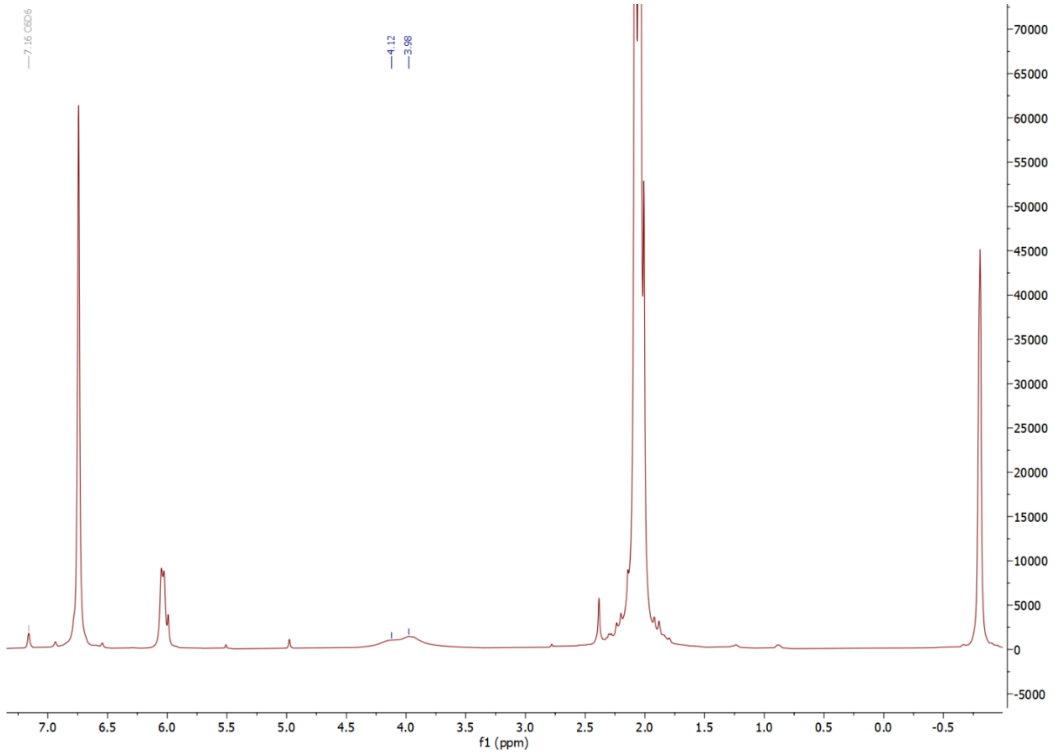

Figure S36 - ^{1}H{^{27}Al} NMR spectrum of ***6b*** *and* ***6b'****.*

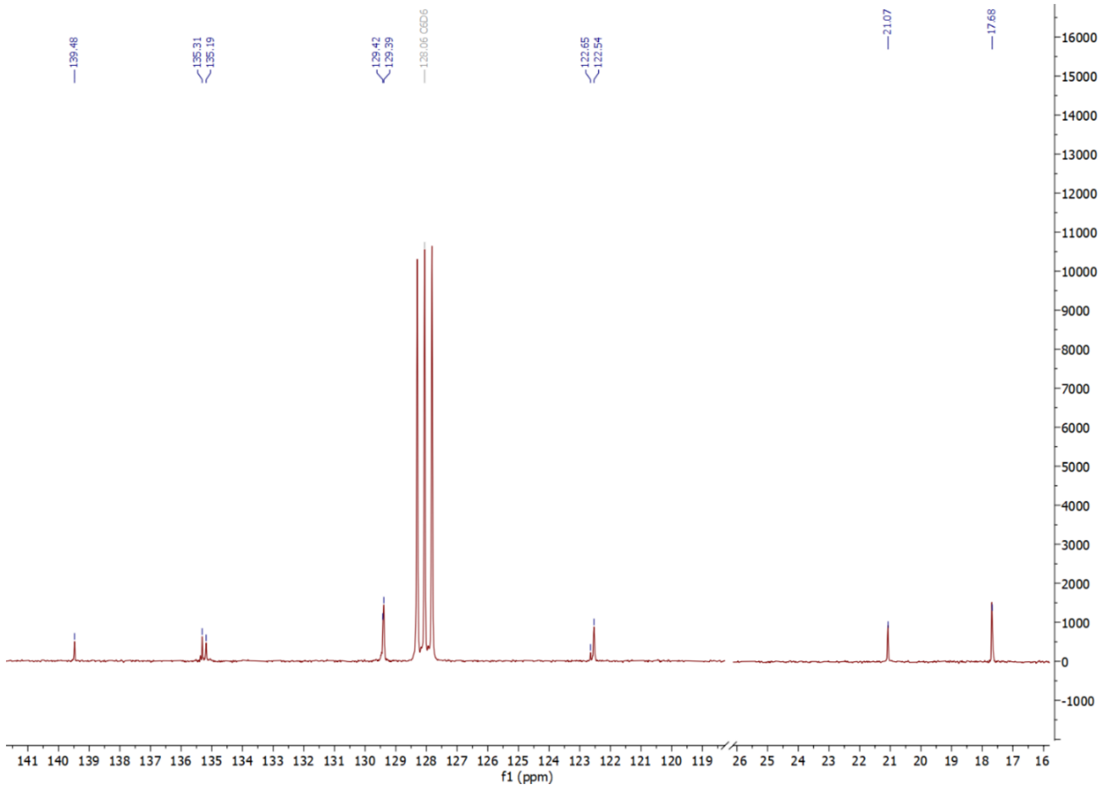

*Figure S37 - $^{13}C\{^1H\}$ NMR spectrum of **6b** and **6b'**.*

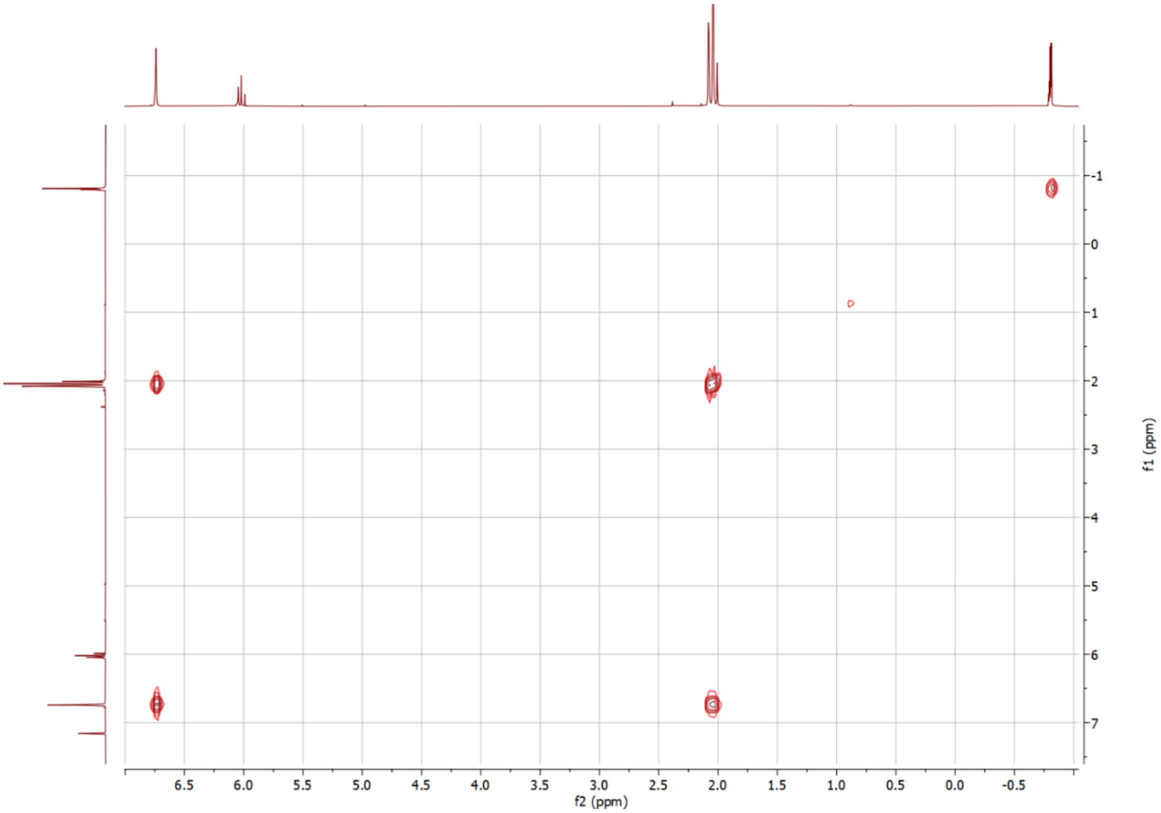

*Figure S38 - ^1H-^1H COSY NMR spectrum of **6b** and **6b'**.*

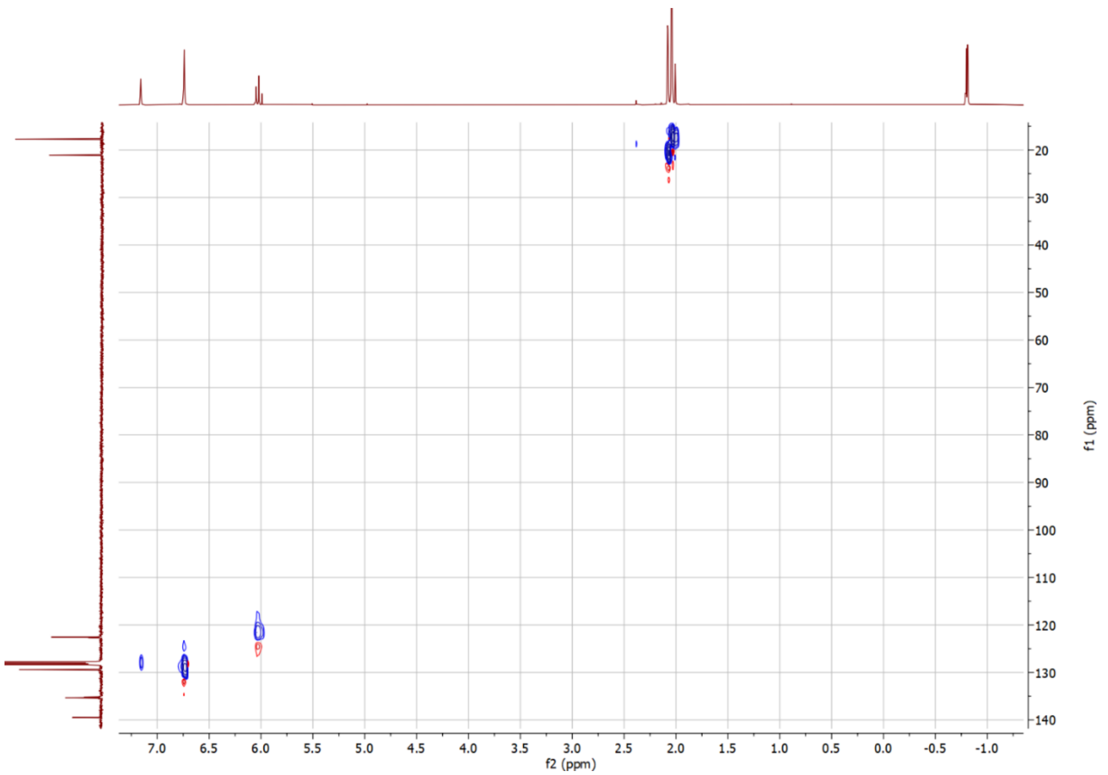

*Figure S39 - ^{1}H-^{13}C HSQC NMR spectrum of **6b** and **6b'**.*

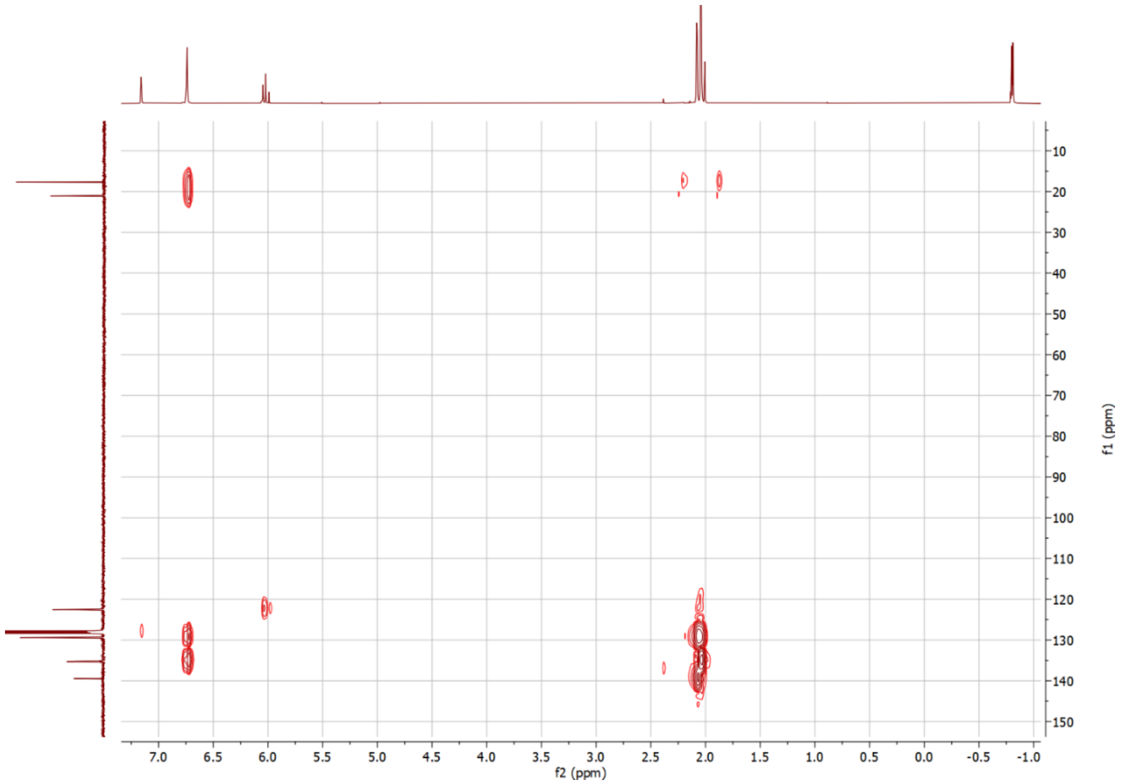

*Figure S40 - ^{1}H-^{13}C HMBC NMR spectrum of **6b** and **6b'**.*

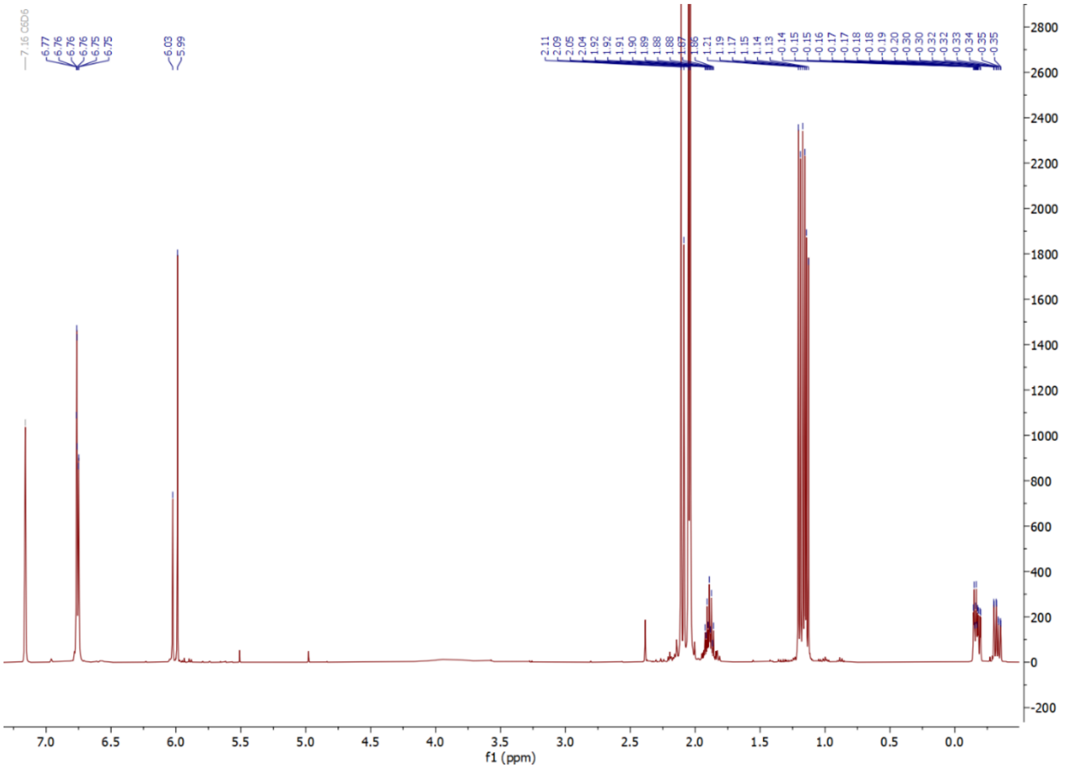

*Figure S41 - ^{1}H NMR spectrum of IMesAliBu$_2$H **7b** and IMesAliBuH$_2$ **7b'**. No integrals provided due to resonance overlap preventing accurate assignment.*

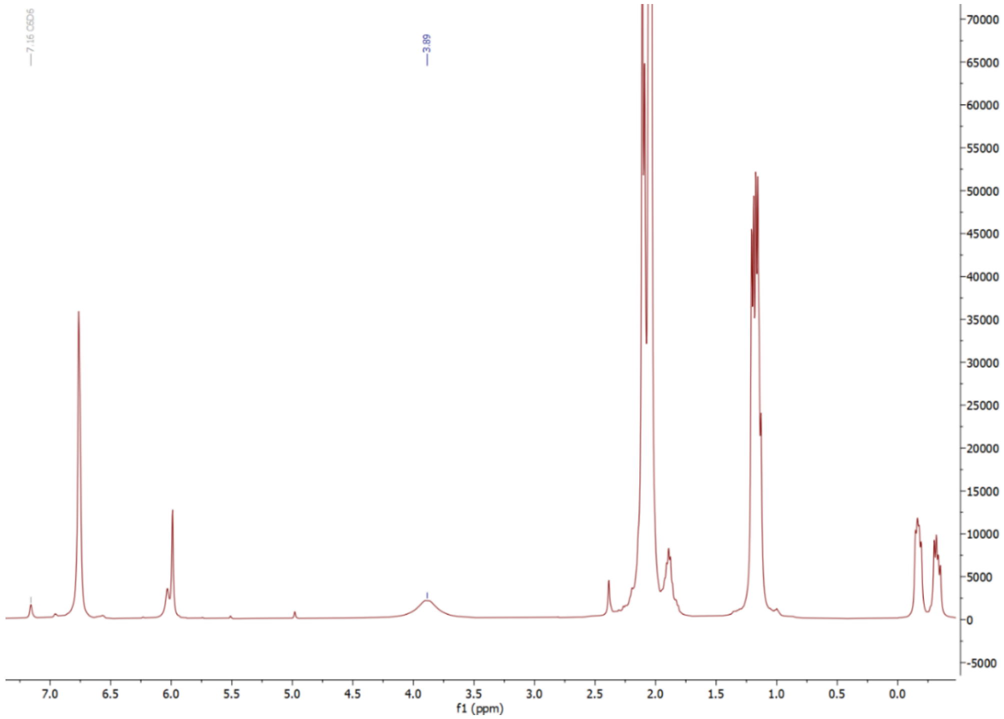

*Figure S42 - $^{1}H\{^{27}Al\}$ NMR spectrum of **7b** and **7b'**.*

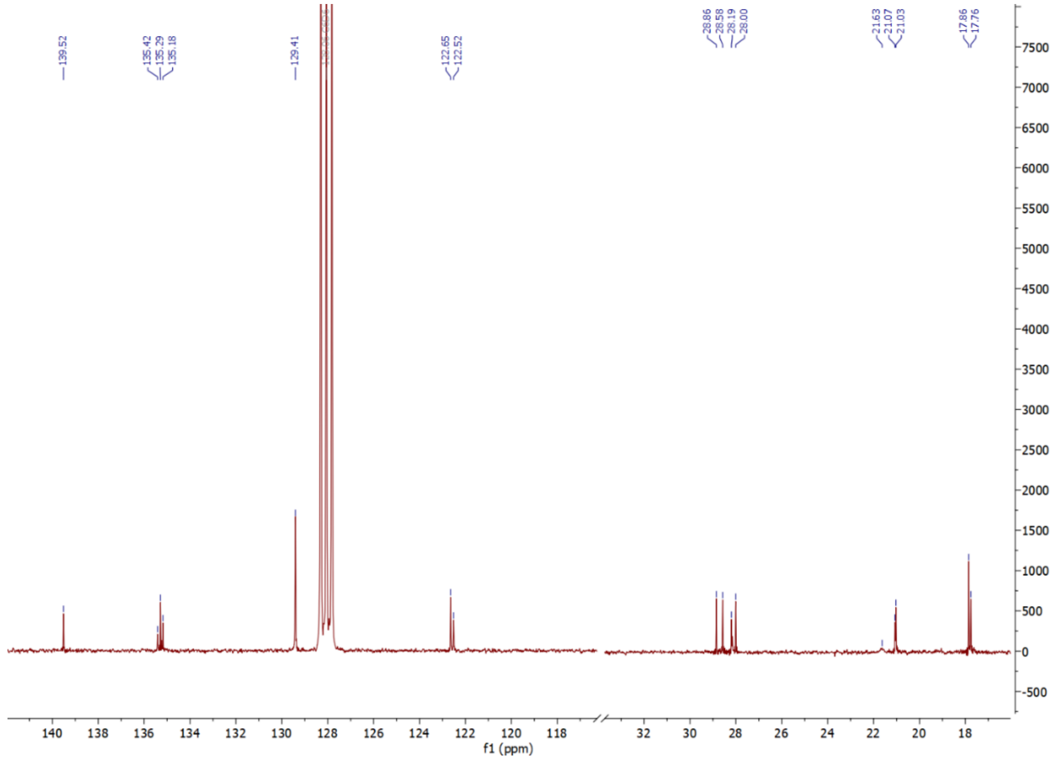

*Figure S43 - $^{13}C\{^{1}H\}$ NMR spectrum of **7b** and **7b'**.*

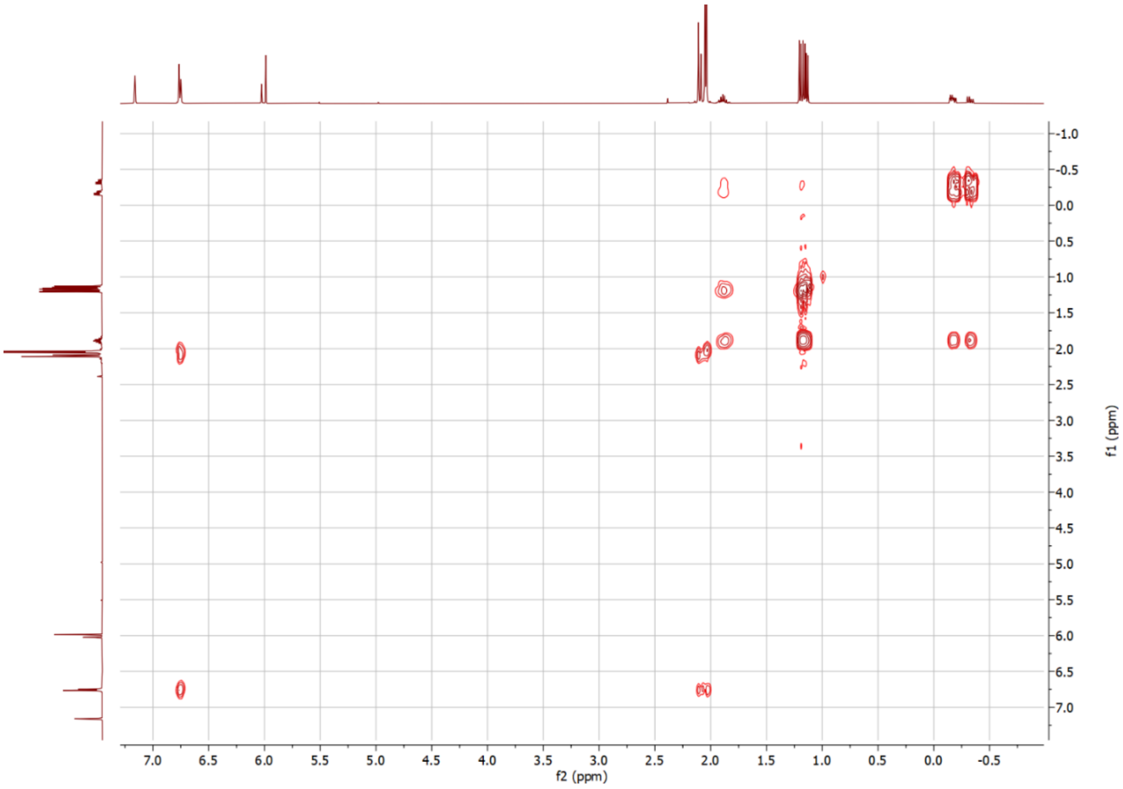

*Figure S44 - ^{1}H-^{1}H COSY NMR spectrum of **7b** and **7b'**.*

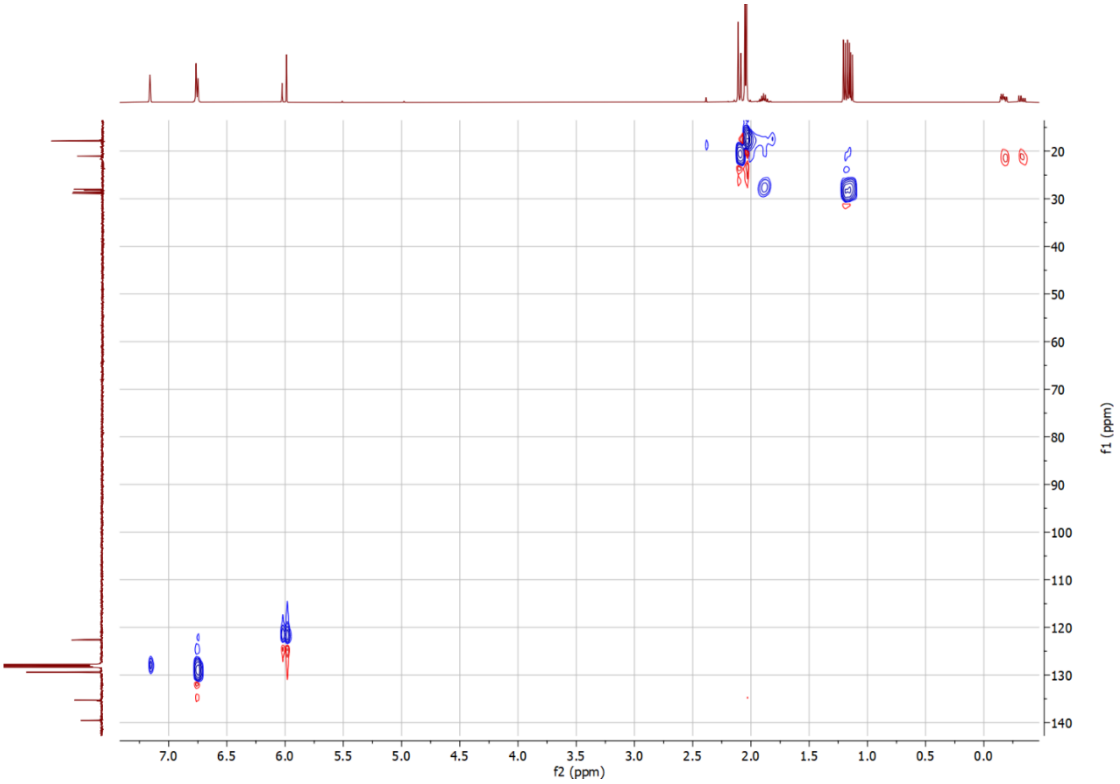

*Figure S45 - ^{1}H-^{13}C HSQC NMR spectrum of **7b** and **7b'**.*

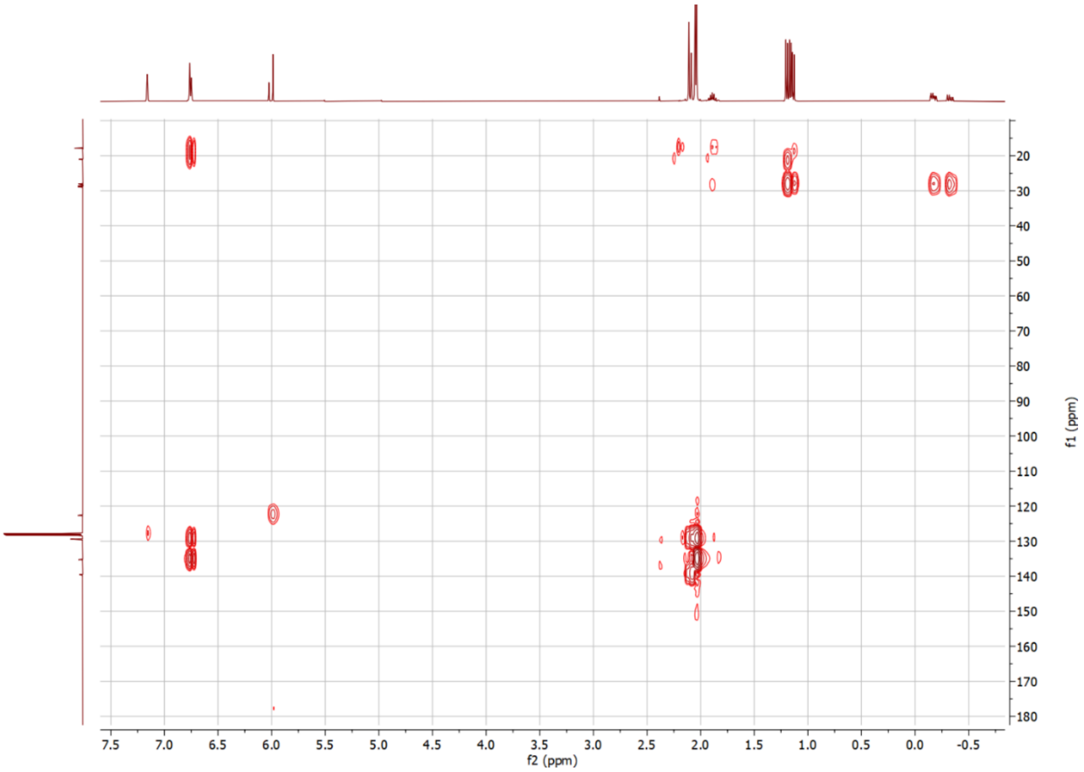

*Figure S46 - ^{1}H-^{13}C HMBC NMR spectrum of **7b** and **7b'**.*

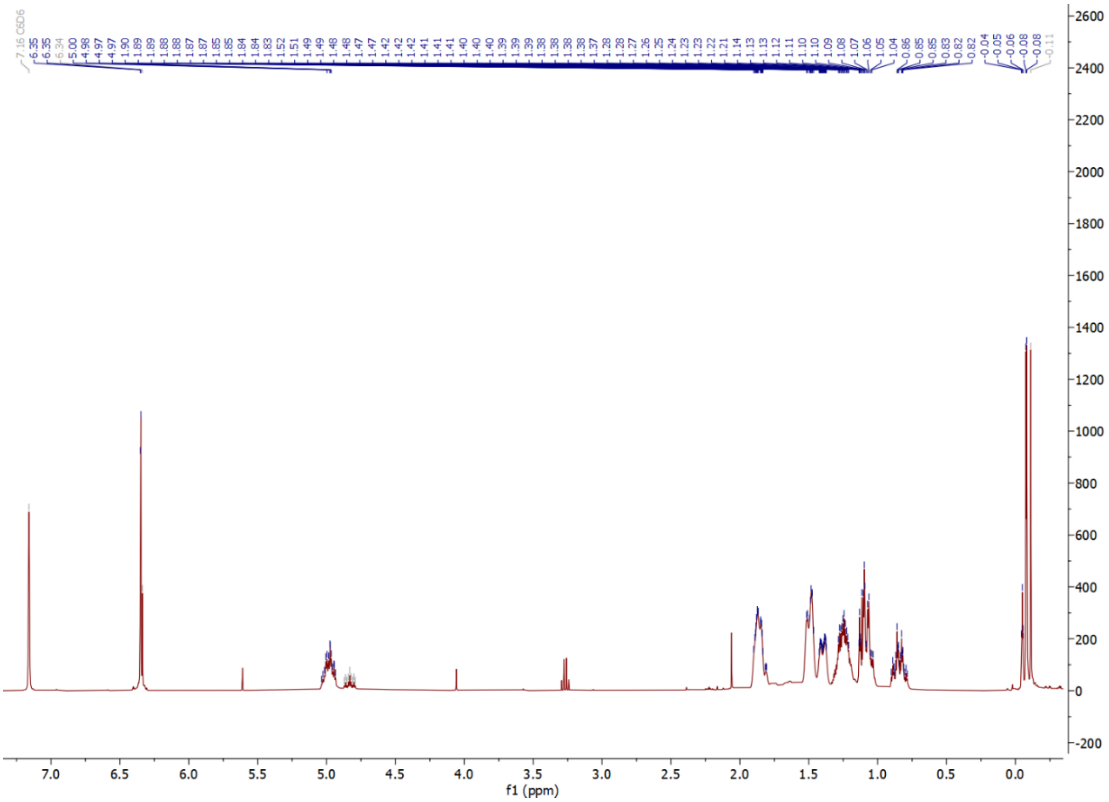

*Figure S47 - ^{1}H NMR spectrum of ICyAlMe$_2$H **6c** and ICyAlMeH$_2$ **6c'**. Starting material resonances labelled in grey. No integrals provided due to resonance overlap preventing accurate assignment.*

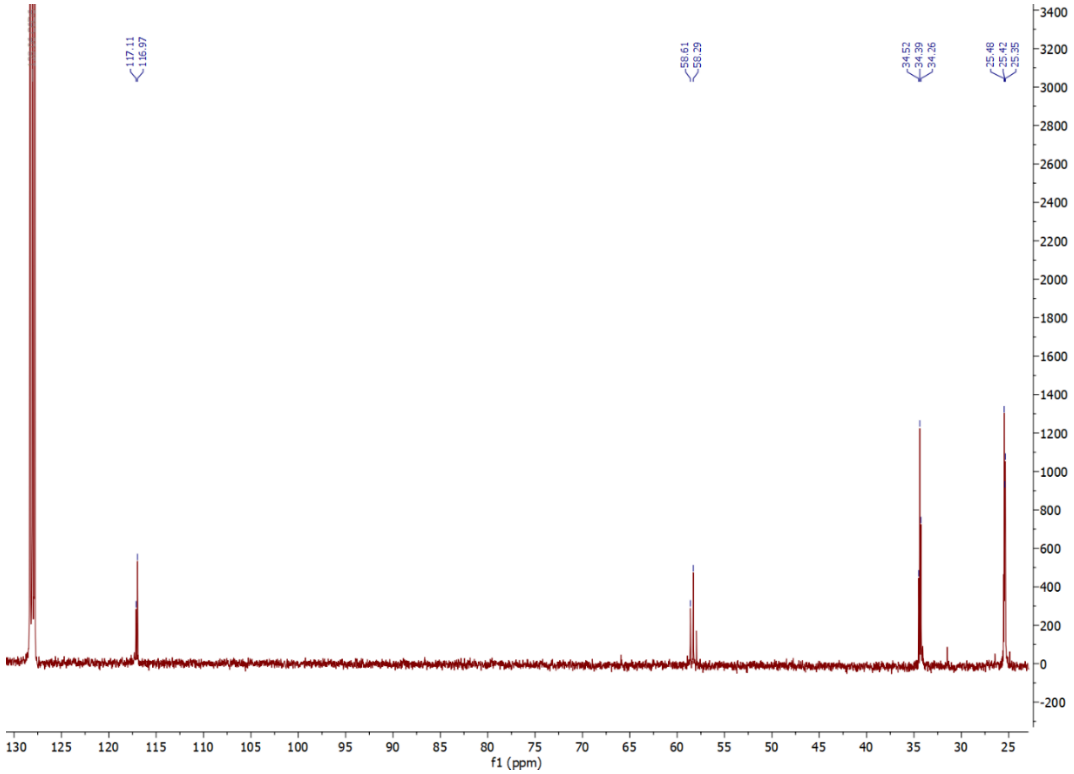

*Figure S48 - ^{13}C{^{1}H} NMR spectrum of **6c** and **6c'**.*

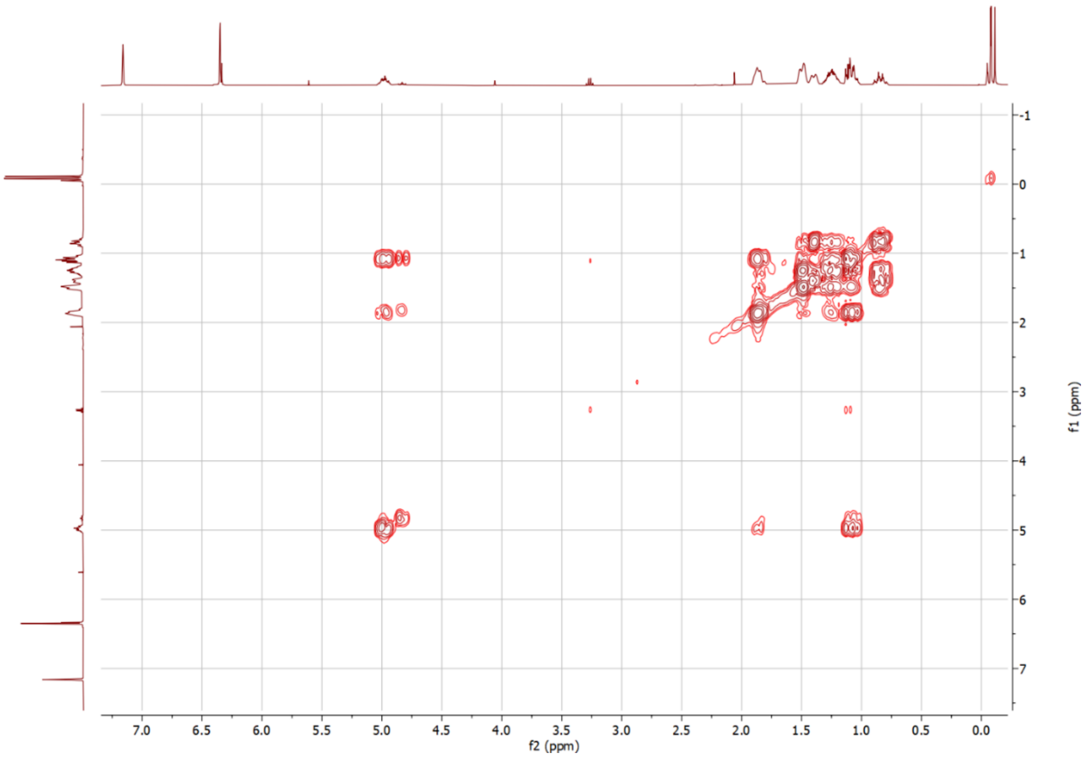

*Figure S49 - ^{1}H-^{1}H COSY NMR spectrum of **6c** and **6c'**.*

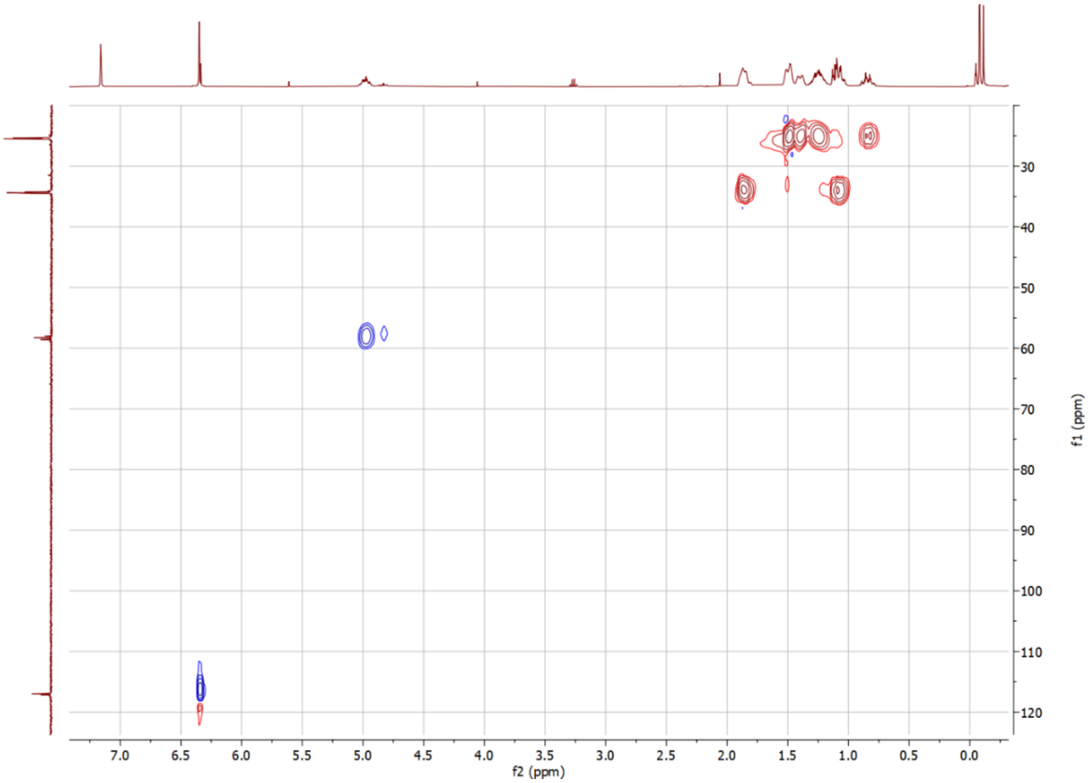

*Figure S50 - ^{1}H-^{13}C HSQC NMR spectrum of **6c** and **6c'**.*

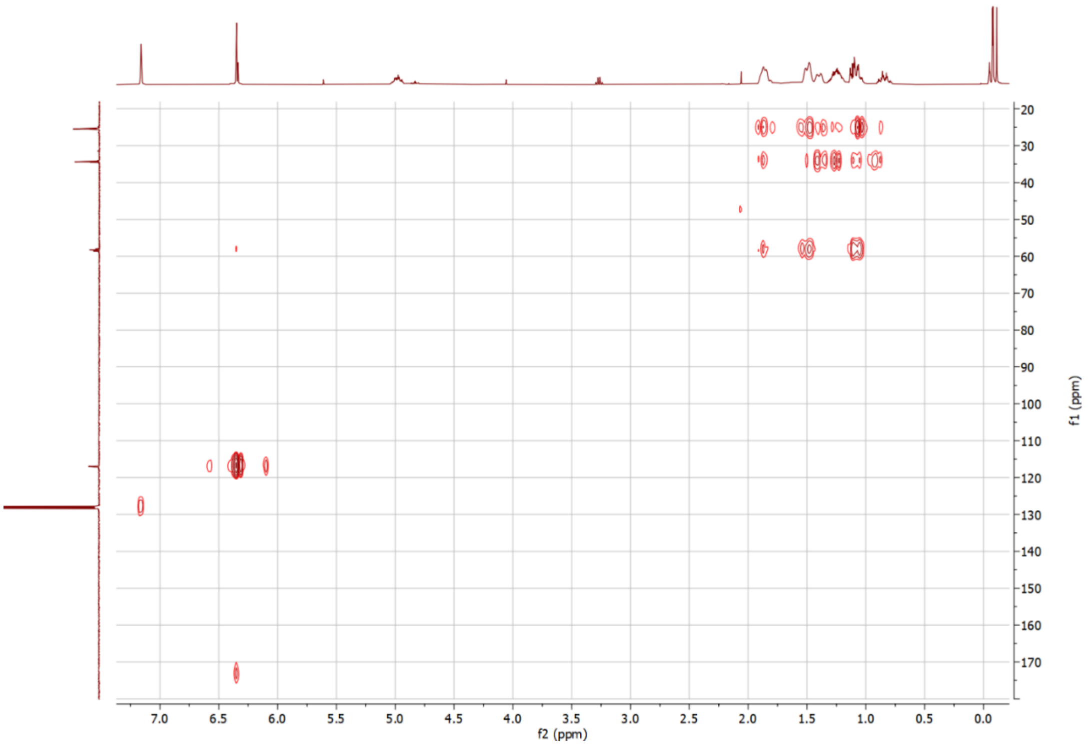

*Figure S51 - ^{1}H-^{13}C HMBC NMR spectrum of **6c** and **6c'**.*

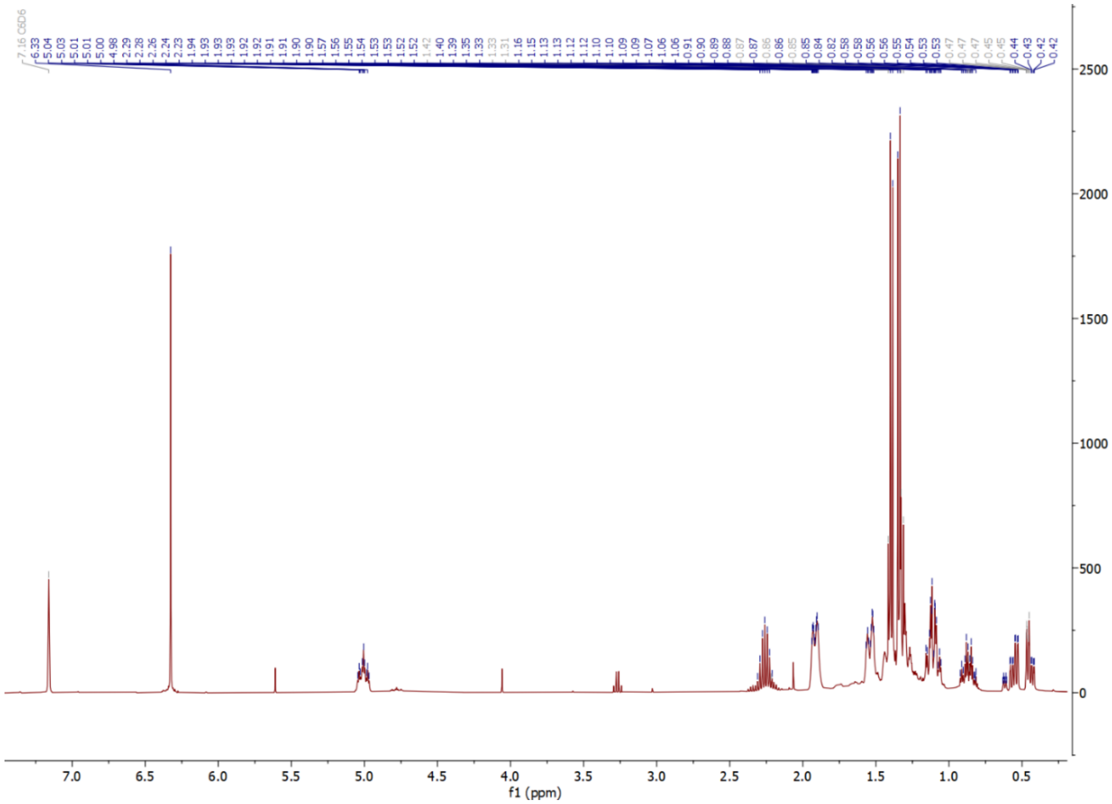

*Figure S52 - ^{1}H NMR spectrum of ICyAliBu$_2$H **7c** and ICyAliBuH$_2$ **7c'**. Starting material resonances labelled in grey. No integrals provided due to resonance overlap preventing accurate assignment.*

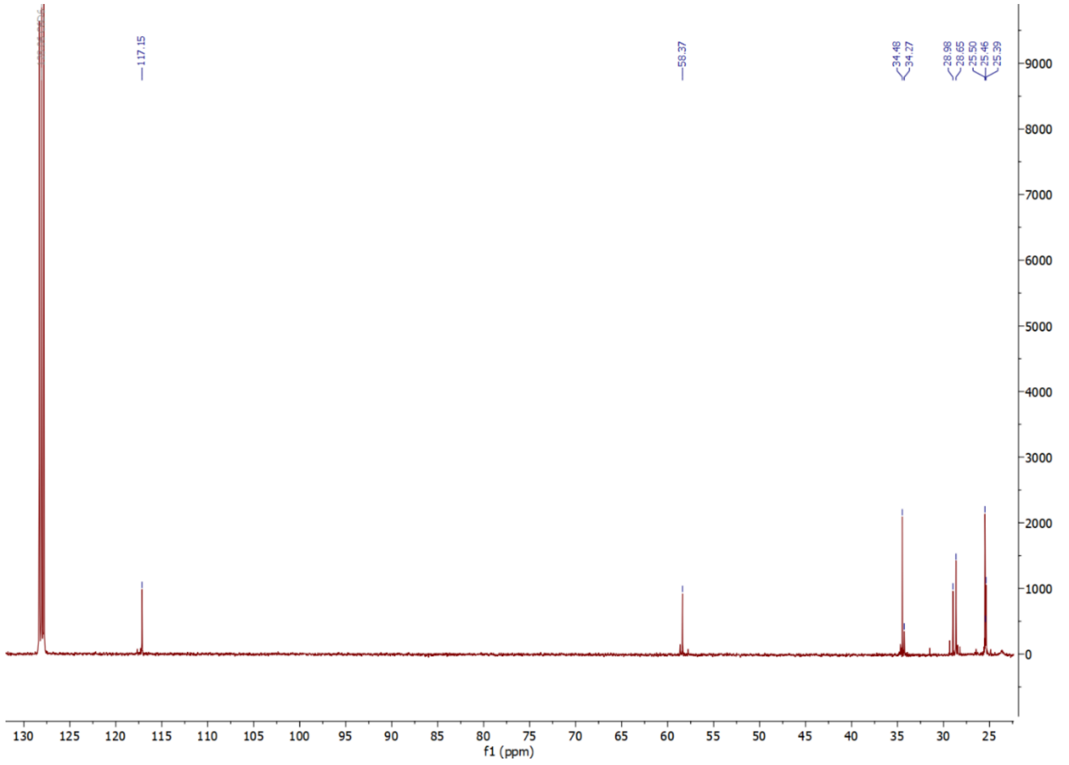

*Figure S53 - $^{13}C\{^{1}H\}$ NMR spectrum of **7c** and **7c'**.*

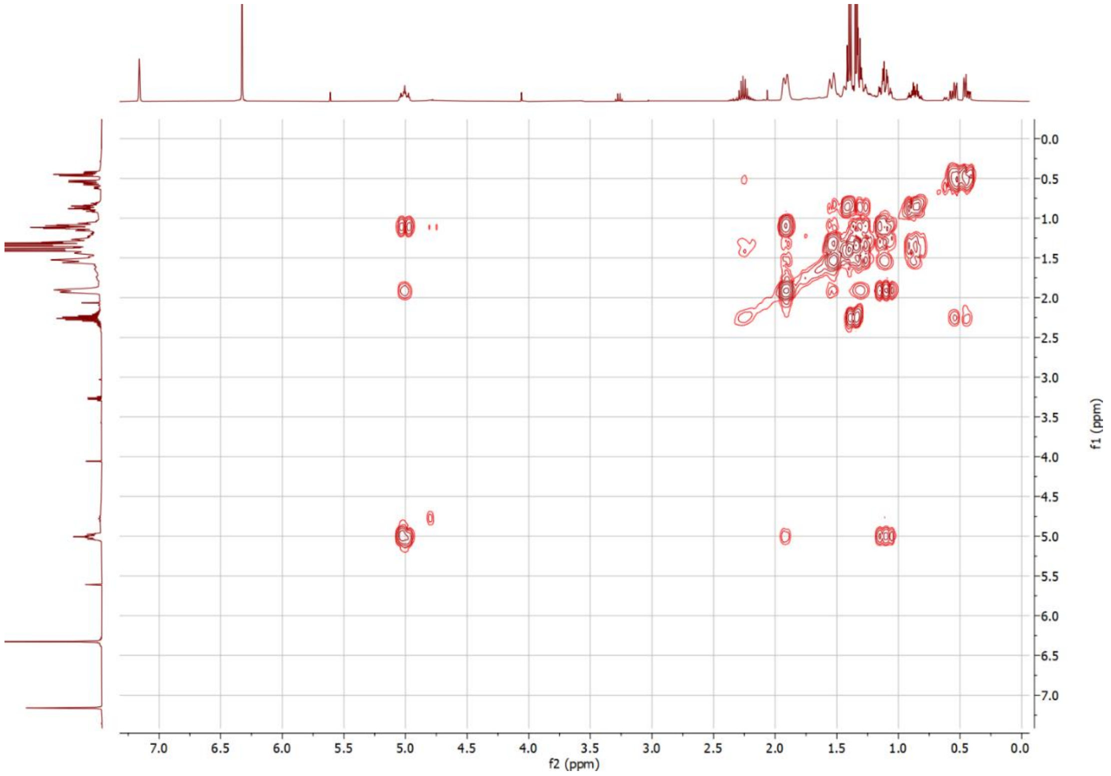

*Figure S54 - ^{1}H-^{1}H COSY NMR spectrum of **7c** and **7c'**.*

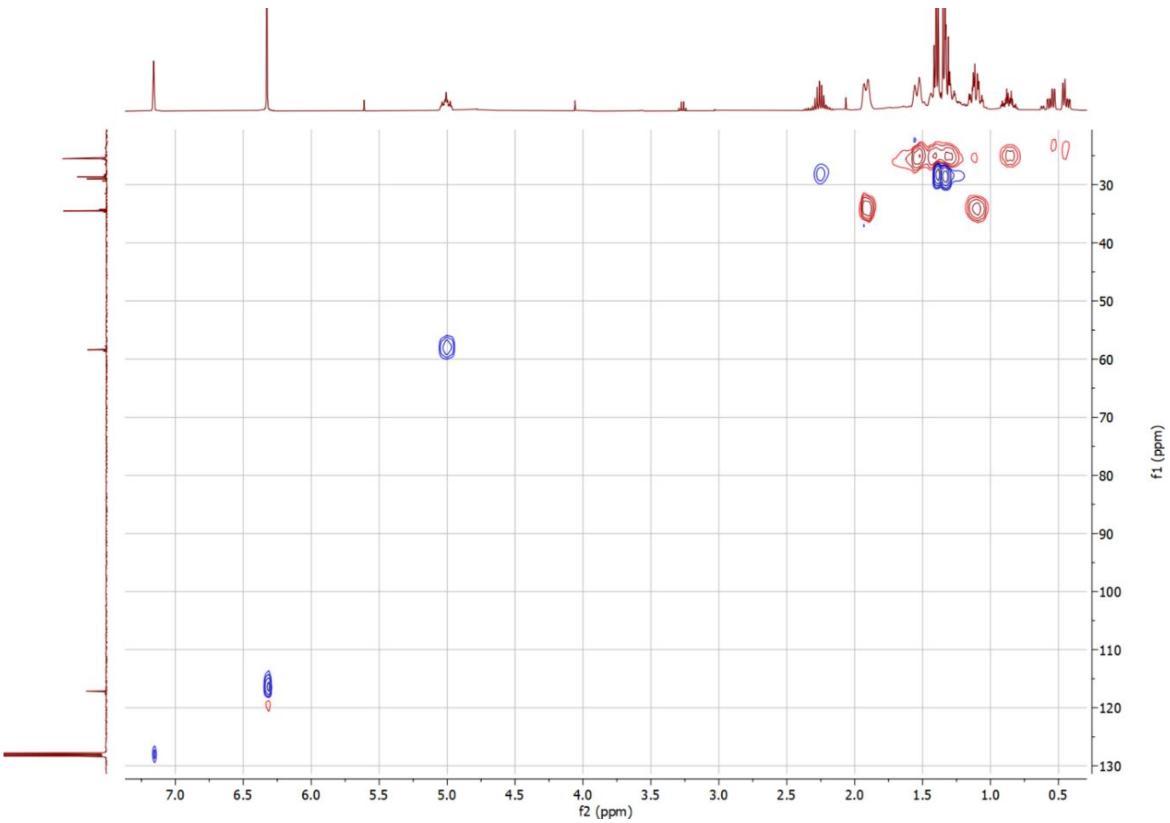

*Figure S55 - ^{1}H-^{13}C HSQC NMR spectrum of **7c** and **7c'**.*

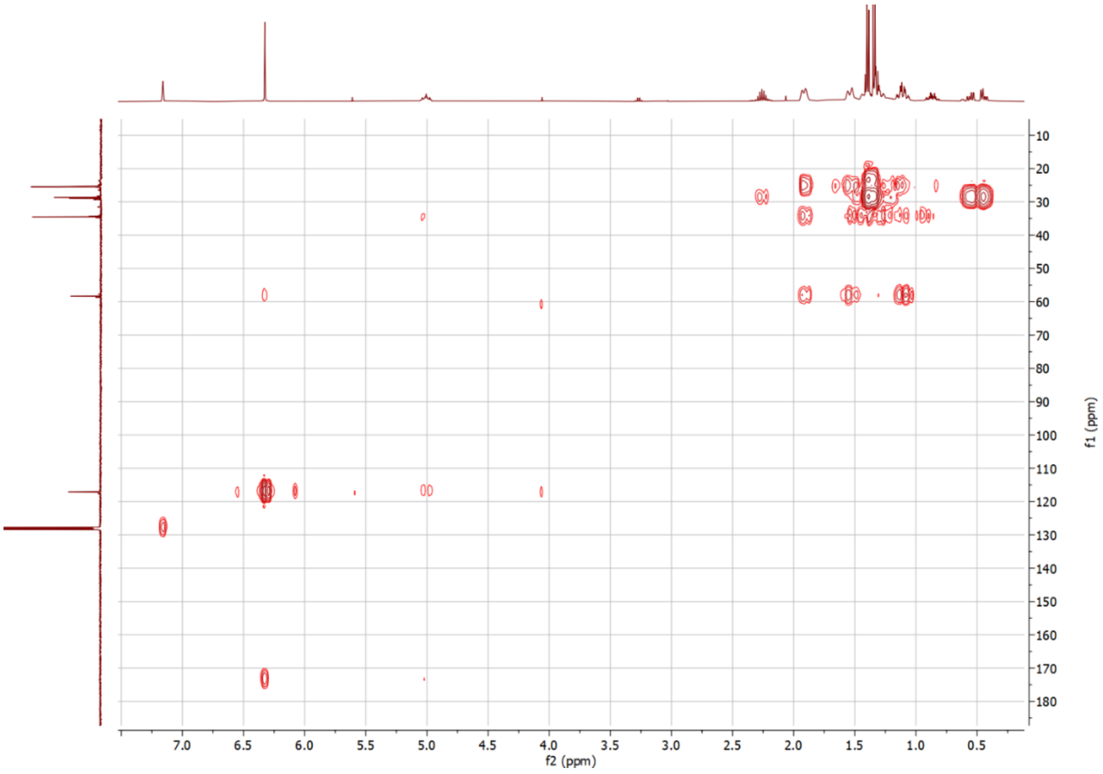

*Figure S56 - ^{1}H-^{13}C HMBC NMR spectrum of **7c** and **7c'**.*

4. X-Ray Crystallography Data

Crystallographic data was measured with a Rigaku Synergy-i instrument with monochromated Cu−Kα (λ 1.54184 Å) radiation. The measured data was processed with the CrysAlisPro software package.[8] The structures were solved with the ShelXT structure solution program and refined with ShelXL-2018 to convergence against F^2.[9] Final refinement was within OLEX or WinGX.[10,11] All ordered non-hydrogen atoms were refined using anisotropic thermal parameters and the hydride H atom positions were refined freely. The butyl ligands of **5c**, a xylene group of **6a**, and a propyl substituent of **7a** were modelled as disordered over two positions. We note that for **6a** this disorder affects a large proportion of the molecule and thus induces associated limitations on the accuracy of the model. Here the disorder corresponds to the xylene ring lying slightly above and slightly below the plane of the C_4N ring with refined occupancies of 0.794(6) and 0.206(6). Appropriate constraints and restraints were added to these disordered groups to force approximation to expected geometries and displacement behaviours. Selected crystallographic and refinement data is given in Table S1.

Full details of single crystal diffraction data for are reported in crystallographic information files (CIF) accompanying this document and deposited with the CCDC as deposition numbers 2495126 to 2495131. Full details on data collection, reduction and refinement can be found in the individual CIFs.

Table S1 – Selected crystallographic and refinement parameters.

Compound	4c	5b	5c
CCDC no.	2495126	2495127	2495128
Empirical formula	$AlN_2C_{18}H_{33}$	$AlN_2C_{33}H_{51}$	$AlN_2C_{27}H_{51}$
Formula weight	304.44	502.73	430.67
Temperature/K	100(2)	100(2)	200(2)
Crystal system	monoclinic	monoclinic	orthorhombic
Space group	$P2_1/c$	$P2_1/n$	$P2_12_12_1$
a/Å	16.1811(6)	8.8753(1)	10.8582(1)
b/Å	13.2684(4)	22.3197(1)	11.0533(1)
c/Å	18.4971(6)	16.0290(1)	24.1829(2)
α/°	90	90	90
β/°	104.267(4)	90.162(1)	90
γ/°	90	90	90
Volume/$Å^3$	3848.8(2)	3175.24(4)	2902.41(4)
Z	8	4	4
ρ_{calc}g/cm^3	1.051	1.052	0.986
μ/mm^{-1}	0.874	0.7	0.693
Crystal size/mm^3	0.16 × 0.14 × 0.07	0.21 × 0.11 × 0.09	0.16 × 0.11 × 0.08
2Θ range for data collection/°	5.636 to 144.802	6.79 to 143.85	7.312 to 146.566
Reflections collected	76625	60313	40422
Independent reflections	7468 [R_{int} = 0.1178, R_{sigma} = 0.0550]	6193 [R_{int} = 0.0382, R_{sigma} = 0.0170]	5829 [R_{int} = 0.0278, R_{sigma} = 0.0158]
Restraints/parameters	0/385	0/334	12/326
Reflections obs	4836	5655	5632
Goodness-of-fit on F^2	1.019	1.060	1.050
Final R indexes [I>=2σ (I)]	R_1 = 0.0689, wR_2 = 0.1679	R_1 = 0.0364, wR_2 = 0.0989	R_1 = 0.0302, wR_2 = 0.0830
Final R indexes [all data]	R_1 = 0.1081, wR_2 = 0.1917	R_1 = 0.0396, wR_2 = 0.1011	R_1 = 0.0314, wR_2 = 0.0838
Largest diff. peak/hole/e Å^{-3}	0.438/-0.330	0.301/-0.243	0.180/-0.128

Table S1 (cont.) – Selected crystallographic and refinement parameters.

Compound	6a	7a	7c
CCDC no.	2495129	2495130	2495131
Empirical formula	$AlN_2C_{29}H_{43}$	$AlN_2C_{35}H_{55}$	$AlN_2C_{23}H_{43}$
Formula weight	446.63	530.79	374.57
Temperature/K	101(2)	100(2)	150(2)
Crystal system	monoclinic	monoclinic	Monoclinic
Space group	$P2_1/c$	$P2_1/c$	P/c
a/Å	18.8569(2)	20.6968(8)	12.7143(12)
b/Å	9.6665(1)	16.0699(5)	8.2473(5)
c/Å	15.6154(2)	20.6070(8)	23.447(2)
α/°	90	90	90
β/°	93.976(1)	101.851(4)	90.321(9)
γ/°	90	90	90
Volume/Å^3	2839.53(6)	6707.7(4)	2458.6(3)
Z	4	8	4
ρ_{calc}g/cm^3	1.045	1.051	1.012
μ/mm^{-1}	0.733	0.686	0.758
Crystal size/mm^3	0.25 × 0.2 × 0.17	0.20 × 0.14 × 0.09	0.13 × 0.11 × 0.05
2Θ range for data collection/°	4.698 to 139.986	7.022 to 139.988	6.952 to 133.994
Reflections collected	35256	131349	27254
Independent reflections	5376 [R_{int} = 0.0677, R_{sigma} = 0.0284]	12723 [R_{int} = 0.1264, R_{sigma} = 0.0587]	8096 [R_{int} = 0.1235, R_{sigma} = 0.1244]
Restraints/parameters	92/332	12/725	2/483
Reflections obs	4748	7618	3856
Goodness-of-fit on F^2	1.050	1.016	1.018
Final R indexes [I>=2σ (I)]	R_1 = 0.0698, wR_2 = 0.1962	R_1 = 0.0824, wR_2 = 0.2126	R_1 = 0.0890, wR_2 = 0.2262
Final R indexes [all data]	R_1 = 0.0756, wR_2 = 0.2023	R_1 = 0.1335, wR_2 = 0.2526	R_1 = 0.1784, wR_2 = 0.3028
Largest diff. peak/hole/e Å^{-3}	1.102/-0.689	0.727/-0.247	0.347/-0.647

5. References

(1) Ortega, N.; Richter, C.; Glorius, F. N-Formylation of Amines by Methanol Activation. *Org. Lett.* **2013**, *15*, 1776–1779.

(2) Arduengo, A. J.; Dias, H. V. R.; Harlow, R. L.; Kline, M. Electronic Stabilization of Nucleophilic Carbenes. *J. Am. Chem. Soc.* **1992**, *114*, 5530–5534.

(3) Jafarpour, L.; Stevens, E. D.; Nolan, S. P. A Sterically Demanding Nucleophilic Carbene: 1,3-Bis(2,6-Diisopropylphenyl)Imidazol-2-Ylidene). Thermochemistry and Catalytic Application in Olefin Metathesis. *J. Organomet. Chem.* **2000**, *606*, 49–54.

(4) Murphy, F.; Kennedy, A. R.; Weetman, C. E. Synthesis and Structural Comparisons of NHC-Alanes. *Inorganics* **2022**, *11*, 13.

(5) Wu. M, M.; Gill, A. M.; Yunpeng, L.; Falivene, L.; Yongxin, L.; Ganguly, R.; Cavallo, L.; García, F. Synthesis, Structural Studies and Ligand Influence on the Stability of Aryl-NHC Stabilised Trimethylaluminium Complexes. *Dalton Trans.* **2015**, *44*, 15166–15174.

(6) Baker, R. J.; Davies, A. J.; Jones, C.; Kloth, M. Structural and Spectroscopic Studies of Carbene and N-Donor Ligand Complexes of Group 13 Hydrides and Halides. *J. Organomet. Chem.* **2002**, *656*, 203–210.

(7) Ruff, J. K.; Hawthorne, M. F. The Amine Complexes of Aluminum Hydride. I. *J. Am. Chem. Soc.* **1960**, *82*, 2141–2144.

(8) Agilent (2014). CrysAlis PRO. Agilent Technologies Ltd, Yarnton, Oxfordshire, England. 2014.

(9) Sheldrick, G. M. *Acta Crystallogr. Sect. C Struct. Chem.* 2015, **71**, 3–8.

(10) Dolomanov, O. V.; Bourhis, L. J.; Gildea, R. J.; Howard, J. a. K; Puschmann, H. *J. Appl. Crystallogr.* 2009, **42**, 339–341.

(11) Farrugia, L. J. *J. Appl. Crystallogr.* 2012, **45**, 849-854.
